# Chemical Upcycling of Poly(Lactic Acid) Waste Into Lactyl‐Based Diols for the Synthesis of New Polyesters

**DOI:** 10.1002/cplu.70261

**Published:** 2026-10-11

**Authors:** Federica Santulli, Andrea Annunziata, Pasquale D’Angelo, Marina Lamberti, Leonardo Marchese, Daniela Pappalardo, Mina Mazzeo

**Affiliations:** ^1^ Department of Chemistry and Biology “Adolfo Zambelli” Università degli Studi di Salerno Fisciano Italy; ^2^ Department of Science and Technology Università degli Studi del Sannio Benevento Italy; ^3^ Department of Science and Technological Innovation Università del Piemonte Orientale Alessandria Italy

**Keywords:** alcoholysis, polycondensation reactions, polylactic acid, polymerization, recycling, zinc

## Abstract

The development of efficient catalytic strategies for the chemical recycling and upcycling of poly(lactic acid) (PLA) is of great importance for advancing sustainable polymer technologies and promoting a circular plastics economy. Herein, we describe the selective alcoholysis of PLA promoted by a commercial titanium alkoxide and a phenoxy‐imine pyridine zinc complex using various diols, leading to the formation of novel lactyl‐based intermediates that were employed in subsequent polycondensation reactions with dicarboxylic acids. Alcoholysis reaction of PLA in the presence of ethylene glycol, 1,4‐butanediol, *cis*‐2‐butene‐1,4‐diol and 3‐allyloxy‐1,2‐propanediol afforded novel lactyl‐containing diols, which were successfully reacted with adipic acid to produce a series of novel copolyesters. Structural characterization by one‐ and two‐dimensional NMR spectroscopy confirmed the effective incorporation of lactyl units into the polymer backbones. Thermal analyses revealed that the introduction of lactyl moieties influences the crystallinity and thermal behavior of the resulting materials, generally leading to amorphous polymers. This approach demonstrates a straightforward and versatile route for the valorization of PLA waste into novel functional building blocks for the synthesis of potentially biodegradable polyesters with a variety of chemical features.

## Graphical abstract

We report a green one‐pot synthesis of novel polyesters from postconsumer PLA, diols, and adipic acid derived from renewables, using a cheap, easy‐to‐make titanium‐based catalyst.

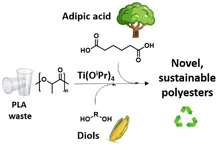

## 1 Introduction

Global plastic production has currently surpassed 420 million tons per year and is projected to double by 2050 [1]. Despite this rapid growth, end‐of‐life management strategies for plastic waste have not advanced at a comparable rhythm. Recycling rates remain below 10% [2, 3], and more than 90% of plastics become waste after use [4, 5]. Moreover, the plastics industry remains heavily dependent on fossil resources, with petroleum‐based feedstocks accounting for approximately 99% of production. This scenario is further aggravated by a predominantly linear “take–use–dispose” model, which accelerates resource depletion and environmental degradation. Therefore, a transition toward a sustainable circular economy for plastics is essential [6, 7, 8, 9].

The development of bio‐based and biodegradable polymers represents a key strategy for promoting a more sustainable plastics economy [10, 11, 12, 13, 14, 15, 16, 17]. Among these, poly(lactic acid) (PLA) is particularly attractive due to its renewable origin, ease of processing, versatility, and potential for chemical recyclability. PLA can be synthesized either from lactic acid or from its cyclic dimer, lactide. Lactic acid is typically produced via starch fermentation and is associated with a relatively low carbon footprint [18].

Although PLA is not readily degradable under ambient environmental conditions, it is fully compostable under industrial conditions [19]. However, life cycle assessment (LCA) studies indicate that the most energy‐intensive stage in PLA production is the conversion of biomass into L‐lactic acid. This suggests that composting, which requires regeneration of the original feedstock, may not be the most advantageous end‐of‐life option [20].

Similarly, mechanical recycling often leads to a reduction in material quality, resulting in economic drawbacks [21]. Consequently, alternative strategies are required to manage PLA waste more effectively and to preserve the intrinsic value of the original feedstock [22, 23, 24].

In this context, chemical recycling has emerged as a promising complementary approach, enabling the conversion of PLA waste into value‐added chemicals or its original monomers [25, 26, 27, 28, 29, 30]. In particular, alcoholysis converts PLA into lactate esters [31], which are widely used as green solvents or as intermediates for lactide regeneration, thereby supporting a circular model for PLA [32]. The efficiency of this process strongly depends on the catalytic system employed, with organic [33, 34], ionic, and metal‐based catalysts [30, 35, 36], especially zinc complexes, showing high activity and selectivity [37, 38, 39, 40].

In this regard, we recently reported phenoxy‐imine zinc complexes that efficiently catalyze both the polymerization of lactide and the chemical recycling of PLA and other polyesters [41, 42], including polyethylene terephthalate (PET) [43, 44, 45] and polycarbonates [46]. We also observed that microwave irradiation effectively promotes both alcoholysis [47] and hydrolysis of PLA [48]. The beneficial effects of microwave heating significantly reduced reaction times compared to conventional methods as well as the ability to operate under solvent‐free conditions with high selectivity and reproducibility [45].

Moreover, we have recently developed a one‐pot strategy for the degradation and functionalization of PLA using titanium isopropoxide as an inexpensive, commercially available, and nontoxic catalyst, enabling the formation of PLA‐based oligomers bearing unsaturated functionalities, which were subsequently employed in the preparation of thermoset materials [49].

In this contribution, we extend this approach to the chemical degradation of PLA waste using a range of different diols, such as ethylene glycol (EG), 1,4‐butanediol (1,4 BDO), *cis*‐2‐butene‐1,4‐diol (*cis*BD), and 3‐allyloxy‐1,2‐propanediol (APD), enabling access to a variety of novel functionalized lactyl‐based diols. A comparative study has been carried out between the phenoxy–imine pyridine zinc catalyst and titanium isopropoxide. Different reaction conditions have been investigated, highlighting how parameters such as the choice of diol and the control of experimental variables play a crucial role in determining the nature of the degradation products and their potential for further chemical transformation. Moreover, the bifunctional monomers obtained from the alcoholysis reactions were subsequently employed in polycondensation reactions with adipic acid, performed either stepwise or in a one‐pot procedure by direct addition of the carboxylic acid to the depolymerization mixture without isolating the intermediate monomers. Adipic acid is the most commercially important dicarboxylic acid primarily used for nylon production. Currently, adipic acid is predominantly produced from petrochemical feedstocks; however, sustainable bio‐based production routes are being actively developed as promising alternatives [50, 51]. About the diols, 1,4‐butanediol [52], 3‐allyloxy‐1,2‐propanediol [53], and ethylene glycol [54] can be also obtained from biomass.

The thermal behavior of the resulting polymers was then compared with those of the related copolymers obtained from the direct polycondensation of adipic acid with the initial diols.

Similar studies were recently reported for the chemical recycling and upcycling of polyethylene terephthalate (PET), promoted by a tetranuclear alkoxide titanium catalyst. The catalyst exhibited remarkable activity in PET methanolysis, enabling the recovery of polymerizable monomers and their subsequent repolymerization into high‐molecular‐weight polyesters [55].

## 2 Results and Discussion

### 2.1 Depolymerization Studies of Polylactic Acid Waste

For the chemical depolymerization studies, a commercial poly(lactic acid) waste sample (cups, *M*_n_ = 51,000 g/mol, *Đ* = 1.9) was selected.

Initially, ethylene glycol (EG) was chosen as the benchmark diol as it offers a simple and well‐established model for evaluating the depolymerization behavior of PLA under controlled conditions (Scheme 1).

**SCHEME 1:**
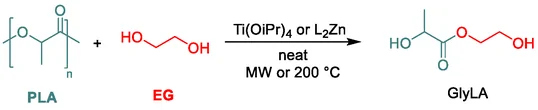
Alcoholysis reaction of PLA with ethylene glycol (EG).

Previous studies performed with the homoleptic phenoxy–imine pyridine zinc complex (Scheme 2 and Figure S1) showed that microwave irradiation significantly enhanced performance, enabling complete depolymerization in shorter reaction times in comparison to conventional heating [47, 48]. These findings prompt the question of whether the improvement observed under microwave conditions is intrinsic to the zinc catalyst or reflects a more general acceleration effect applicable to other catalytic systems.

**SCHEME 2:**
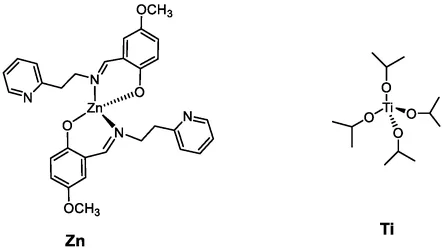
Structures of the zinc and titanium‐based catalysts used in this work.

To investigate this point, titanium (IV) isopropoxide (Ti(O^i^Pr)_4_) was selected as a comparative catalyst. This compound is inexpensive, commercially available, and operationally straightforward, making it a suitable reference system to explore the combined influence of catalyst identity and heating method on PLA depolymerization. At the same time, Ti(O^i^Pr)_4_ is traditionally used as polycondensation catalyst; thus, it could offer the opportunity to be used as single catalyst in sequential processes in which the PLA waste is depolymerized to produce new diols that could be directly condensed in a second step with dicarboxylic acids. The choice of this catalyst may therefore open the possibility to adopt a closed‐loop approach for the PLA upcycling.

Examining both catalysts (Scheme 2) under identical conventional and microwave conditions enables a systematic comparison, allowing catalyst‐specific effects to be distinguished from those arising solely from the heating technique.

The degradation reactions were performed at 200 °C on a commercial PLA sample in the absence of solvent by using three equivalents of unpurified ethylene glycol for each ester functionality of the polymer. The data regarding the depolymerization reactions of PLA by the selected zinc‐ and titanium‐based catalysts (Scheme 2) are summarized in Table 1.

**TABLE 1:** Alcoholysis of PLA by ethylene glycol (EG).^a^

Entry	[PLA]:[ROH]^b^	Cat	Heat	Time, min	*X*_Int_ c, %	*S*_RLa_ c, d, %	*Y*_RLa_ c, d, %
1	1:3	Zn	Thermal	60	97	89	86
2	1:3	Zn	MW	20	100	100	100
3	1:3	Ti	Thermal	60	95	87	82
4	1:3	Ti	MW	60	97	87	85
5	1:1	Ti	MW	75	89	nd	nd

a Reactions were carried out using a catalyst load of 0.66 mol % relative to lactyl unit, *T* = 200 °C.

b Calculations for the amounts of PLA and diol added to the system in the feed were made considering the single repeating unit of PLA with a molecular weight of 72.06 g/mol, the molecular weight of ethylene glycol of 62.07 g/mol.

c Determined by ^1^H NMR spectroscopy.

d The yield (*Y*_RLa_) of the target product is determined by accounting for all species detected after the reaction, while selectivity (*S*_RLa_) refers specifically to the alkyl mono‐substituted compound respect to oligomeric PLA fragments.

The homoleptic zinc catalyst provided high conversion of PLA under conventional heating, reaching almost complete conversion and selectivity after 1 h (entry 1, Table 1). Under microwave irradiation, after only 20 min (entry 2, Table 1), the complete conversion of PLA and the perfect selectivity in the production of glycolyl lactate were achieved, confirming that microwave activation significantly improves the efficiency of this system (Figure S2).

Titanium isopropoxide showed performance comparable to the zinc complex under conventional heating (entry 3, Table 1); differently, no meaningful improvement in conversion or selectivity was observed when switching from thermal to microwave heating (entry 4, Table 1). As expected, reducing the amount of EG, the reaction proceeded slower (entry 5, Table 1).

Experiments conducted with EG revealed clear differences between the two catalytic systems when the two activation modes were adopted. Overall, this comparison highlights a divergent behavior between the two systems: while microwave activation improved the conversion and selectivity obtained with the zinc catalyst, the titanium one appeared largely insensitive to the activation method, reinforcing the need to consider catalyst‐dependent mechanisms rather than general assumptions about microwave‐assisted depolymerization.

Although microwave heating did not offer any advantage in terms of PLA conversion or selectivity when the titanium catalyst was used, LCA studies demonstrated that microwave‐assisted PET glycolysis could substantially reduce the global warming potential of the bis(2‐hydroxyethyl) terephthalate (BHET) production [56]. Thus, the subsequent experiments were prevailingly performed with microwave assistance.

Subsequently, 1,4‐butanediol (1,4‐BDO) and different unsaturated diols such as *cis*‐2‐butene‐1,4‐diol (*cis*BD) and 3‐allyloxy‐1,2‐propanediol (APD) were selected. The rationale for selecting these diols is their ability to introduce unsaturation into the alcoholysis products, thereby enabling the formation of novel functionalized lactyl‐based diols. This approach allows the incorporation of unsaturated groups at different positions within the repeating unit, either along the polymer backbone or as pendant functionalities (Scheme 3) [53].

**SCHEME 3:**
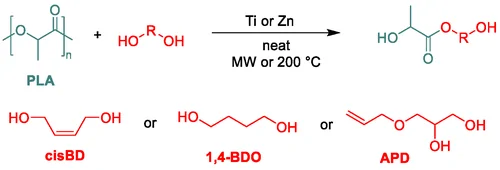
Alcoholysis reaction of PLA with different diols.

The depolymerization reactions were carried out under the same conditions used for glycolysis; data regarding the alcoholysis of PLA by 1,4‐BDO, *cis*BD, and APD are summarized in Table 2. Employing a 1:3 molar ratio of lactic acid repeating unit to diol, in the presence of the zinc catalyst and under microwave heating, *cis*BD afforded complete PLA conversion after 2 min (entry 2, Table 2), whereas complete conversion with EG was achieved after 20 min under the corresponding conditions (entry 2, Table 1). When the amount of *cis*BD was reduced to one equivalent per PLA repeating unit, 80% PLA conversion was obtained after 60 min (entry 3, Table 2), consistent with the trend observed with ethylene glycol.

**TABLE 2:** Alcoholysis of PLA with *cis*BD, 1,4‐BDO, and APD.^a^

Entry	[PLA]:[R(OH)_2_]^b^	Cat	R(OH)_2_	Heating	Time, min	*X*_Int_ c, %
1	1:3	Zn	*cis*BD	Thermal	5	100
2	1:3	Zn	*cis*BD	MW	2	100
3	1:1	Zn	*cis*BD	MW	60	80
4	1:3	Ti	*cis*BD	Thermal	60	91
5	1:3	Ti	*cis*BD	MW	60	93
6	3:1	Ti	*cis*BD	Thermal	60	35
7	3:1	Ti	*cis*BD	MW	60	33
8^d^	3:1	Ti	*cis*BD	Thermal	48h	—
9^d^	3:1	Ti	*cis*BD	MW	60	11
10	1:3	Ti	1,4‐BDO	MW	60	96
11	1:3	Zn	APD	MW	1	100
12	1:3	Ti	APD	MW	10	61
13	1:3	Ti	APD	MW	30	89
14	1:3	Ti	APD	MW	60	91
15	1:10	Ti	APD	MW	60	100
16	3:1	Ti	APD	MW	30	29
17	3:1	Ti	APD	MW	60	40

^a^Reactions were carried out using a catalyst loading of 0.66 mol% relative to lactyl unit and under neat conditions at 200 °C using conventional heating or microwave.

b Calculations for the amounts of PLA and diol added to the system (feed) were made considering the single repeating unit of PLA with a molecular weight of 72.06 g/mol; *cis*BD of 88.11 g/mol; 1,4‐BDO = 90.12 g/mol; APD of 132.16 g/mol.

c Determined by ^1^H NMR spectroscopy.

d Reaction temperature = 170 °C.

Titanium isopropoxide proved to be less efficient than the zinc complex: 90% PLA conversion was reached after 1 h, with comparable performance under both conventional and microwave heating (entries 4 and 5, Table 2), in agreement with the behavior previously observed for glycolysis.

As expected, when the amount of *cis*BD was reduced to 1/3 of the lactic acid repeating units (entries 6 and 7, Table 2), a marked decrease in the alcoholysis rate was observed. Under conventional heating, a polymer conversion of 35% occurred after 1 h (entry 6, Table 2). In contrast, microwave irradiation led to 33% conversion. This difference was more evident when the reactions were performed at lower temperature (170 °C); under these conditions, when conventional heating was used, no conversion of polymer was achieved within 48 h; differently, a conversion of 11% was observed after 1h when microwave irradiation was used (entries 8 and 9, Table 2).

This pronounced difference can be rationalized by considering that the experiments were performed under solvent‐free conditions. Under conventional heating, the process is limited by the poor diffusion of the diol within the polymeric matrix. In contrast, microwave irradiation promotes the formation of localized “hot spots,” which may enhance polymer alcoholysis at a local level by facilitating more efficient energy transfer and reactant mobility.

When the amount of diol is reduced, the advantage of microwave heating becomes even more evident as localized heating effects play a more critical role in promoting the reaction. No difference in terms of activity was observed when 1,4 butanediol (1,4 BDO) was used instead of *cis*BD, suggesting that the increased rigidity of the monomer, arising from the internal double bond, did not significantly affect the reaction (entry 10, Table 2).

Next, we investigated a diol featuring an external unsaturation, namely, 3‐allyloxy‐1,2‐propanediol (APD). We anticipated that APD would outperform *cis*‐2‐butene‐1,4‐diol (*cis*BD) as its hydroxyl groups are oriented on the same side of the molecule, potentially enhancing its polarity. Consistent with this expectation, microwave heating afforded a quantitative conversion within only 1 min (entry 11, Table 2), supporting the high reactivity of APD.

The same effect was observed also with titanium catalyst (entries 12–14, Table 2).

Experimentally determined dielectric constants for both APD and *cis*BD are not available in standard databases, making a direct comparison difficult. As an alternative descriptor, the topological polar surface area (TPSA) can be used to estimate polarity and hydrogen‐bonding capability. APD exhibits a TPSA of approximately 49.7 Å, whereas *cis*BD shows a lower value of about 40.5 Å. Given the correlation between TPSA, molecular polarity, and intermolecular interactions, these data suggest that APD possesses higher polarity and may display enhanced reactivity in polar reaction pathways compared to *cis*BD.

Subsequent experiments were conducted by varying the amount of diol. When 10 equivalents of APD were employed (entry 15, Table 2), quantitative PLA conversion was achieved after 60 min, selectively affording APD‐lactate (APD‐La) (Figures 1 and S7–S10). When the APD amount was reduced to one‐third of the PLA repeating units, PLA conversions of 29% and 40% were obtained after 30 and 60 min, respectively (entries 16 and 17, Table 2). The structure of APDLa was confirmed by ^1^H, ^13^C, COSY, and HSQC NMR analyses, and a detailed ^1^H NMR assignment is provided in Table S1 of the Supporting Information.

**FIGURE 1:**
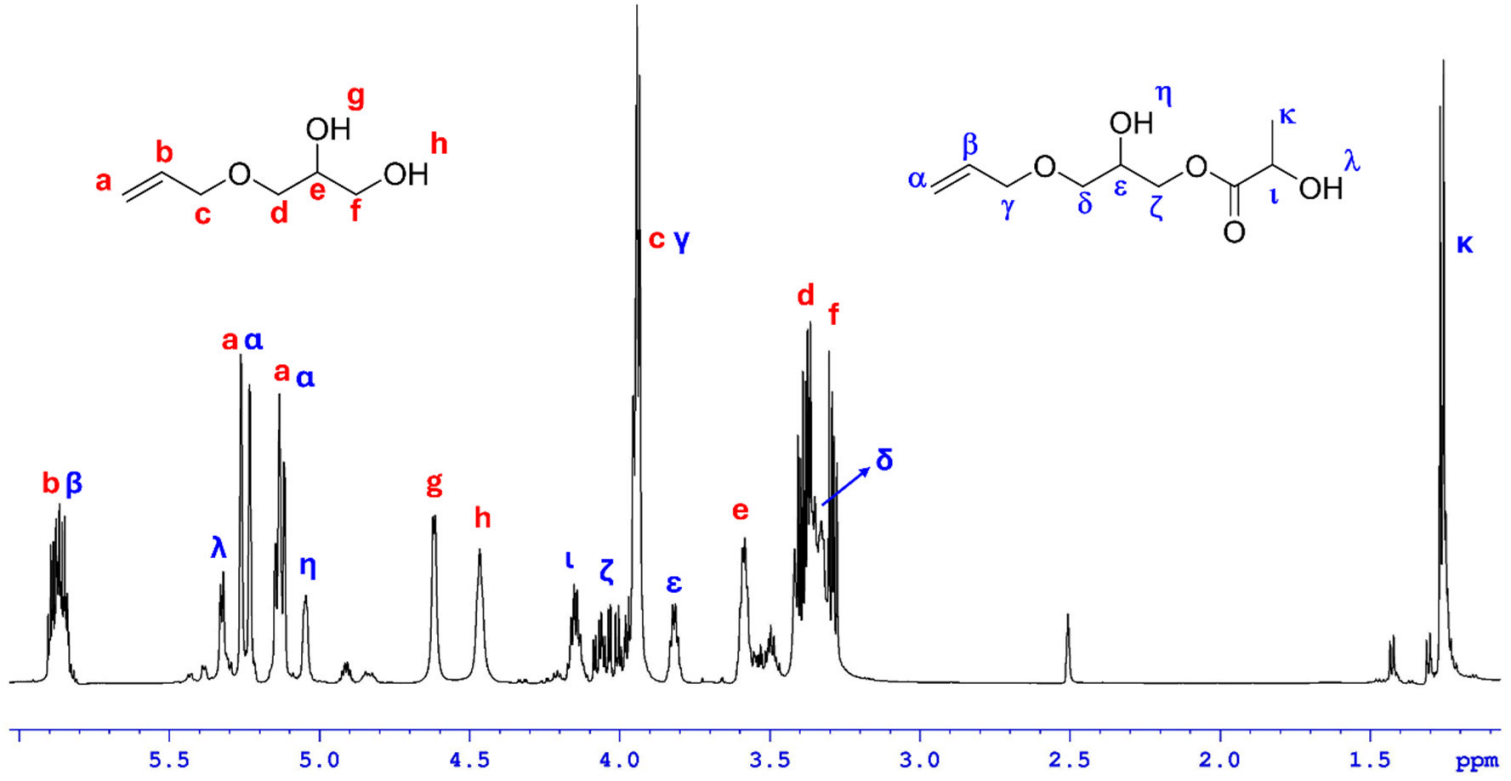
^1^H NMR spectrum (DMSO, 600 MHz, 298 K) of the product of PLA alcoholysis using APD (entry 15, Table 2).

The products obtained from the depolymerization reaction of PLA waste with diverse diols represent interesting building blocks for the synthesis of novel aliphatic polyesters with original microstructures.

### 2.2 Melt‐Polycondensation of the Lactyl‐Based Diols With Adipic Acid

We demonstrated that when PLA depolymerization was carried out in the presence of a slight excess of diol, PLA could be quantitatively converted into bifunctional monomers. The so obtained diols, having embedded the lactic acid repeating, could be exploited for the preparation of novel polyesters by simple polycondensation reactions with bifunctional carboxylic acids. Here, we have explored the use of these new diols, obtained from the alcoholysis of PLA waste, in polycondensation reactions with adipic acid (AA).

As already pointed out, the selected titanium isopropoxide is traditionally used also in polycondensation reaction of diols with dicarboxylic acids. To prove the possibility to achieve a novel polyester by using the PLA glycolysis products, a polycondensation run was carried out by using the mixture of monomers obtained by glycolysis reaction of PLA (glycolyl lactate and the residual ethylene glycol, as summarized in entry 4 of Table 1), without further purification, with adipic acid (molar ratio 3:1). For this polycondensation experiment, Ti(O^i^Pr)_4_ was used at 10 mol% relative to the initial moles of PLA repeating units used to generate the glycolysis mixture at 200 °C (Scheme 4).

**SCHEME 4:**
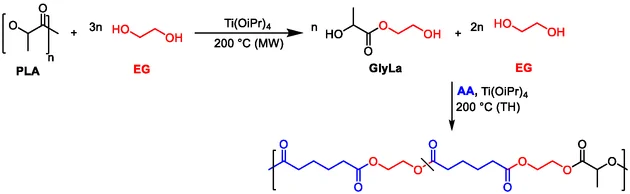
Polycondensation reaction between adipic acid (AA) and the mixture of ethylene glycol (EG) and glycolyl lactate (GlyLa).

After 60 min, the viscosity of the solution increased notably; after this time, the flask was connected to the vacuum system for an additional 2 h. The resulting product was dissolved in a minimal amount of chloroform and precipitated into methanol. The polymer, recovered by filtration, was dried overnight in an oven.

Subsequently, a further polycondensation reaction was carried using a one‐pot, two‐step procedure. In this approach, PLA was first depolymerized via glycolysis by thermal heating, and then adipic acid was directly added to the resulting reaction mixture without isolation of the product mixture and without adding additional catalyst. As above, after 2 h, the viscosity of the solution increased; the mixture was kept under vacuum for an additional 2 h.The product was dissolved in chloroform, precipitated into methanol, filtered, and dried oven‐night.

Both polyesters were characterized by ^1^H NMR spectroscopy and thermal analysis and were compared with the poly(ethylene adipate) (PEA), obtained from polycondensation reaction between ethylene glycol and adipic acid, performed under identical reaction conditions (Figures 2 and S11–S21).

**FIGURE 2:**
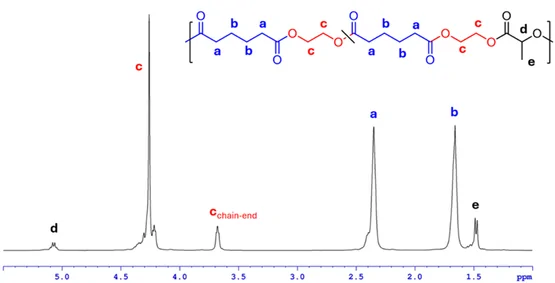
^1^H NMR spectrum (CDCl_3_, 600 MHz, 298 K) of poly(ethylene adipate‐*co*‐lactate), (PEALa).

The ^1^H NMR spectrum of poly(ethylene adipate‐*co*‐lactate) (PEALa) displayed, in addition to the characteristic signals of PEA, two additional resonances: a quartet at 5.07 ppm and a doublet at 1.48 ppm, assigned to the methine and methyl protons of the lactyl units incorporated into the polymer chain, respectively (Figure 2). These results demonstrated that glycolyl lactate is successfully incorporated into the poly(ethylene adipate) polymer backbone. Moreover, integration of the proton signals indicated that the ethylene glycol/glycolyl lactate/adipic acid ratio in the final polymer closely matched the initial composition employed in the depolymerization/polycondensation reaction mixture.

Further evidence for the successful incorporation of the lactyl unit into the polymer backbone was provided by 2D DOSY NMR spectra obtained at 298 K in CDCl_3_ solution (Figure 3). Nicely, all signals relative to the ethylene adipate and lactate units have the same diffusion coefficient, thus indicating that they belong to the same macromolecular chain.

**FIGURE 3:**
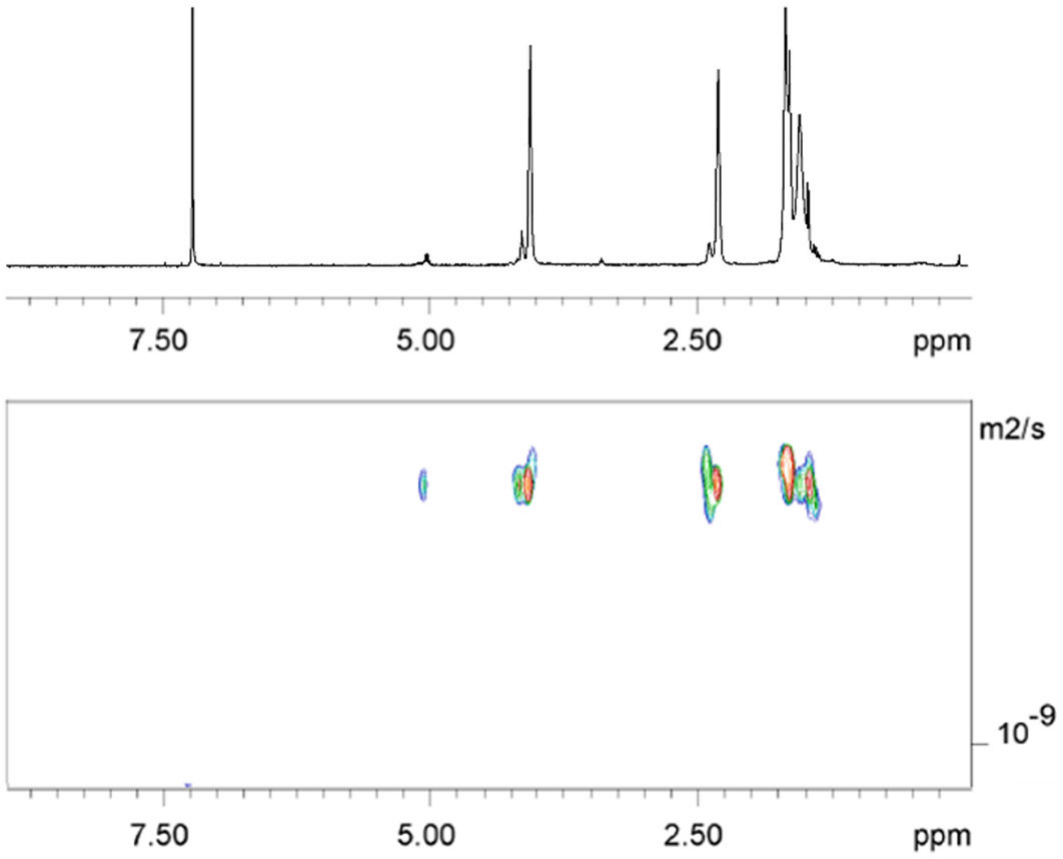
2D DOSY NMR spectrum (600 MHz, CDCl_3_, 298 K) of poly(ethylene adipate‐*co*‐lactate), PEALa.

The PEA and PEALa were characterized by differential scanning calorimetry (DSC) and thermogravimetric analysis (TGA). DSC analysis (Figures S14 and S20) revealed two distinct thermal behaviors: poly(ethylene adipate) exhibited a semicrystalline nature, characterized by a glass‐transition temperature (*T*_g_) of −45 °C, together with crystallization and melting transitions at −7 °C (*T*_c_) and 45 °C (*T*_m_), respectively. In contrast, the poly(ethylene adipate‐*co*‐lactate), in which lactyl units have been incorporated into the polymer backbone, showed a loss of crystallinity, resulting in the formation of an amorphous material with a *T*_g_ of −42 °C.

TGA was carried out to determine the thermal stability of the samples. Comparison of the TGA curves of PEA and PEALa (Figures S15 and S21 and Table 3) showed that the presence of lactyl units in the copolymer leads to a slight decrease in the temperatures at 5% mass loss (*T*_5%_), which goes from 262 °C for PEA to 254 °C for PEALa. Both the PEA and PEALa degrade in a single step with inflection point on the TG curve at 349 and 377, respectively. This behavior may reflect the higher thermal stability of PEALa with respect to the one of PEA, indicating that the inclusion of lactyl units into the PEA chains strongly delay the thermal degradation of the PEALa copolymer. The different microstructure of PEALa may modify the local chemical environment of the ester groups and affect the thermal chain‐scission processes, accounting for the higher degradation temperature observed for PEALa compared with PEA. The single‐step degradation observed for PEALa is consistent with a random distribution of the lactyl units along the poly(ethylene adipate) chains. In contrast, a different poly(ethylene adipate‐*co*‐L‐lactic acid) copolymers containing blocky LA and EA sequences displayed two degradation events [57].

**TABLE 3:** TGA results of the samples analyzed.

Sample	T_5%_, °C	T_50%_, °C	T_d1_, °C	T_d2_, °C
*Std. Dev.*	±1 °C	±1 °C	±1 °C	±1 °C
PEA	262	342	349	—
PEALa	254	365	377	—
PBA	184	350	366	—
PBALa	261	366	374	—
P*c*BA	273	375	348	424
P*c*BALa	261	361	337	422

Subsequently, the same approach was extended to the alcoholysis products of PLA obtained with the other diols. The polycondensation of the products obtained from the alcoholysis of PLA in the presence of 1,4‐butanediol (1,4‐BDO) with adipic acid was carried out. The reaction was performed with a one‐pot two‐step procedure, by using Ti(O^i^Pr)_4_ as catalyst (10 mol% relative to the PLA repeating unit) and an excess of 1,4‐butanediol (3:1 diol‐to‐repeating‐unit molar ratio). The alcoholysis reaction was conducted at 180 °C under continuous stirring for approximately 60 min, leading to complete conversion of the PLA into butylene lactate. Following the addition of adipic acid, a progressive increase in the viscosity of the reaction mixture was observed over time, similarly to the behavior described in the previous experiment. For comparison, a poly(butylene adipate) (PBA) sample was prepared in analogous conditions.

The obtained poly(ethylene adipate‐*co*‐lactate) (PBALa) was characterized by ^1^H NMR and 2D DOSY spectroscopy (Figures S22, S26, and S27), that confirmed the incorporation of lactyl units into the poly(butylene adipate) polymer chain. The composition of the final polymer fully reflected the initial reaction mixture ratio, confirming the efficient incorporation of both monomers (Figures S22 and S26). Thermal analysis showed a semicrystalline nature of poly(butylene adipate) (PBA), as evidenced by a crystallization peak at 23 °C and a melting peak at 40 °C (Figure S24). Likewise, poly(butylene adipate‐*co*‐lactate) (PBALa) exhibited semicrystalline behavior, characterized by a crystallization peak at −2 °C and two distinct melting peaks at 17 and 26 °C (Figure S30). The presence of multiple melting transitions suggests the formation of crystalline domains with different sizes and/or degrees of structural regularity. Notably, the incorporation of lactyl units into the polymer chain did not suppress crystallization, indicating that the semicrystalline character of PBA was preserved in the copolymer.

Comparison of the TGA curves for the PBALa with that one of PBA revealed that the incorporation of lactyl units into the polymer backbone resulted in an increased thermal stability of PBALa respect to the PBA (Figures S25 and S31 and Table 3). Notably, while the PBA showed a two‐step degradation, for the PBALa, a single degradation step was observed.

The same polycondensation reaction was successfully carried out with the products of alcoholysis of PLA with *cis*‐2‐butene‐1,4‐diol (*cis*BD), again by using a one‐pot two‐step procedure. Compared with saturated analogs, *cis*BD offers a significant synthetic advantage: upon alcoholysis of PLA, it generates novel diols that retain the carbon–carbon double bond. These unsaturated monomers are subsequently incorporated into the polymer during polycondensation, introducing reactive sites along the polymer backbone that could be exploited for postpolymerization functionalization. The obtained poly(*cis*‐butene adipate‐*co*‐lactate), P*c*BALa, was fully characterized by ^1^H and ^13^C NMR analysis, as well as by dimensional NMR analyses such as DOSY and HSQC (Figures S36–S39).

DSC analysis reveals for P*c*BA a semicrystalline nature, characterized by a glass‐transition temperature (*T*_g_) of −54 °C, together with crystallization and melting transitions at −6 °C (*T*_c_) and 48 °C (*T*_m_), respectively (Figure S34). In contrast, the incorporation of lactyl units into the polymer backbone suppresses crystallinity, resulting in the formation of an amorphous material with a *T*_g_ value of −32 °C (Figure S40).

The TGA curves of poly(*cis*‐butene adipate) (P*c*BA) and poly(*cis*‐butene adipate‐*co*‐lactate) (P*c*BALa) indicated that the incorporation of lactyl units slightly affect the thermal degradation profile, with PcBA exhibiting slightly higher degradation temperatures; in both the copolymers, the DTG thermograms indicate two degradation stages either for P*c*BA or P*c*BALa (Figures S35 and S41 and Table 3).

## 3 Conclusions

Upcycling of polymeric materials is a crucial strategy to transform plastic waste into higher‐value materials, driving a circular economy by conserving resources and reducing environmental impact.

This study demonstrates an efficient strategy for the chemical upcycling of PLA waste into novel value‐added functionalized diols and their subsequent transformation into new polyester materials. The comparative analysis of catalytic systems revealed that the phenoxy–imine pyridine zinc complex exhibits superior performance under microwave irradiation, enabling rapid and selective depolymerization, whereas titanium isopropoxide shows comparable activity under conventional conditions but remains largely insensitive to microwave irradiation.

The nature of the diol was found to play a crucial role in determining both PLA conversion under the investigated conditions and the structure of the resulting products. The influence of diol structure, polarity, and stoichiometry highlights the importance of reaction design in controlling efficiency and selectivity.

The use of unsaturated diols allowed the direct introduction of functional groups into the recovered monomers, providing versatile building blocks for further chemical transformations. Among the different possible approaches, in our current work, we have presented a method for using these new compounds as building blocks in polycondensation reaction with adipic acid, a cheap and key precursor for polyester and polyamide synthesis. Importantly, the feasibility of integrating depolymerization and repolymerization steps in a one‐pot, two‐step process using the titanium isopropoxide as the catalytic system was demonstrated, avoiding intermediate purification and simplifying the overall workflow. The resulting polyesters exhibited distinct structural features compared with those derived from the related diols, underscoring the potential of this approach to access novel materials from waste resources.

Overall, this work provides a proof of concept for a circular and sustainable approach to PLA valorization, in which polymeric waste is converted into functional monomers and subsequently into new materials with tailored properties. The combination of efficient catalytic systems, energy‐conscious methodologies, and flexible synthetic design offers promising perspectives for the development of advanced chemical recycling strategies for other polyesters.

## 4 Experimental Section

### 4.1 Materials and Methods

All operations involving air‐ and/or moisture‐sensitive compounds were performed under an inert nitrogen atmosphere, either inside an MBraun Labmaster glove box or using standard Schlenk line techniques. Prior to use, all glassware and reaction vials employed in alcoholysis experiments were dried overnight at 120 °C and subsequently subjected to three vacuum/nitrogen purge cycles.

### 4.2 Reagents and Solvents

Commercial PLLA waste cups (Natura Bio, provided by Aristea or Selex) were employed without further purification. Representative PLLA sample was characterized by gel permeation chromatography (GPC) to determine the molecular mass (*M*_*n*_ = 51 KDa, *Ð* = 1.9) and TGA to verify the presence of impurities. TGA established that the residual after thermal decomposition up to 340 °C was around 5%. Before use, the cups were cut into small pieces of approximately 1 cm^2^. Ethylene glycol (EG), 1,4‐butanediol (1,4 BDO), *cis*‐2‐butene‐1,4‐diol (*cis*BD), 3‐allyloxy‐1,2‐propanediol (APD), and titanium isopropoxide were purchased from Merck or Carlo Erba and used as received.

### 4.3 Instruments and Measurements

#### 4.3.1 NMR Spectroscopy

^1^H NMR and ^13^C NMR spectra were registered at 298 K using Bruker Avance instruments operating at 300, 400, and 600 MHz. Chemical shifts (*δ*) are reported in ppm, while coupling constants (*J*) are given in hertz. ^1^H spectra were internally referenced to the residual solvent peaks at *δ* = 7.16 (C_6_D_6_), *δ* = 7.26 (CDCl_3_), and *δ* = 2.50 (DMSO). ^13^C spectra were referenced to *δ* = 128.06 (C_6_D_6_), *δ* = 77.16 (CDCl_3_), and *δ* = 39.52 (DMSO).

#### 4.3.2 Microwave Reactor

All the microwave‐assisted reactions have been per formed using the CEM reactor model Discover 2.0. The reactor can work with 10 and 35 mL glass‐pressurized vessels and is equipped with an IR temperature sensor and a touchscreen interface from which it is possible to program the parameters of the reaction, such as temperature, time, stirring power, power limit that the instrument can reach, and max pressure that the reactor can handle before activating the safety control of pressure and release all the excess gasses.

#### 4.3.3 DSC Analysis

Glass transition temperatures (*T*_g_), melting points (*T*_m_), and enthalpy of fusion (Δ*H*_m_) of the polymer samples were measured by DSC using aluminum pans and a DSC 2920 TA Instruments apparatus, calibrated with indium. Measurements were performed under nitrogen flow with a heating rate of 10 °C min^−1^ in the range opportune range of temperature. DSC data were processed with TA Universal Analysis v2.3.

#### 4.3.4 TGA Analysis

TGA measurements were performed on a Netzsch TG 209 F1 analyzer in the temperature range of 40–800 °C at a heating rate of 10 °C min^−1^ under nitrogen flow.

### 4.4 Alcoholysis Reaction of PLLA by Diol With Microwave

PLLA plastic cups, the catalyst, and the diol were added to 10 mL MW vessel with a magnetic stirrer. The reaction was conducted at the required temperature under magnetic stirring for the designated time. The conversion of PLLA and the percentages of alkyl lactate and oligomers were calculated via ^1^H NMR spectroscopy using the following equations

$$X_{\mathrm{Int}}\left(\%\right)=1\text{−}\;\frac{\left[\mathrm{Int}\right]}{{\left[\mathrm{Int}\right]}_{0}}\;\times \;100$$

$$S_{A\text{−}\mathrm{La}}\left(\%\right)=\frac{\left[A-\mathrm{La}\right]}{\left[\mathrm{Int}\right]-{\left[\mathrm{Int}\right]}_{0}}\times 100$$

$$Y_{A\text{−}\mathrm{La}}\left(\%\right)=\;X_{\mathrm{Int}}\;\times \;S_{A\text{−}\mathrm{La}}$$

### 4.5 Alcoholysis Reaction of PLLA by Diol With Conventional Heating

PLLA plastic cups, the catalyst, and the diol were added to 10 mL Schlenk tube with a magnetic stirrer. The reaction was conducted at the required temperature under magnetic stirring for the designated time. The conversion of PLLA and the percentages of alkyl lactate and oligomers were calculated via ^1^H NMR spectroscopy, as described in the previous paragraph.

### 4.6 Polycondensations of the Lactyl‐Based Diols With Adipic Acid

A 50 mL Schlenk flask, equipped with a magnetic stir bar, was connected to a vacuum line and subjected to three vacuum–nitrogen cycles (2 min each). Subsequently, 3.47 × 10^−3^ mol of waste PLA, 10.4 × 10^−3^ mol of diol, and 3.47 × 10^−4^ mol of catalyst (10 mol% relative to the PLA repeating units) were added. After stirring under nitrogen for 60 min at 200 °C, 3.47 × 10^−3^ mol of adipic acid was introduced, corresponding to a 1:3 molar ratio with respect to the hydroxyl groups.

After 60 min, the system was connected to a vacuum trap. After 2 h, the product obtained was dissolved in a minimum amount of chloroform and precipitated dropwise into ~40 mL of cold methanol. The polymer was collected by filtration and dried.

## Conflicts of Interest

The authors declare no conflicts of interest.

## Supporting information

Supplementary Material

## Data Availability

The data that support the findings of this study are available in the Supporting Information of this article.
