# Supplementary material for: Chemical Upcycling of Poly(Lactic Acid) Waste Into Lactyl‐Based Diols for the Synthesis of New Polyesters
Source: Chempluschem. 2026 Oct 11;91(10):e70261. doi: 10.1002/cplu.70261 (PMC13654598; doi:10.1002/cplu.70261)
Supplement: Supplementary file 1 [file CPLU-91-e70261-s001.pdf]

Supporting Information

Chemical Upcycling of Poly(lactic acid) Waste into Value-Added Functionalized Monomers for the Synthesis of New Polyesters.

Federica Santulli,[1] Andrea Annunziata,[2] Pasquale D'Angelo,[1] Marina Lamberti,[1] Leonardo Marchese,[3] Daniela Pappalardo,*[2] Mina Mazzeo.*[1]

[1]*Department of Chemistry and Biology "Adolfo Zambelli", Università degli Studi di Salerno, via Giovanni Paolo II, 132, 84084, Fisciano, Italy*

[2]*Department of Science e Technology, Università degli Studi del Sannio, via de Sanctis snc, 82100, Benevento, Italy*

[3]*Department of Science and Technological Innovation, Università del Piemonte Orientale, Viale T. Michel 11, Alessandria 15121, Italy*

Email: pappalardo@unisannio.it; mmazzeo@unisa.it

Table of Contents

Synthesis and characterization of the Zinc complex 5

Figure S1. ^{1}H NMR spectrum (600 MHz, C_6D_6, 298 K) of **Zn** complex. 5

Characterization of degradation products 6

- Alcoholysis by ethylene glycol (EG) 6

Figure S2. ^{1}H NMR spectrum (400 MHz, $CDCl_3$, 298 K) of glycolyl lactate (GlyLa). 6

Figure S3. ^{13}C NMR spectrum (150 MHz, DMSO, 298 K) of glycolyl lactate. 6

- Alcoholysis by 1,4-butanediol (1,4-BDO) 7

Figure S4. ^{1}H NMR spectrum (400 MHz, $CDCl_3$, 298 K) of 4-hydroxybutyl lactate (BDLa). 7

Figure S5. ^{13}C NMR spectrum (150 MHz, DMSO, 298 K) of 4-hydroxybutyl lactate (BDLa). 7

- Alcoholysis by *cis*-2-butene-1,4-diol (*cis*-BD) 8

Figure S6. ^{1}H NMR spectrum (400 MHz, $CDCl_3$, 298 K) of 4-hydroxy-*cis*-butyl lactate (*c*BDLa). . 8

- Alcoholysis by 3-allyloxy-1,2-propanediol (APD) 8

Figure S7. ^{1}H NMR spectrum (DMSO, 600 MHz, 298 K) of ADP-lactate (ADPLa). 8

Table S1. ^{1}H NMR assignment of APD-lactate (APDLa) 9

Figure S8. COSY NMR spectrum (600 MHz, DMSO, 298 K) of ADP-lactate (ADPLa). 9

Figure S9. HSQC NMR spectrum (600 MHz, DMSO, 298 K) of ADP-lactate (ADPLa). 10

Figure S10. ^{13}C NMR spectrum (150 MHz, DMSO, 298 K) of ADP-lactate (ADPLa). 10

Characterization of the polycondensation products 11

- *Ethylene glycol (EG)* 11

Scheme S1. Polycondensation reaction of the adipic acid (AA) and ethylene glycol (EG). 11

Figure S11. ^{1}H NMR spectrum (600 MHz, $CDCl_3$, 298 K) of poly(ethylene)adipate, PEA. 11

Figure S12. HSQC NMR spectrum (600 MHz, $CDCl_3$, 298 K) of poly(ethylene)adipate, PEA...... 12

Figure S13. ^{13}C NMR spectrum (150 MHz, $CDCl_3$, 298 K) of poly(ethylene)adipate, PEA. *TFA= trifluoroacetic acid.* 12

Figure S14. DSC of poly(ethylene)adipate, PEA. 13

Figure S15. TGA of poly(ethylene)adipate, PEA. 13

Scheme S2. Polycondensation reaction of the adipic acid (AA) with the mixture ethylene glycol (EG) and glycolyl lactate (GlyLa). 14

Figure S16. ^{1}H NMR spectrum (600 MHz, $CDCl_3$, 298 K) of poly(ethylene adipate-*co*-lactate), PEALa. 14

Figure S17. 2D DOSY NMR spectrum (600 MHz, $CDCl_3$, 298 K) of poly(ethylene adipate-*co*-lactate), PEALa. 15

Figure S18. HSQC NMR spectrum (600 MHz, $CDCl_3$, 298 K) of poly(ethylene adipate-*co*-lactate), PEALa. 15

Figure S19. ^{13}C NMR spectrum (150 MHz, $CDCl_3$, 298 K) of poly(ethylene adipate-*co*-lactate), PEALa. 16

Figure S20. DSC of poly(ethylene adipate-*co*-lactate), PEALa. 16

Figure S21. TGA of poly(ethylene adipate-*co*-lactate), PEALa. 17

- *1,4 butane diol (BD)* 18

Scheme S3. Polycondensation reaction of the adipic acid (AA) and 1,4 butane diol (BD)............. 18

Figure S22. ^{1}H NMR spectrum (600 MHz, $CDCl_3$, 298 K) of poly(butylene)adipate, PBA........... 18

Figure S23. ^{13}C NMR spectrum (150 MHz, $CDCl_3$, 298 K) of poly(butylene)adipate, PBA.......... 19

Figure S24. DSC of poly(butylene)adipate, PBA. 19

Figure S25. TGA of poly(butylene)adipate, PBA. 20

Scheme S4. Polycondensation reaction of the adipic acid (AA) with the mixture of 1,4 butane diol (BD) and 4-hydroxybutyl lactate (BDLa). 20

Figure S26. ^{1}H NMR spectrum (600 MHz, $CDCl_3$, 298 K) of poly(butylene adipate-*co*-lactate), PBALa. 21

Figure S27. 2D DOSY spectrum (600 MHz, $CDCl_3$, 298 K) of poly(butylene adipate-*co*-lactate), PBALa. 21

Figure S28. HSQC NMR spectrum (600 MHz, $CDCl_3$, 298 K) of poly(butylene adipate-*co*-lactate), PBALa. 22

Figure S29. ^{13}C NMR spectrum (150 MHz, $CDCl_3$, 298 K) of poly(butylene adipate-*co*-lactate), PBALa. 22

Figure S30. DSC of poly(butylene adipate-*co*-lactate), PBALa. 23

Figure S31. TGA of poly(butylene adipate-*co*-lactate), PBALa. 23

- *1,4-cis butene diol (cis-BD)* 24

Scheme S5. Polycondensation reaction of the adipic acid (AA) and 1,4-*cis* butene diol (*cis*-BD). . 24

Figure S32. ^{1}H NMR spectrum (600 MHz, $CDCl_3$, 298 K) of poly(*cis*-butene) adipate, P*c*BA. 24

Figure S33. ^{1}H NMR spectrum (150 MHz, $CDCl_3$, 298 K) of poly(*cis*-butene) adipate, P*c*BA. *TFA= trifluoroacetic acid.* 25

Figure S34. DSC of poly(*cis*-butene) adipate, P*c*BA.. 25

Figure S35. TGA of poly(*cis*-butene) adipate, P*c*BA. 26

Scheme S6. Polycondensation reaction of the adipic acid (AA) with the mixture 1,4-*cis* butene diol (*cis*-BD) and 4-hydroxy-*cis*-butyl lactate (*c*BDLa).. 26

Figure S36. ^{1}H NMR spectrum (600 MHz, $CDCl_3$, 298 K) of poly(*cis*-butene adipate-*co*-lactate), P*c*BALa. 27

Figure S37. 2D DOSY NMR spectrum (600 MHz, $CDCl_3$, 298 K) of poly(*cis*-butene adipate-*co*-lactate), P*c*BALa. 27

Figure S38. HSQC NMR spectrum (600 MHz, $CDCl_3$, 298 K) of poly(*cis*-butene adipate-*co*-lactate), P*c*BALa. 28

Figure S39. ^{13}C NMR spectrum (150 MHz, $CDCl_3$, 298 K) of poly(*cis*-butene adipate-*co*-lactate), P*c*BALa. *TFA= trifluoroacetic acid.* 28

Figure S40. DSC of poly(*cis*-butene adipate-*co*-lactate), P*c*BALa........ 29

Figure S41. TGA of poly(*cis*-butene adipate-*co*-lactate), P*c*BALa. 29

Synthesis and characterization of the Zinc complex

Synthesis of ligand LH

In a 50 mL round-bottom flask 2-(2-pyridyl)ethylamine (0.122 mg, 1 mmol), 2 hydroxy-5-methoxybenzaldehyde (0.152 mg, 1 mmol) and 25 mL of ethanol were introduced. The reaction medium was magnetically stirred at reflux temperature for the night. The solvent was eliminated by evaporation at reduced pressure, and the resulting solid was dried under vacuum. ^{1}H NMR (600 MHz, C_6D_6, 298 K): δ 13.04 (br, 1H, -OH), 8.46 (d, J =4.2 Hz, 1H, Ar), 7.65 (s, 1H, CH=N), 7.00 (m, 2H, Ar), 6.73 (m, 2H, Ar), 6.57 (t, J = 5.4 Hz, 1H, Ar), 6.45 (d, J = 3.0 Hz, 1H, Ar), 3.72 (t, J = 7.2 Hz, 2H, N–CH_2), 3.10 (s, 3H, O–CH_3), 2.89 (t, J = 7.2 Hz, 2H, N–CH_2–CH_2). ^{13}C NMR (150 MHz, C_6D_6, 298K): δ 165.01, 159.35, 155.74, 151.99, 149.43, 135.50, 123.25, 120.95, 118.89, 117.75, 115.05, 59.00, 55.03, 39.22.

Synthesis of zinc complex 1

The synthesis of the homoleptic zinc complex was carried out following the procedure reported in the literature.[1]

The synthesis was carried out under inert conditions in an MBraun Labmaster glovebox. In a 20 mL vial, the ligand LH (1.350 g, 5.27 mmol, 2 eq.) was weighed and was dissolved in 5 mL of dry benzene. In another vial, the metal precursor $ZnEt_2$ (0.333 g, 2.64 mmol, 1 eq.) was weighed and was dissolved in 2 mL of dry benzene. The $ZnEt_2$ solution added drop by drop to the ligand solution. Once the addition was complete, the reaction mixture was left to stir for 2 h at room temperature. The solvent was removed under vacuum and the obtained solid was washed three times with n-hexane. The formation of the desired species was confirmed by NMR analysis.

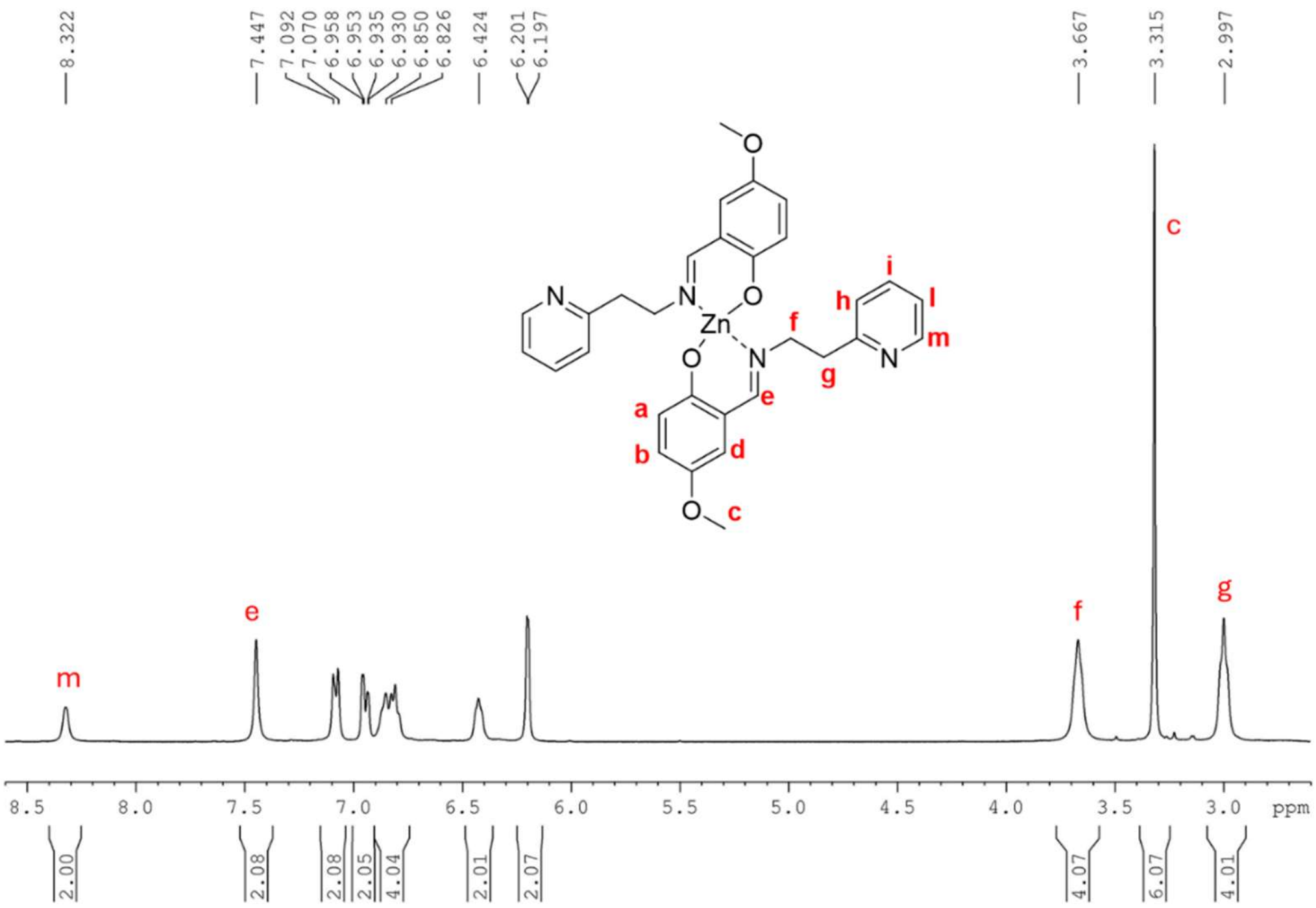

Figure S1. ^{1}H NMR spectrum (600 MHz, C_6D_6, 298 K) of **Zn** complex.

^{1}H NMR (600 MHz, C_6D_6, 298 K): δ 8.46 (br, 2H, Hm), 7.45 (s, 2H, He), 7.08 (d, J = 8.8 Hz, 2H, Ar), 6.95 (d, J = 7.2, 2H, Ar), 6.83 (m, 4H, Ar), 6.42 (br, 2H, Ar), 6.20 (s, 2H, Ar), 3.67 (br, 4H, Hf), 3.31 (s, 6H, Hc), 3.00 (br, 4H, Hg).

Characterization of degradation products

- Alcoholysis by ethylene glycol (EG)

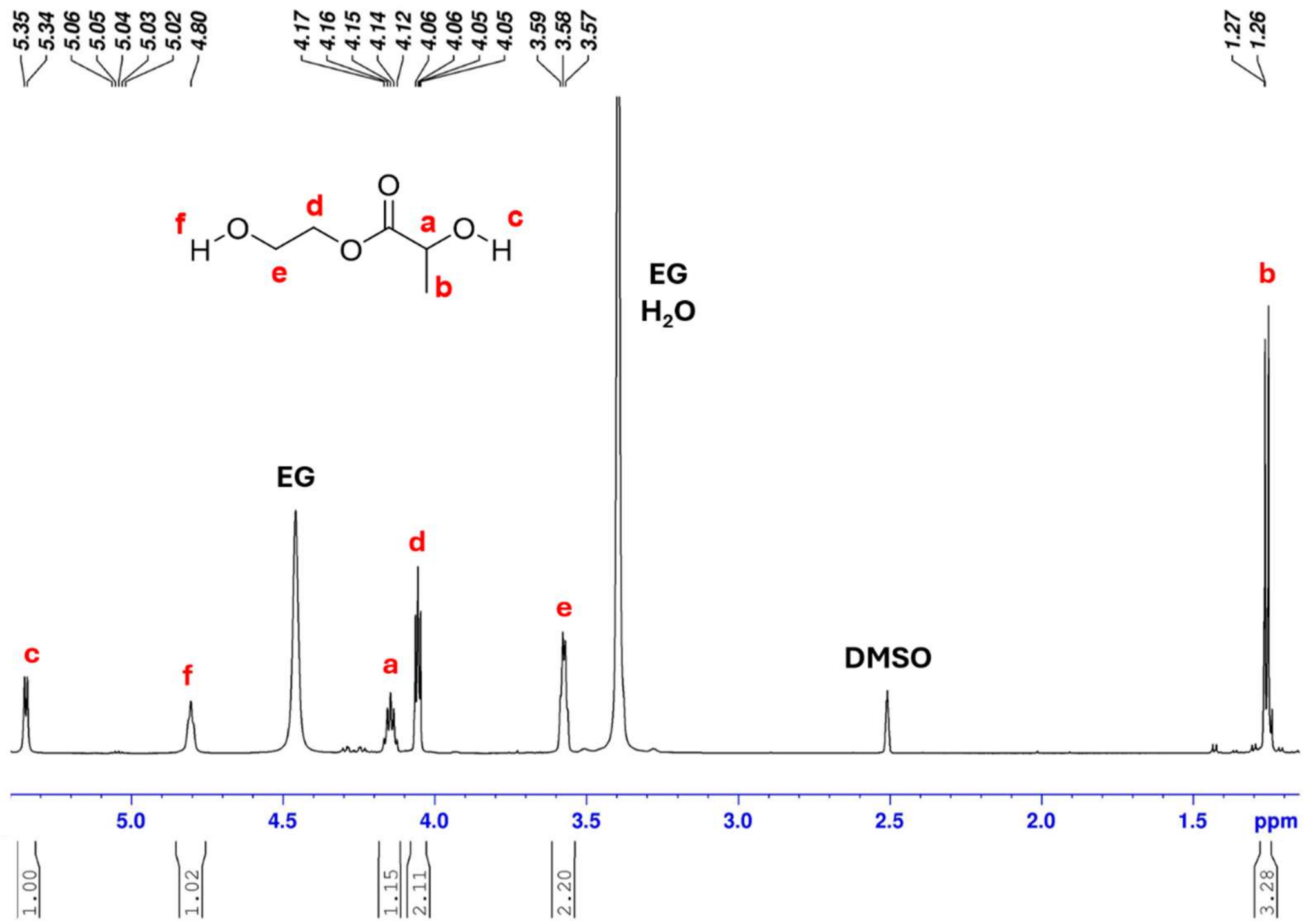

Figure S2. ^{1}H NMR spectrum (400 MHz, $CDCl_3$, 298 K) of glycolyl lactate (GlyLa).

^{1}H NMR (400 MHz, $CDCl_3$, 298 K): δ 5.35 (d, J = 6.8 Hz, 1H, Hc), 4.80 (m, 1H, Hf), 4.15 (quint, J = 6.6 Hz, 1H, Ha), 4.05 (t, d = Hz, 2H, Hd), 3.58 (m, 2H, He), 1.27 (d, J = 6.6 Hz, 3H, Hb).

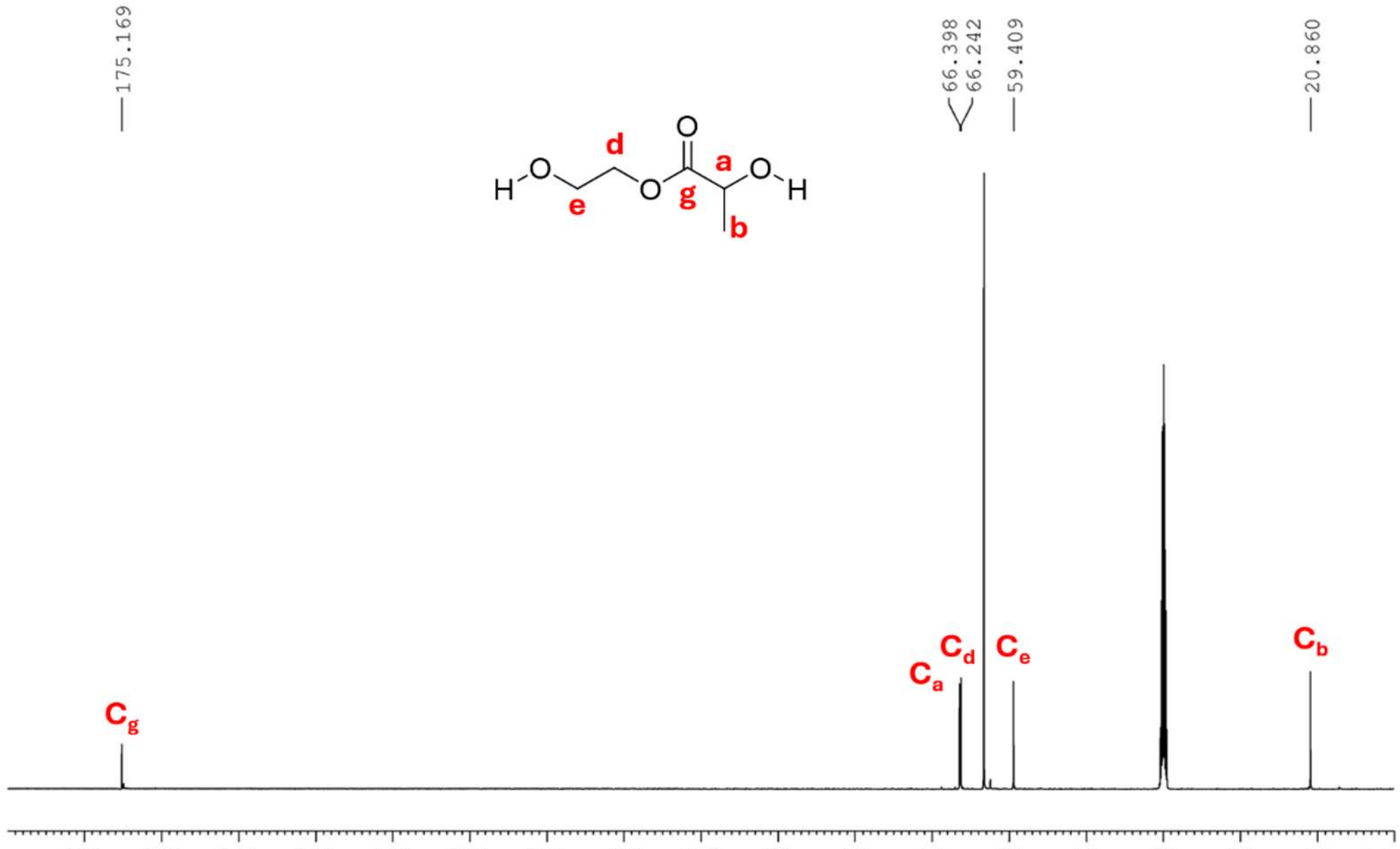

Figure S3. ^{13}C NMR spectrum (150 MHz, DMSO, 298 K) of glycolyl lactate.

^{13}C NMR (150 MHz, DMSO, 298 K): δ 175.17 (C_g), 66.40 (C_a), 66.24 (C_d), 59.41 (C_e), 20.86 (C_b).

- Alcoholysis by 1,4-butanediol (1,4-BDO)

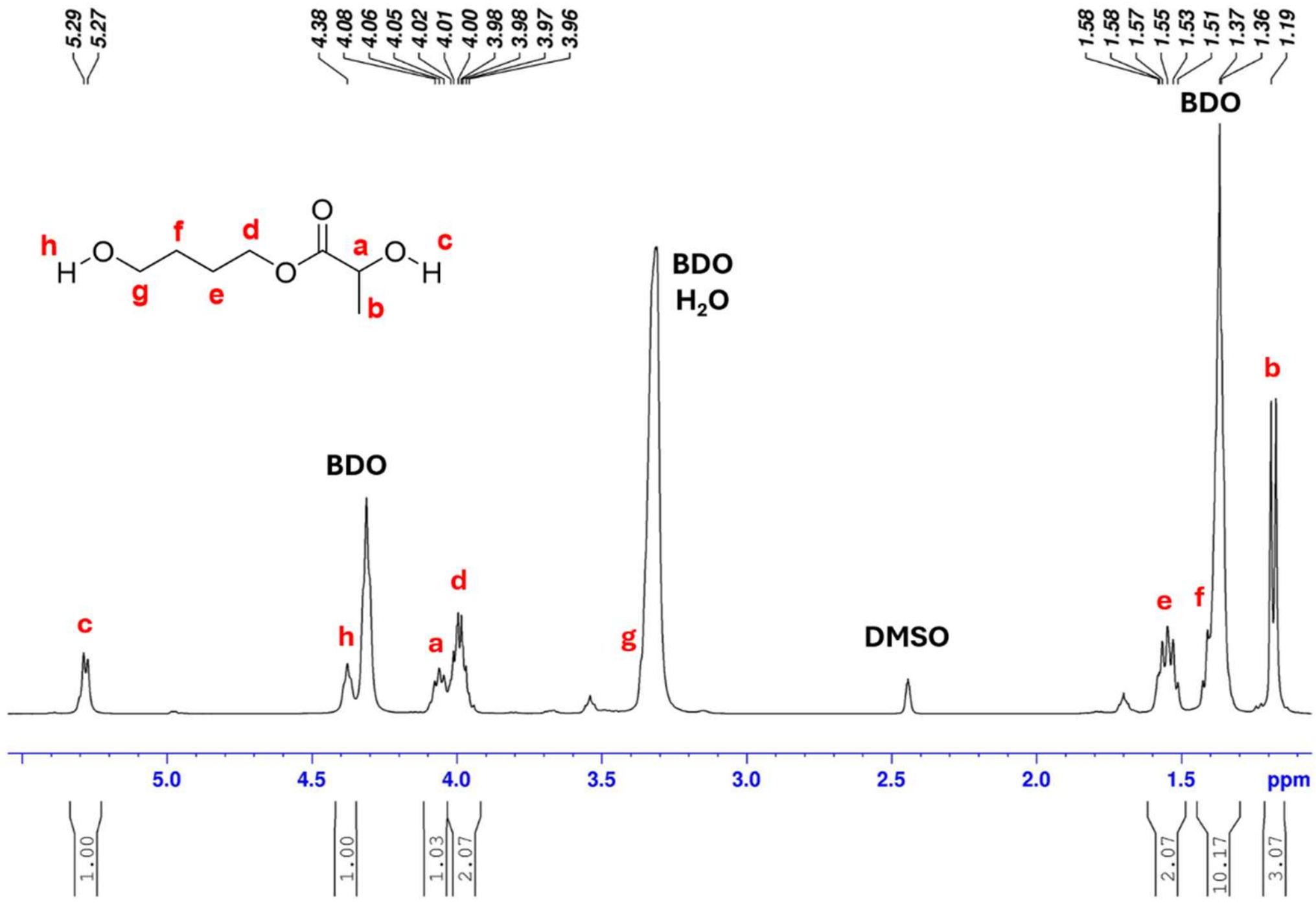

Figure S4. ^{1}H NMR spectrum (400 MHz, $CDCl_3$, 298 K) of 4-hydroxybutyl lactate (BDLa).
^{1}H NMR (400 MHz, $CDCl_3$, 298 K): δ 5.28 (m, 1H, Hc), 4.38 (m, 1H, Hh), 4.06 (quint, J = 6.6 Hz, 1H, Ha), 3.98 (m, 2H, Hd), 3.32 (m, 2H, Hg), 1.58 (m, 2H, He), 1.38 (m, 2H, Hf), 1.19 (d, J = 6.6 Hz, 3H, Hb).

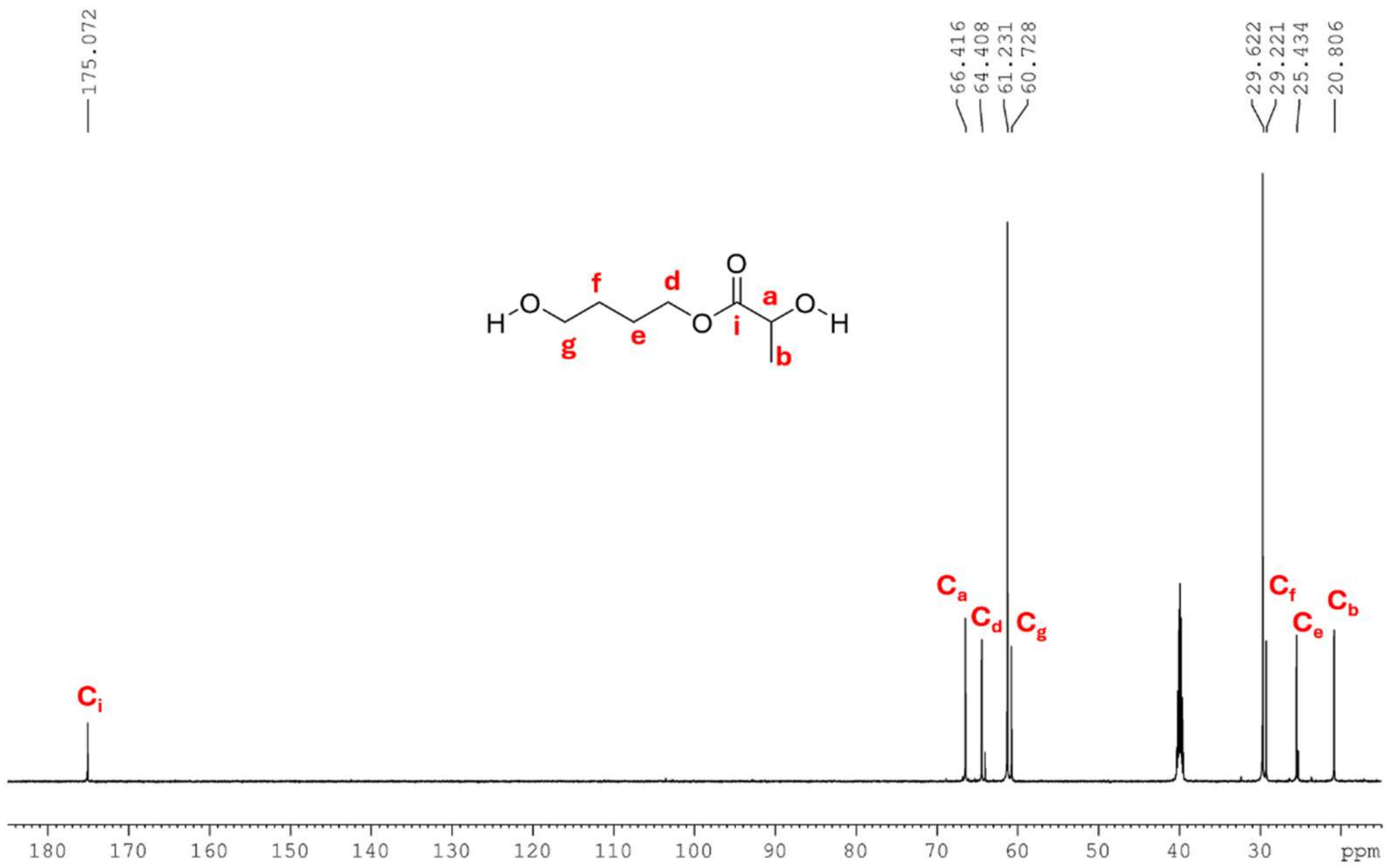

Figure S5. ^{13}C NMR spectrum (150 MHz, DMSO, 298 K) of 4-hydroxybutyl lactate (BDLa).
^{13}C NMR (150 MHz, DMSO, 298 K): δ 175.07 (C_i), 66.41 (C_a), 64.41(C_d), 60.73 (C_g), 29.22 (C_f), 25.43 (C_e), 20.81 (C_b).

- Alcoholysis by *cis*-2-butene-1,4-diol (*cis*-BD)

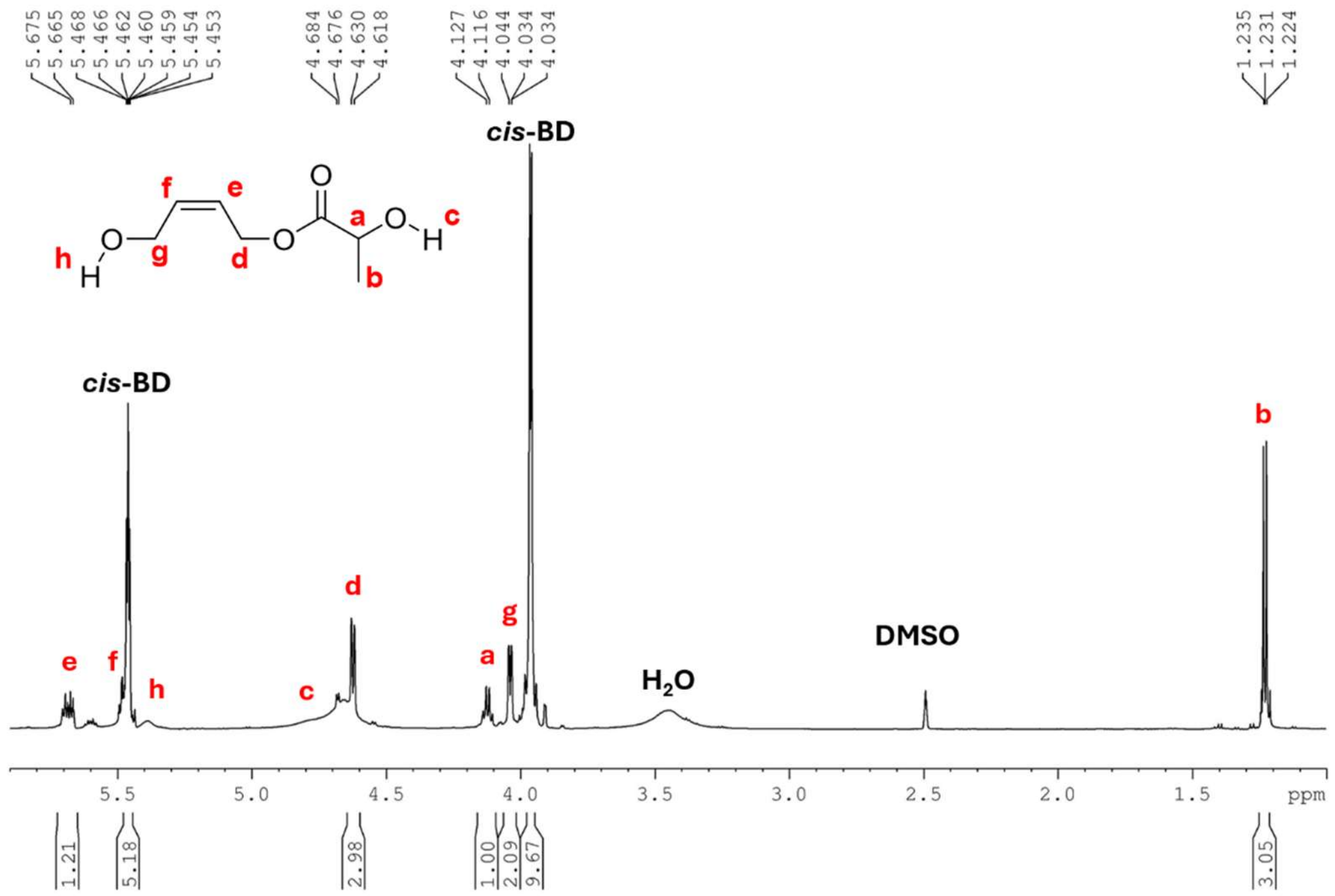

Figure S6. ^{1}H NMR spectrum (400 MHz, $CDCl_3$, 298 K) of 4-hydroxy-*cis*-butyl lactate (*c*BDLa). ^{1}H NMR (600 MHz, $CDCl_3$, 298 K): δ 5.68 (m, 1H, He), 5.46 (m, 2H, Hf + Hh), 4.75 (br, 1H, Hc), 4.63 (d, J = 7.2 Hz, 2H, Hd), 4.12 (q, J = 6.6 Hz, 1H, Ha), 4.03 (d, J = 6.0 Hz, 2H, Hg), 1.23 (d, J = 6.6 Hz, 3H, Hb).

- Alcoholysis by 3-allyloxy-1,2-propanediol (APD)

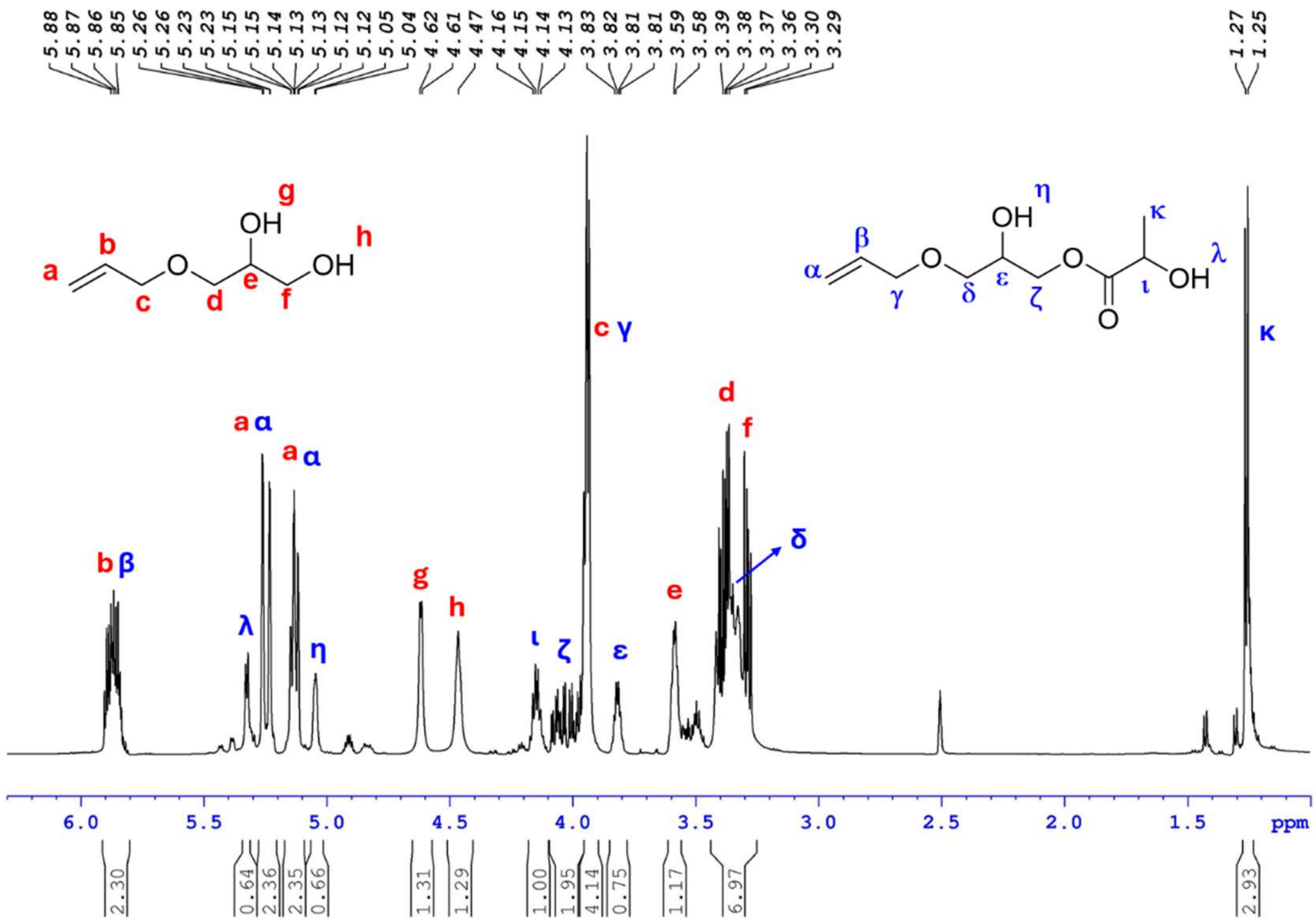

Figure S7. ^{1}H NMR spectrum (DMSO, 600 MHz, 298 K) of ADP-lactate (ADPLa).

Table S1. ^{1}H NMR assignment of APD-lactate (APDLa)

Chemical shift δH (ppm)	Integration	Multiplicity	Label	Assignment
5.88-5.86	1H	m	β	internal vinylic CH, CH_2=C*H*-
5.32	1H	br	λ	lactyl O*H*
5.28-5.23; 5.15-5.12	2H	d; d	α	terminal vinylic C*H*$_2$
5.05-5.04	1H	br	η	O*H* on APD-derived
4.16-4.13	1H	q/m	ι	lactyl C*H*(OH)CH_3
4.10-3.94	2H	m	ζ	C*H*$_2$-O-C(O) on APD-derived
3.83-3.81	2H	m	γ	O-C*H*$_2$ on APD-derived
3.70	1H	m	ε	C*H*-OH on APD-derived
3.39-3.29	2H	m	δ	O-C*H*$_2$-C(OH) on APD-derived
1.27-1.25	3H	d	κ	lactyl C*H*$_3$

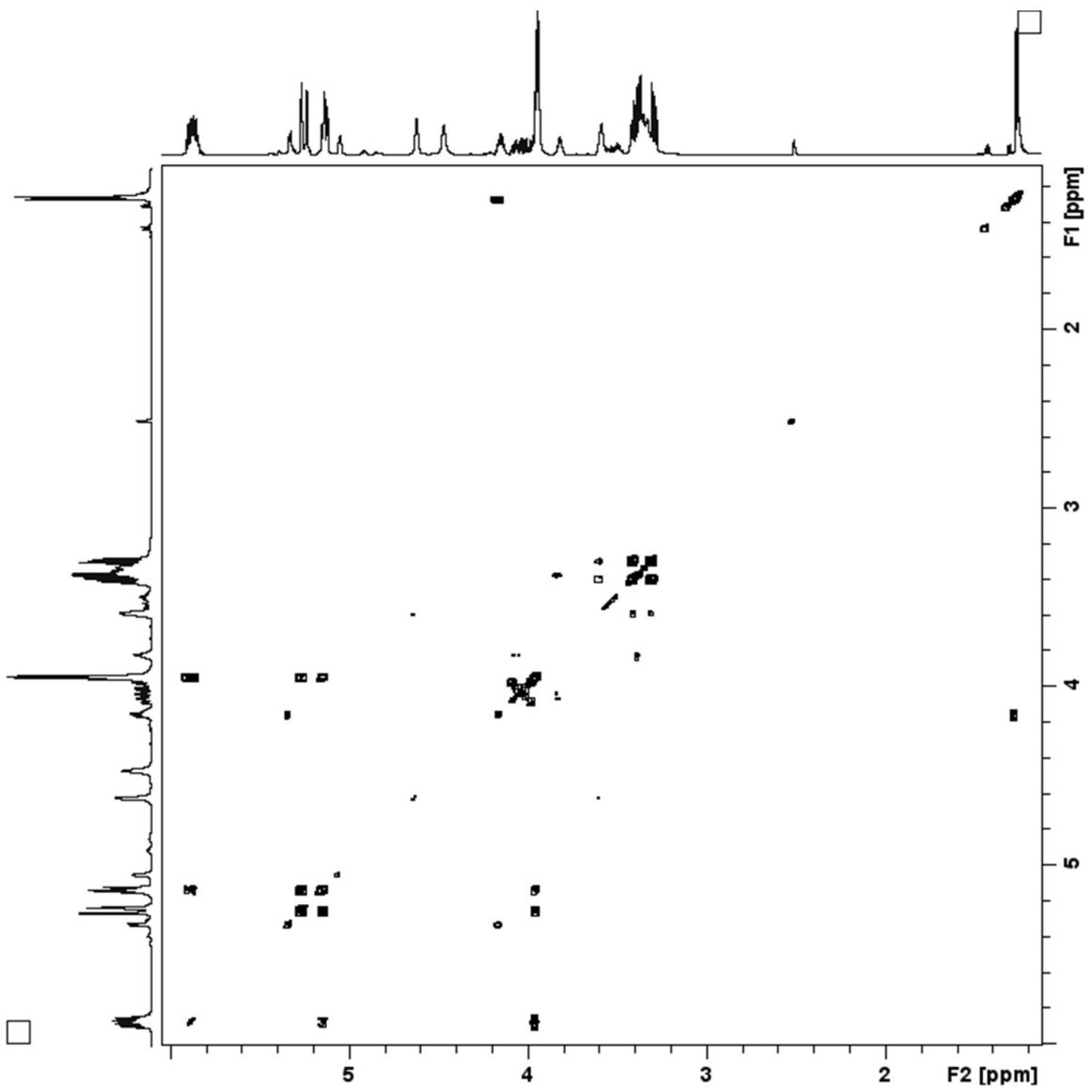

Figure S8. COSY NMR spectrum (600 MHz, DMSO, 298 K) of ADP-lactate (ADPLa).

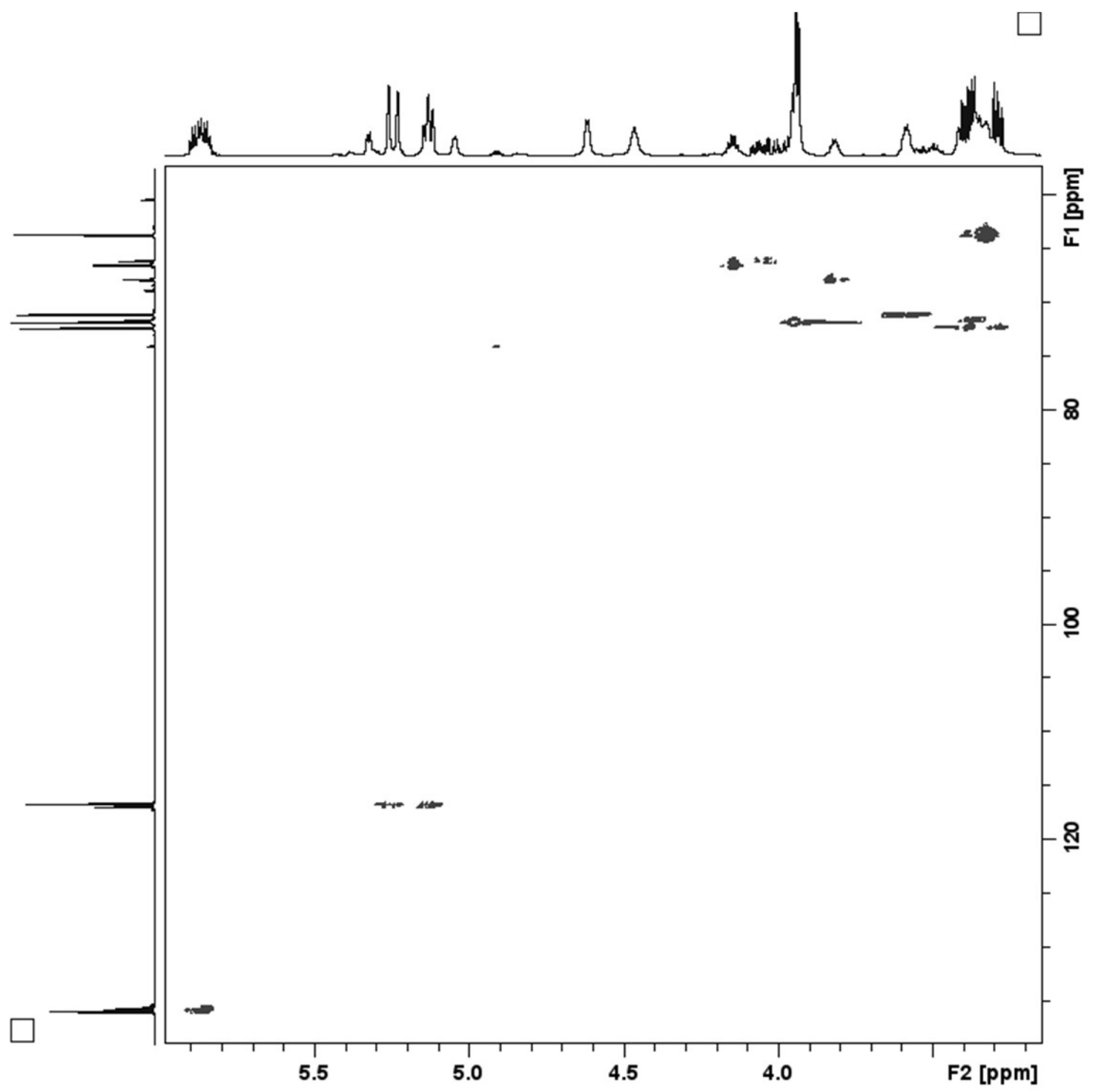

Figure S9. HSQC NMR spectrum (600 MHz, DMSO, 298 K) of ADP-lactate (ADPLa).

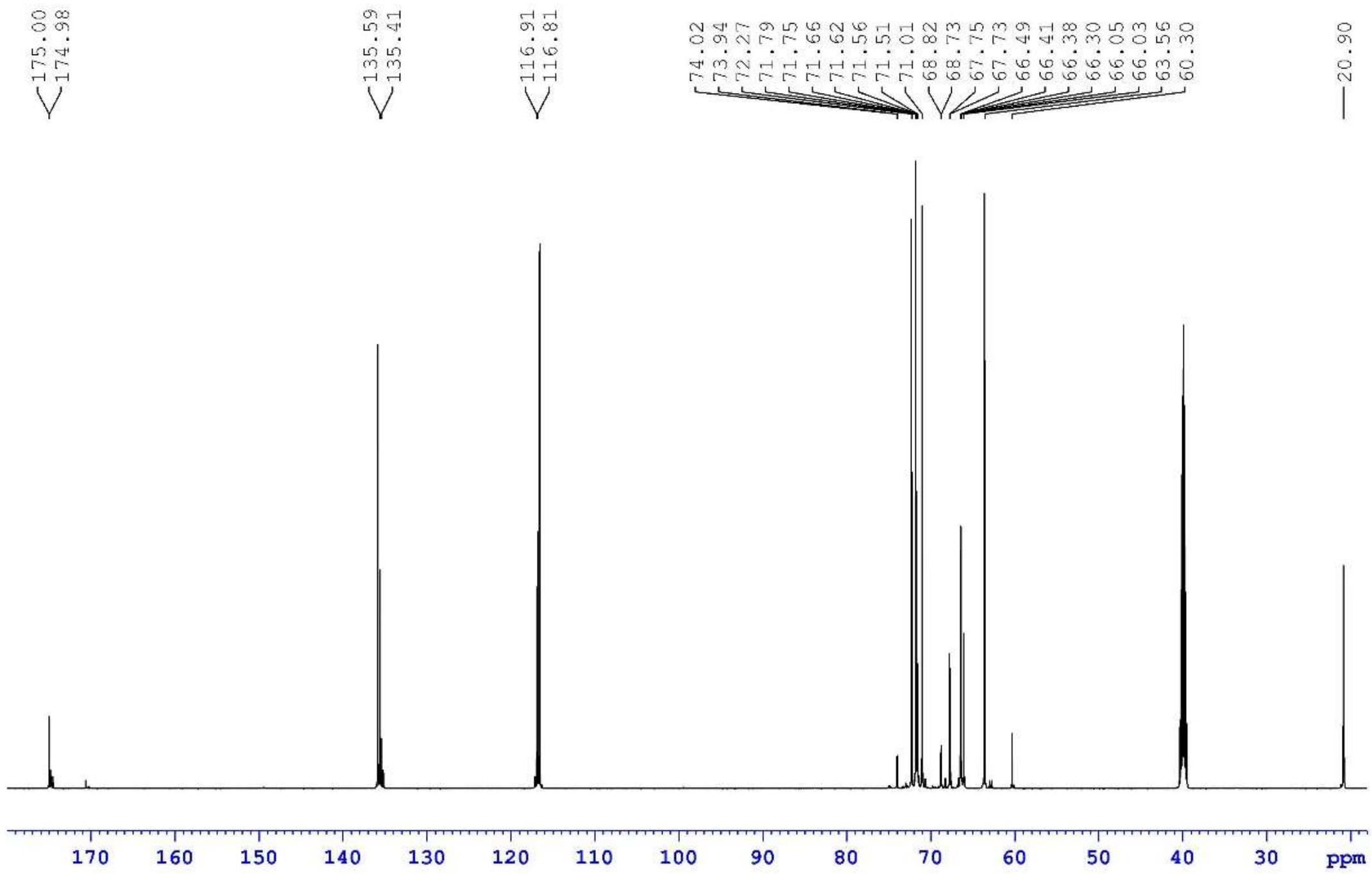

Figure S10. ^{13}C NMR spectrum (150 MHz, DMSO, 298 K) of ADP-lactate (ADPLa).

Characterization of the polycondensation products

- *Ethylene glycol (EG)*

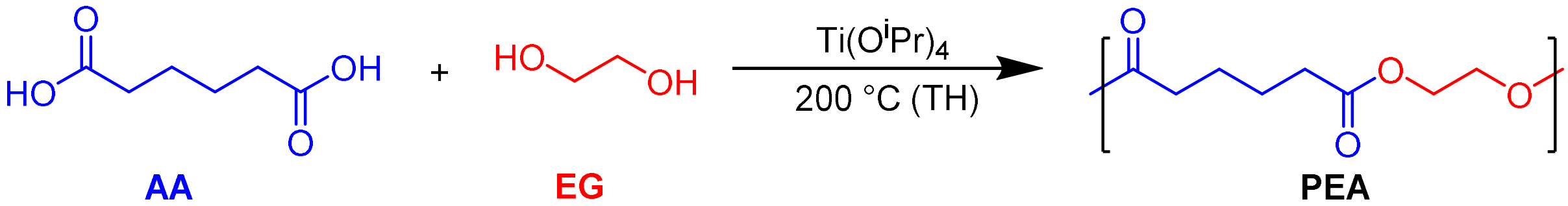

Scheme S1. Polycondensation reaction of the adipic acid (AA) and ethylene glycol (EG).

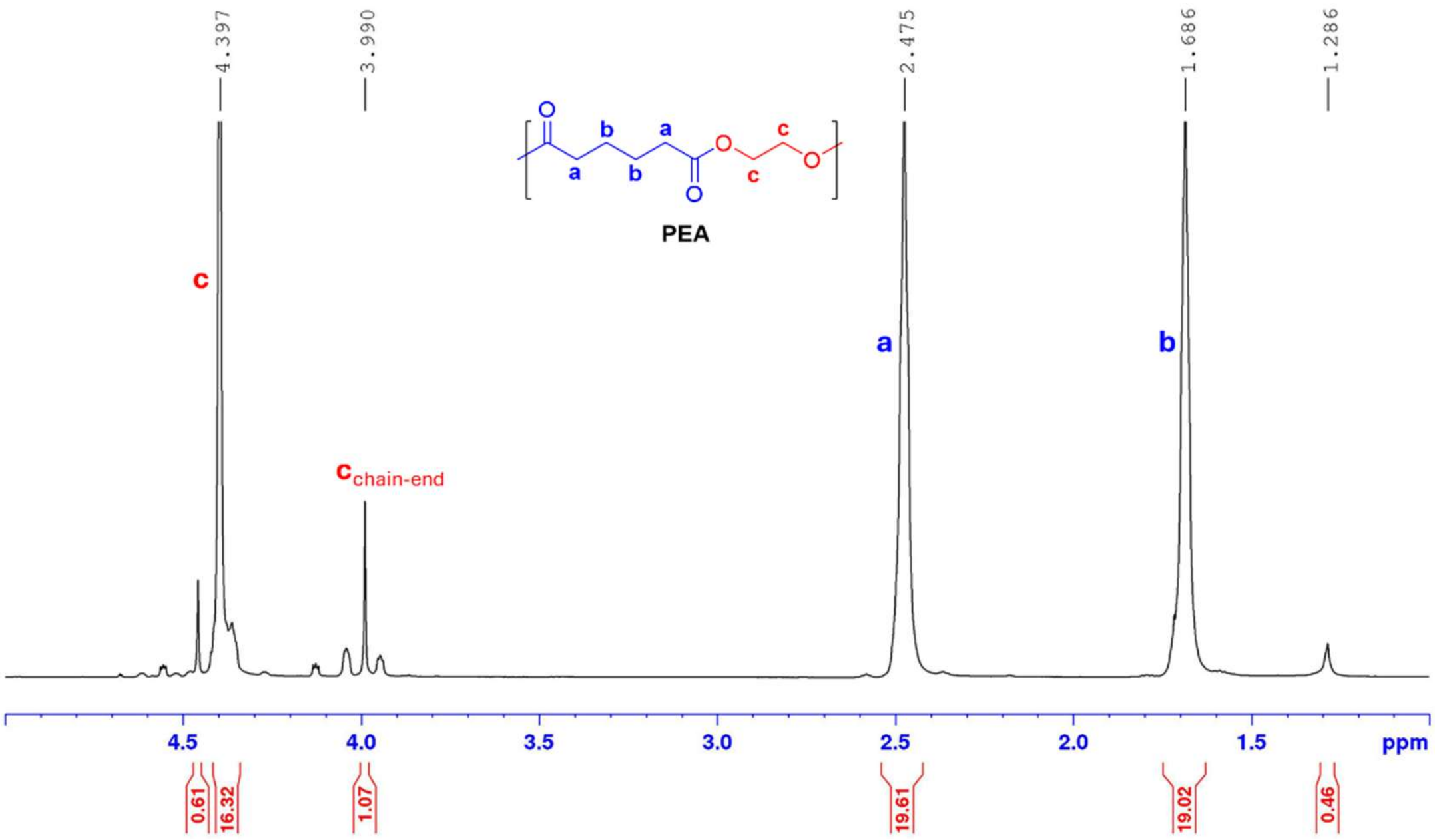

Figure S11. ^{1}H NMR spectrum (600 MHz, $CDCl_3$, 298 K) of poly(ethylene)adipate, PEA.

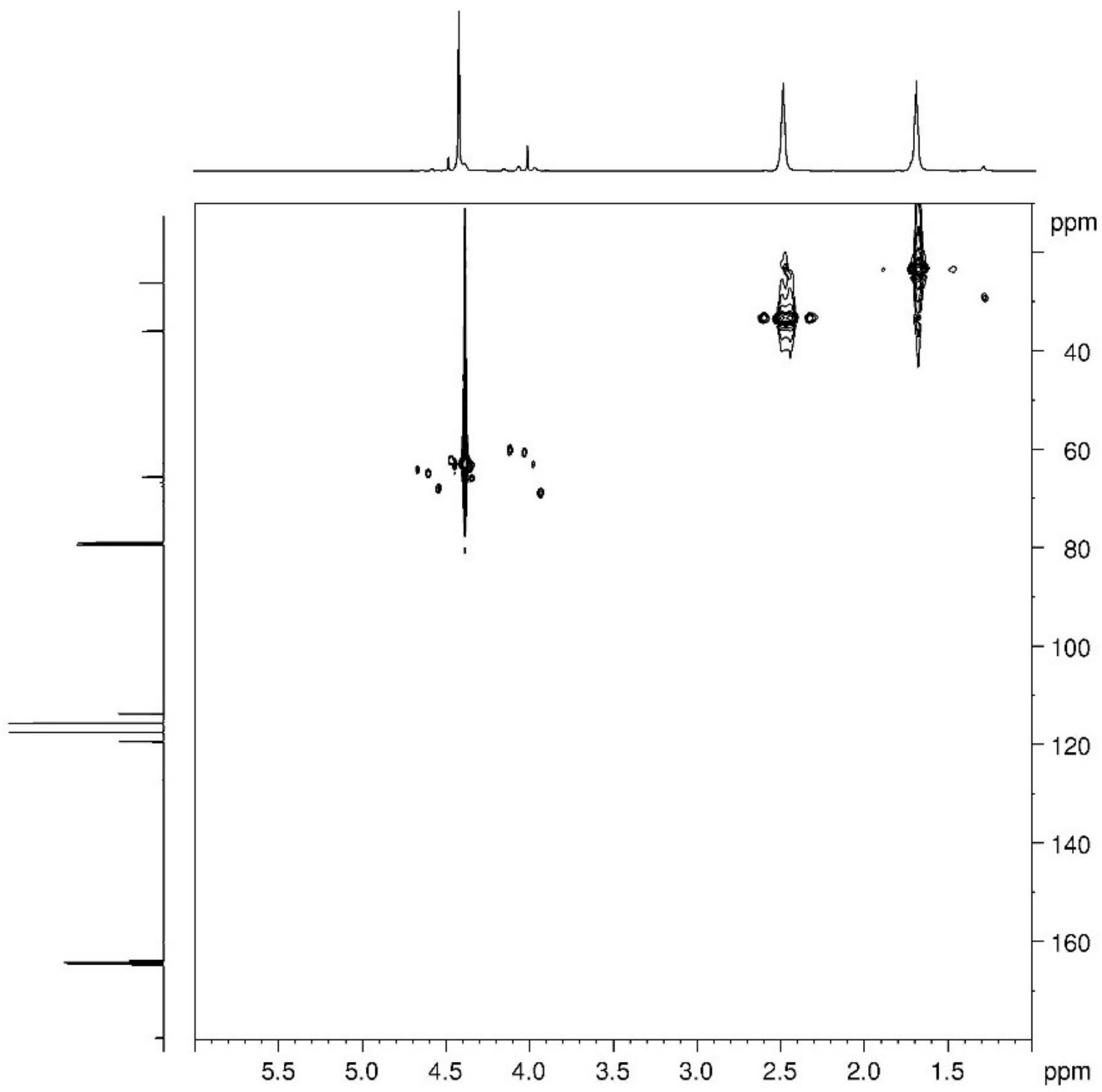

Figure S12. HSQC NMR spectrum (600 MHz, $CDCl_3$, 298 K) of poly(ethylene)adipate, PEA.

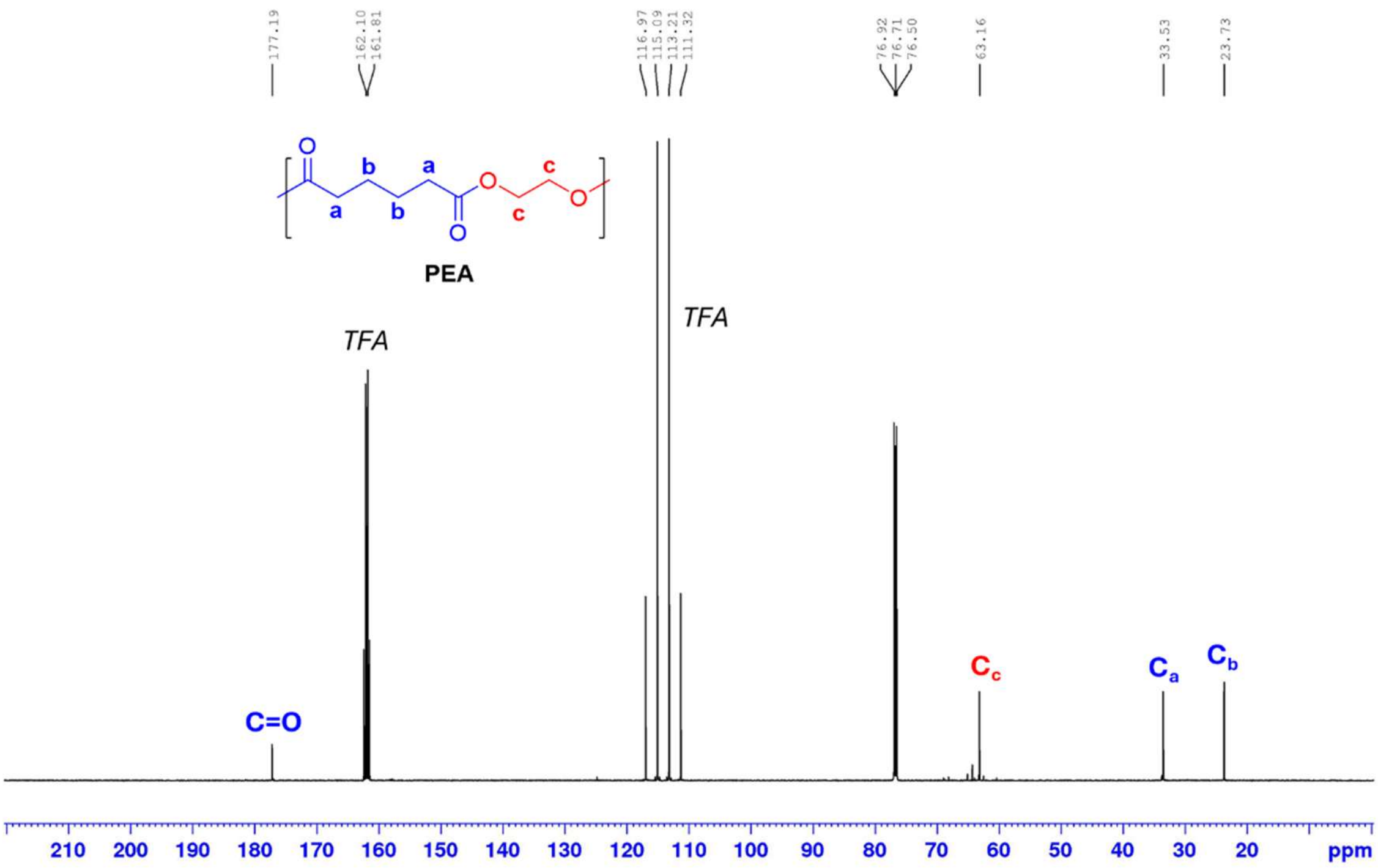

Figure S13. ^{13}C NMR spectrum (150 MHz, $CDCl_3$, 298 K) of poly(ethylene)adipate, PEA. *TFA= trifluoroacetic acid.*

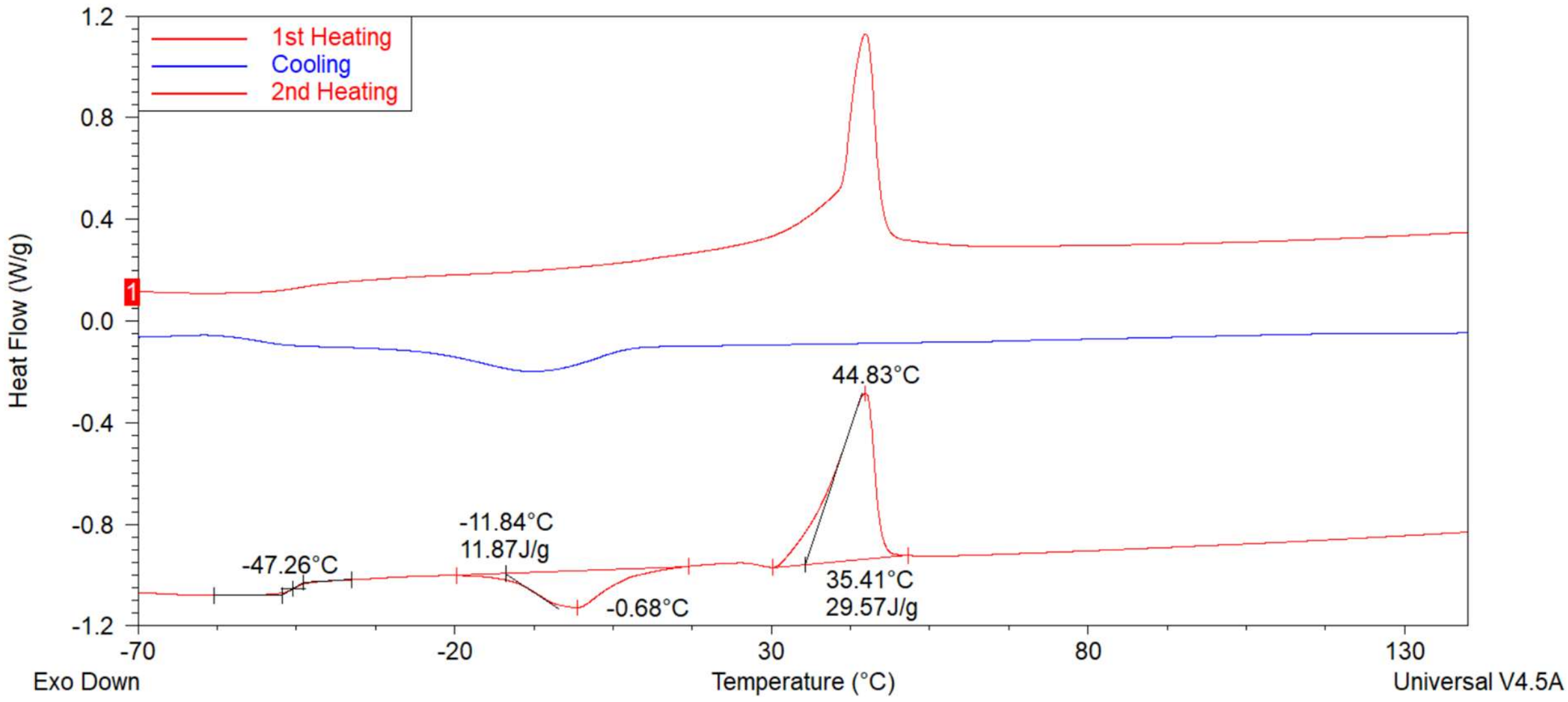

Figure S14. DSC of poly(ethylene)adipate, PEA.

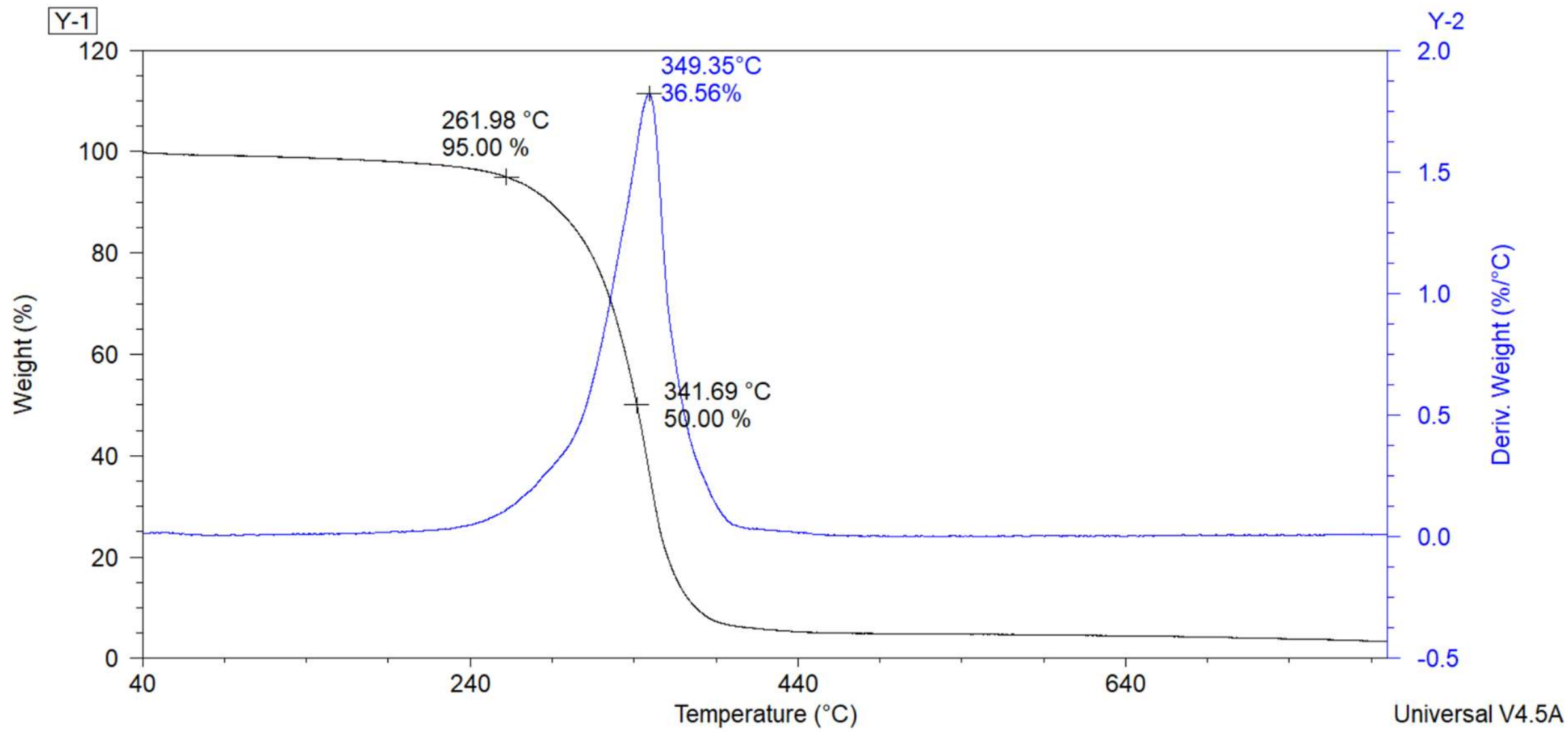

Figure S15. TGA of poly(ethylene)adipate, PEA.

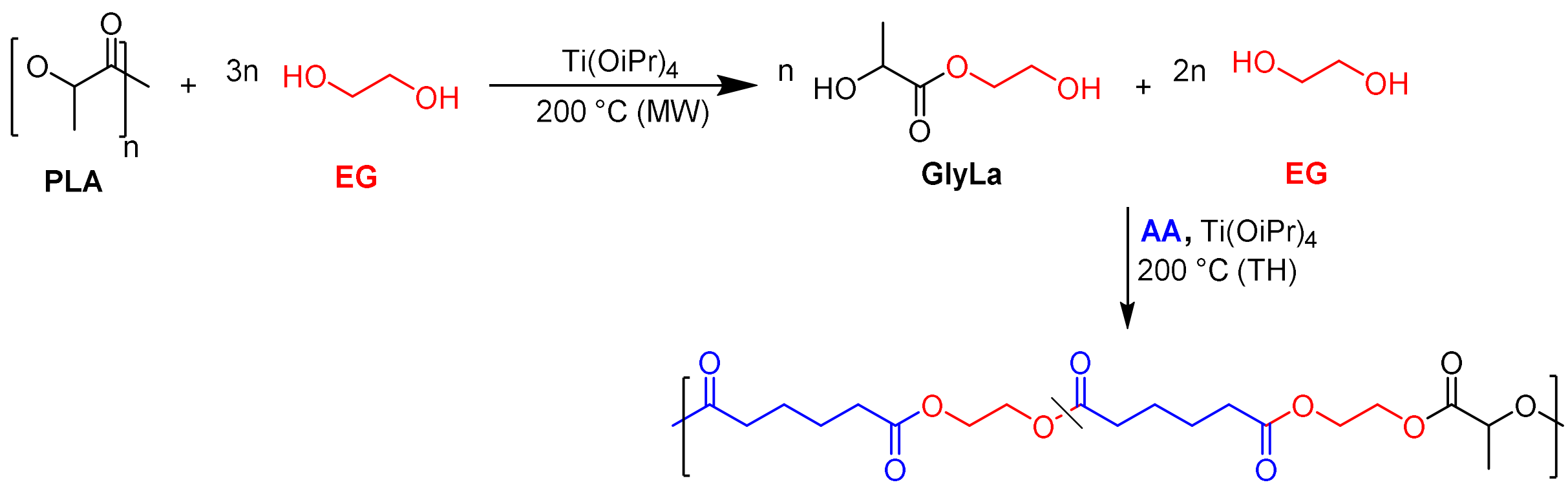

Scheme S2. Polycondensation reaction of the adipic acid (AA) with the mixture ethylene glycol (EG) and glycolyl lactate (GlyLa).

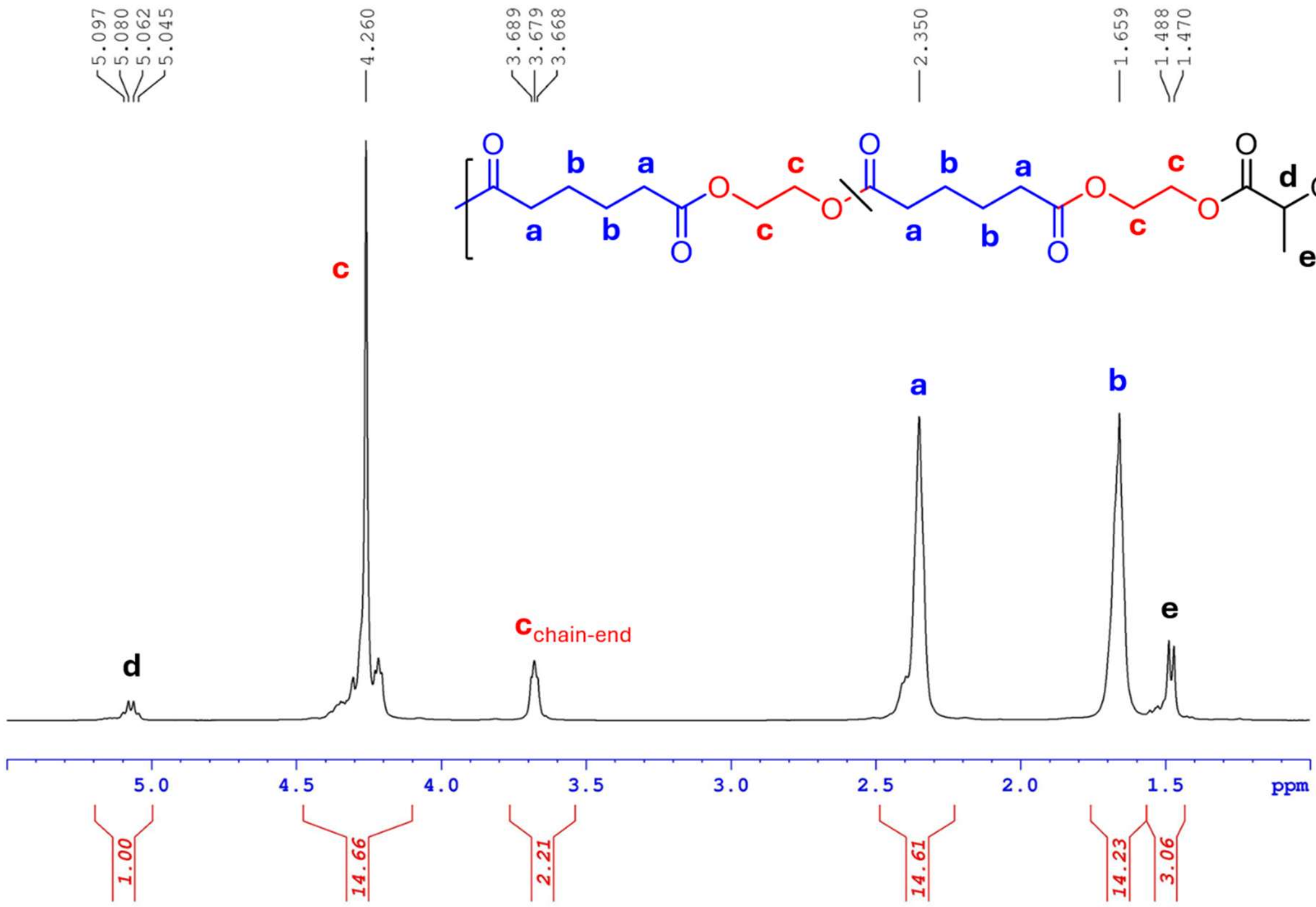

Figure S16. ^{1}H NMR spectrum (600 MHz, $CDCl_3$, 298 K) of poly(ethylene adipate-*co*-lactate), PEALa.

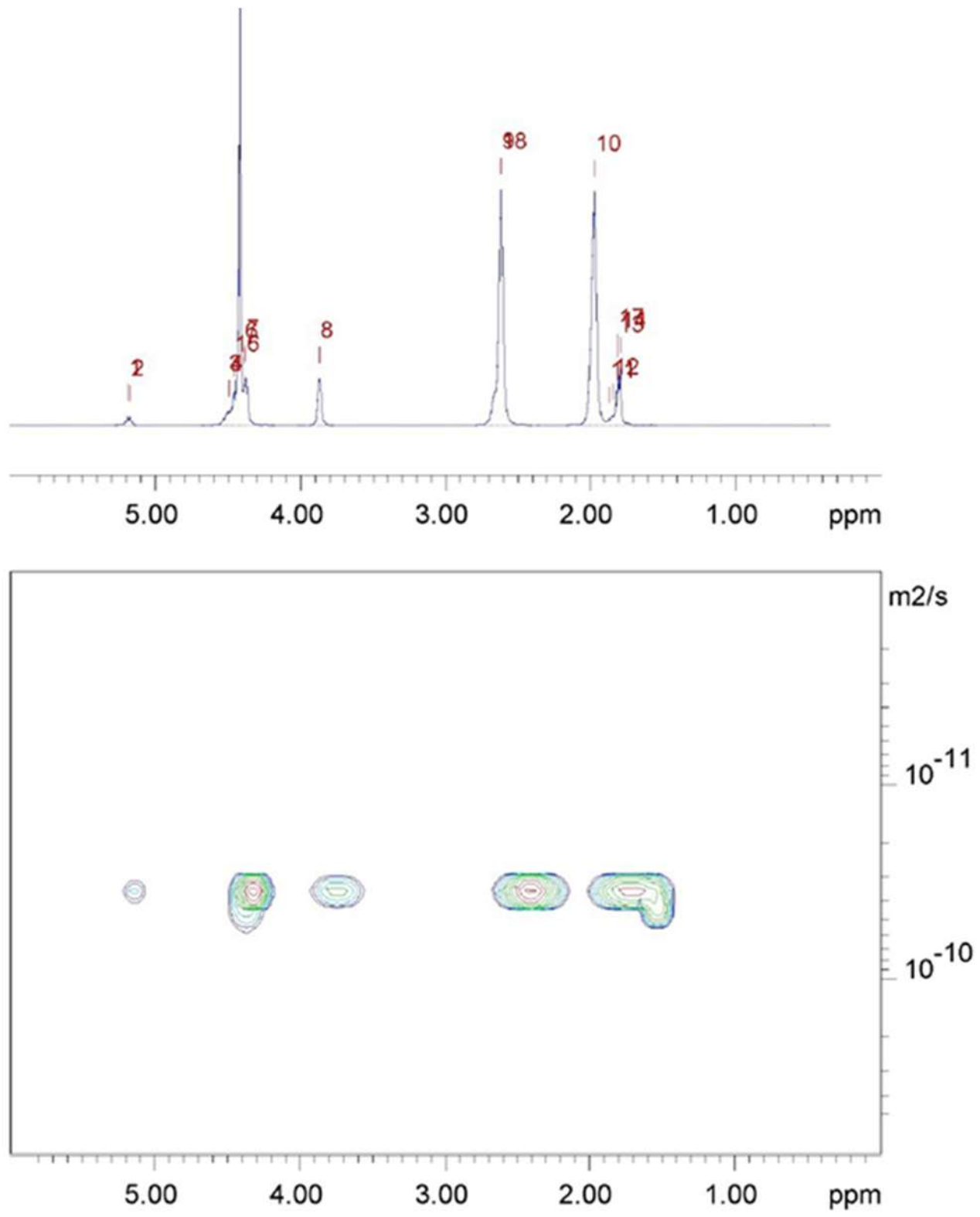

Figure S17. 2D DOSY NMR spectrum (600 MHz, $CDCl_3$, 298 K) of poly(ethylene adipate-*co*-lactate), PEALa.

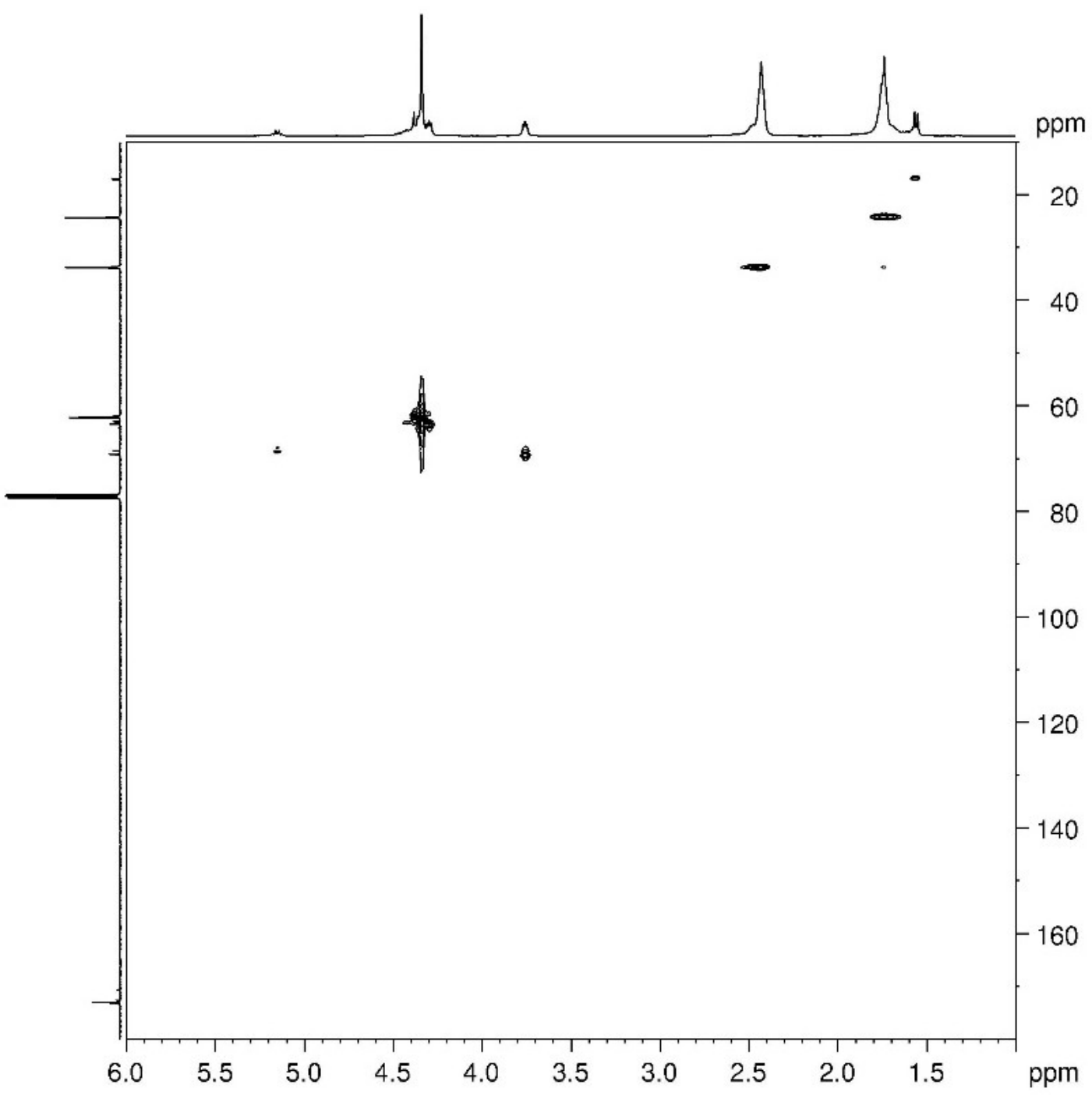

Figure S18. HSQC NMR spectrum (600 MHz, $CDCl_3$, 298 K) of poly(ethylene adipate-*co*-lactate), PEALa.

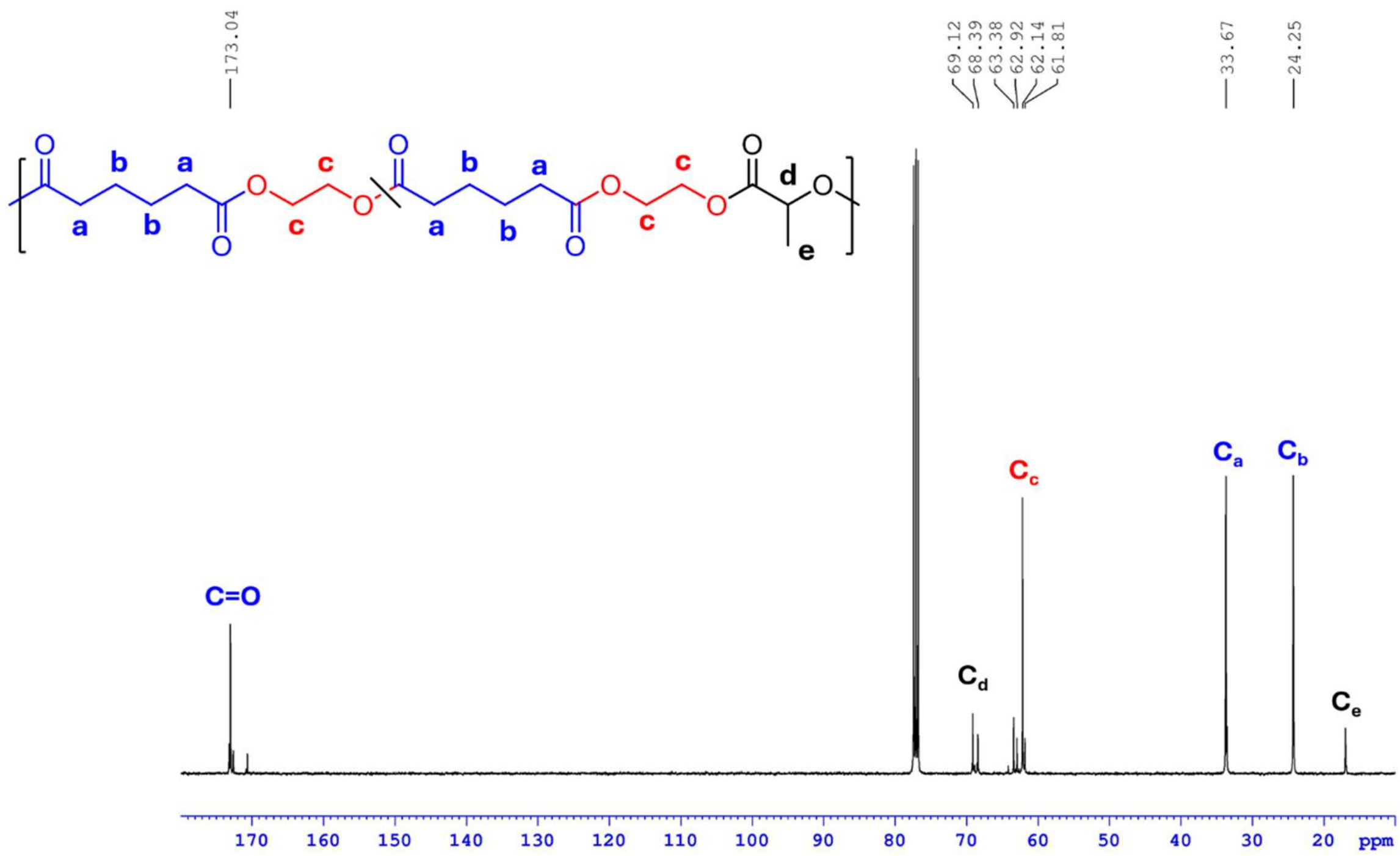

Figure S19. ^{13}C NMR spectrum (150 MHz, $CDCl_3$, 298 K) of poly(ethylene adipate-*co*-lactate), PEALa.

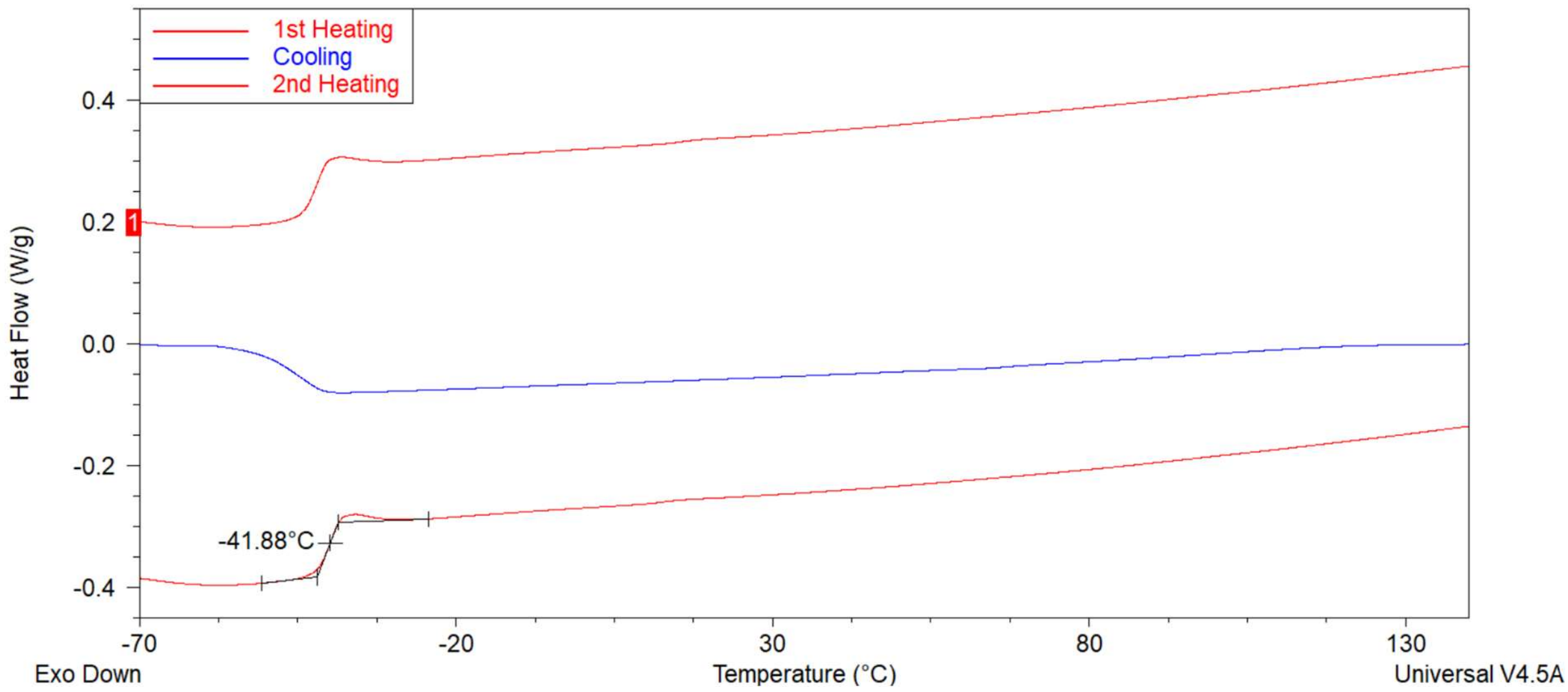

Figure S20. DSC of poly(ethylene adipate-*co*-lactate), PEALa.

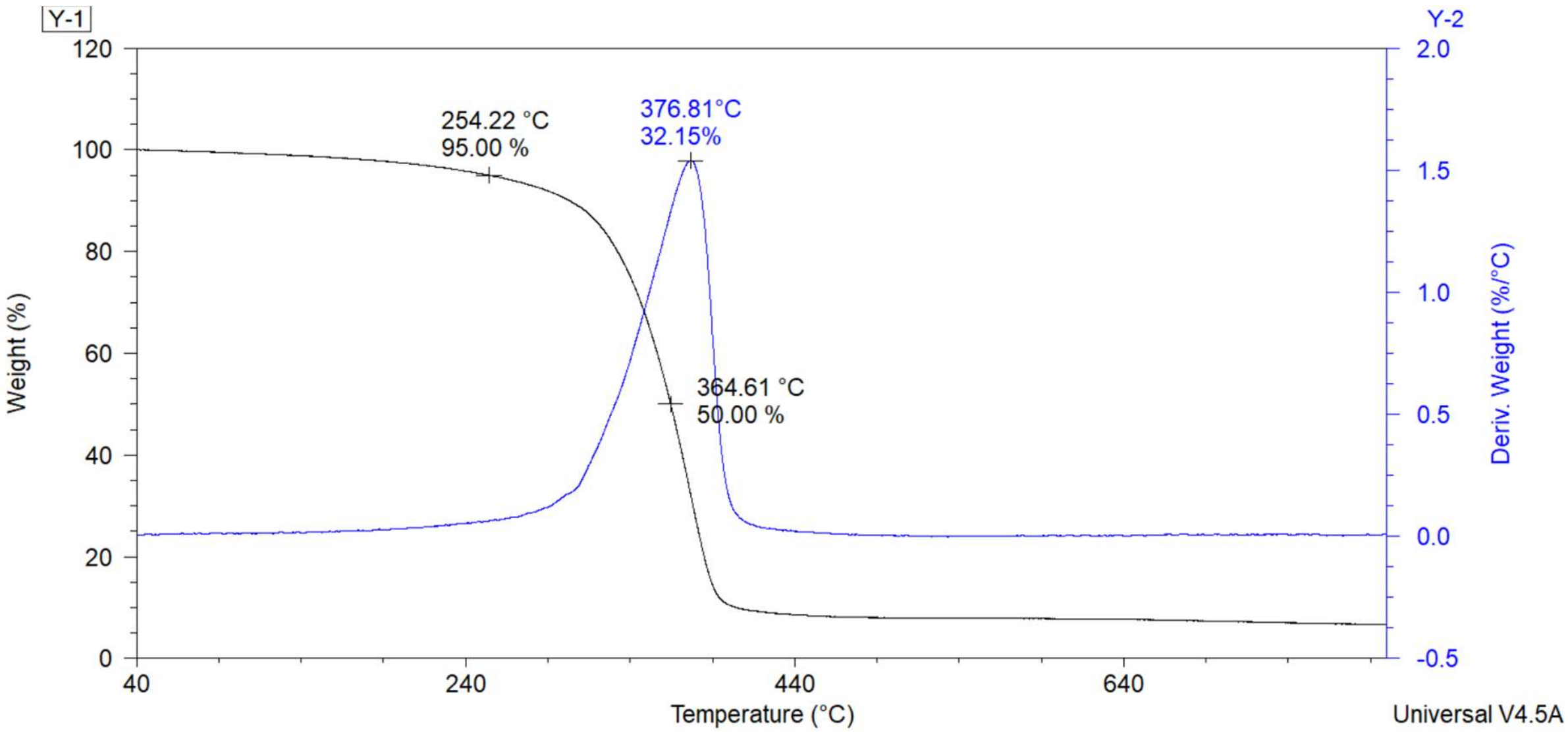

Figure S21. TGA of poly(ethylene adipate-*co*-lactate), PEALa.

- *1,4 butane diol (BD)*

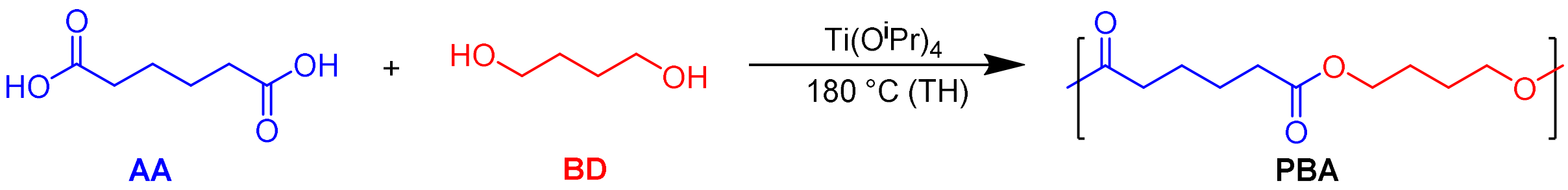

Scheme S3. Polycondensation reaction of the adipic acid (AA) and 1,4 butane diol (BD).

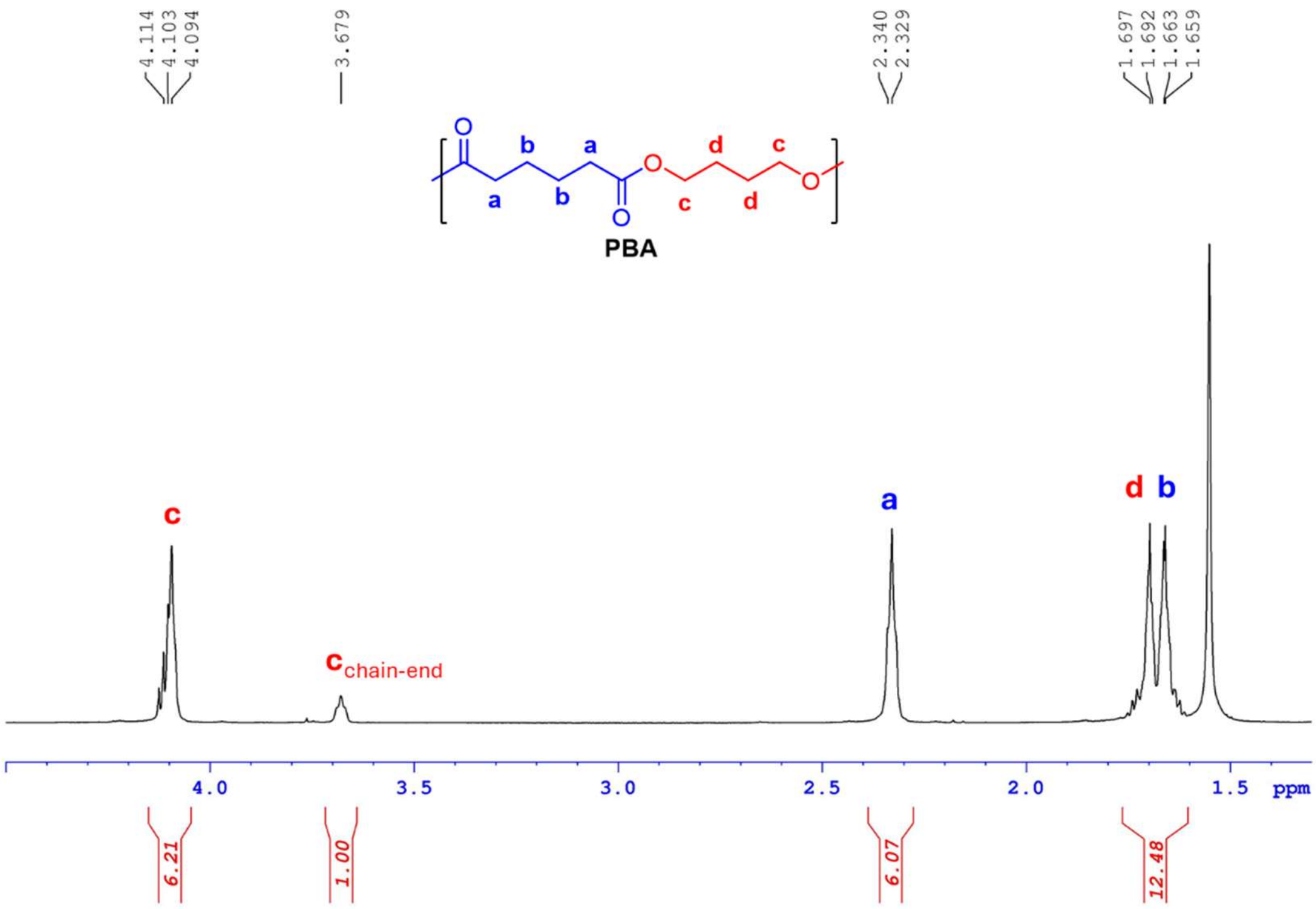

Figure S22. ^{1}H NMR spectrum (600 MHz, $CDCl_3$, 298 K) of poly(butylene)adipate, PBA.

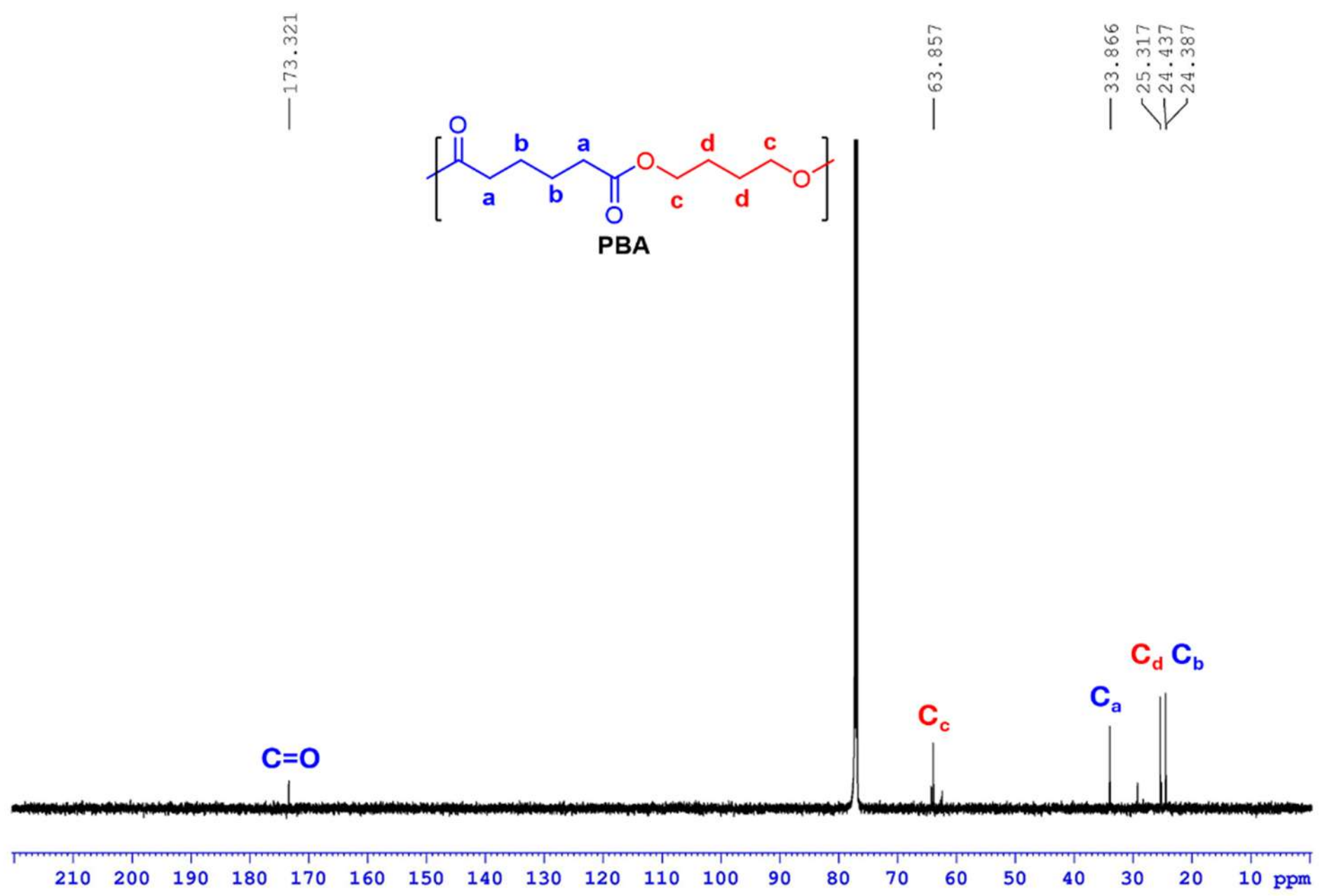

Figure S23. ^{13}C NMR spectrum (150 MHz, $CDCl_3$, 298 K) of poly(butylene)adipate, PBA.

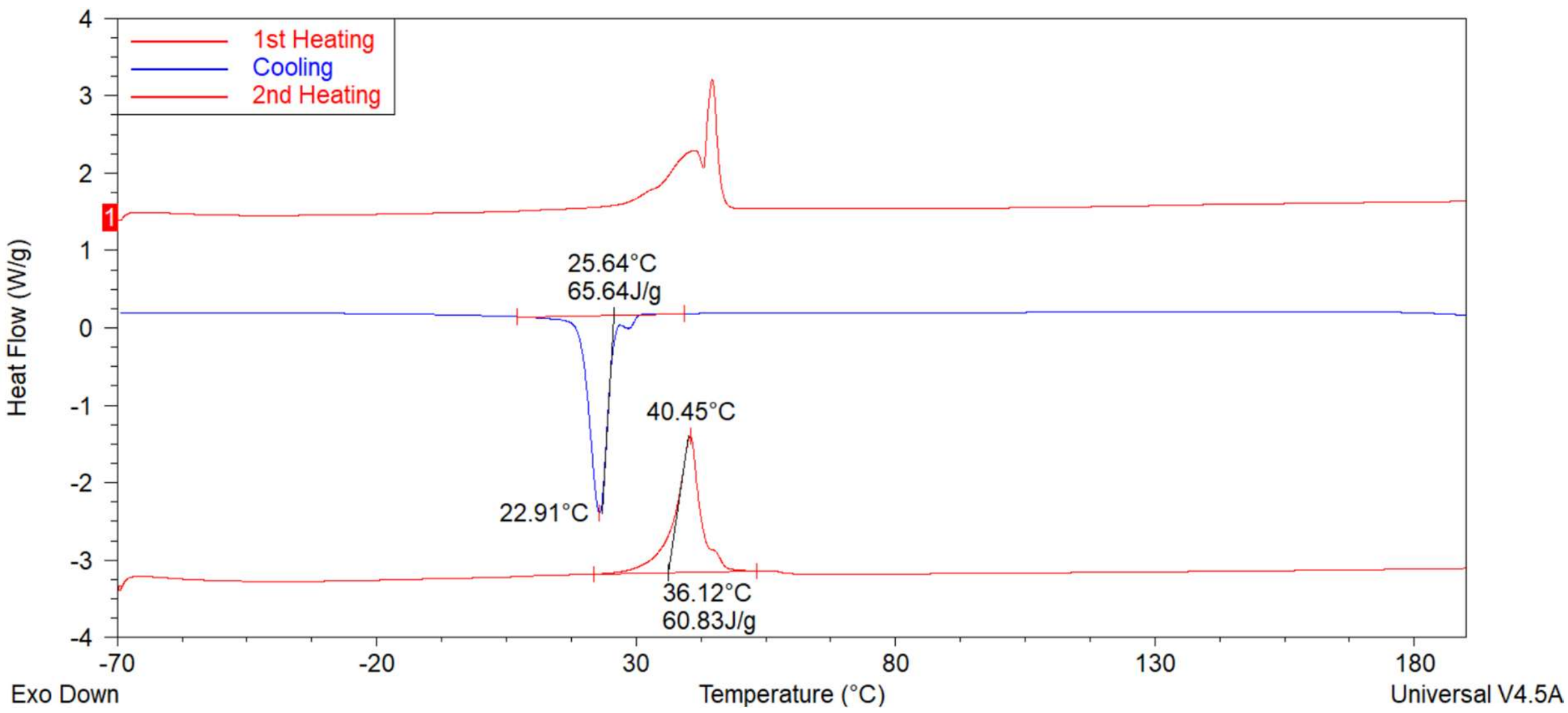

Figure S24. DSC of poly(butylene)adipate, PBA.

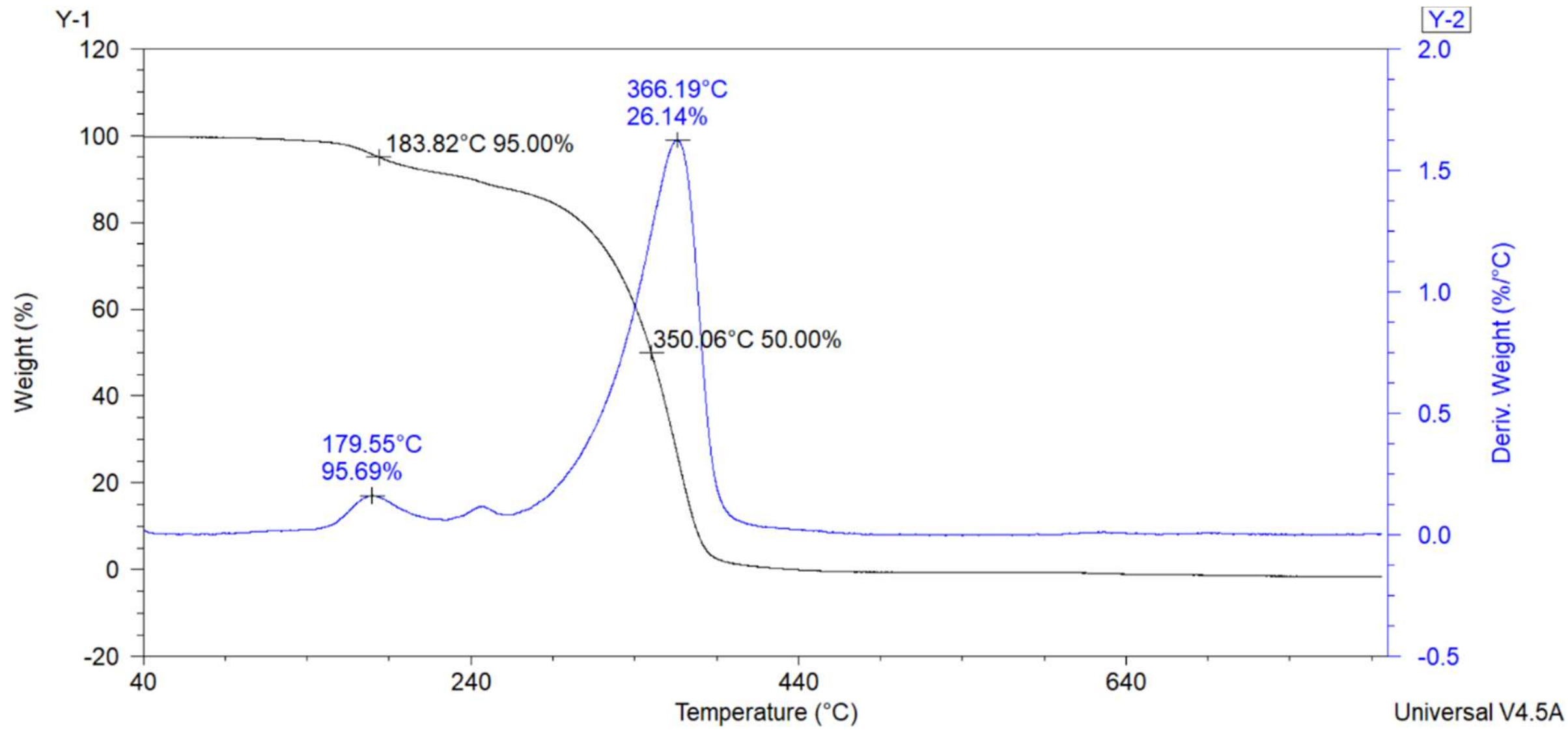

Figure S25. TGA of poly(butylene)adipate, PBA.

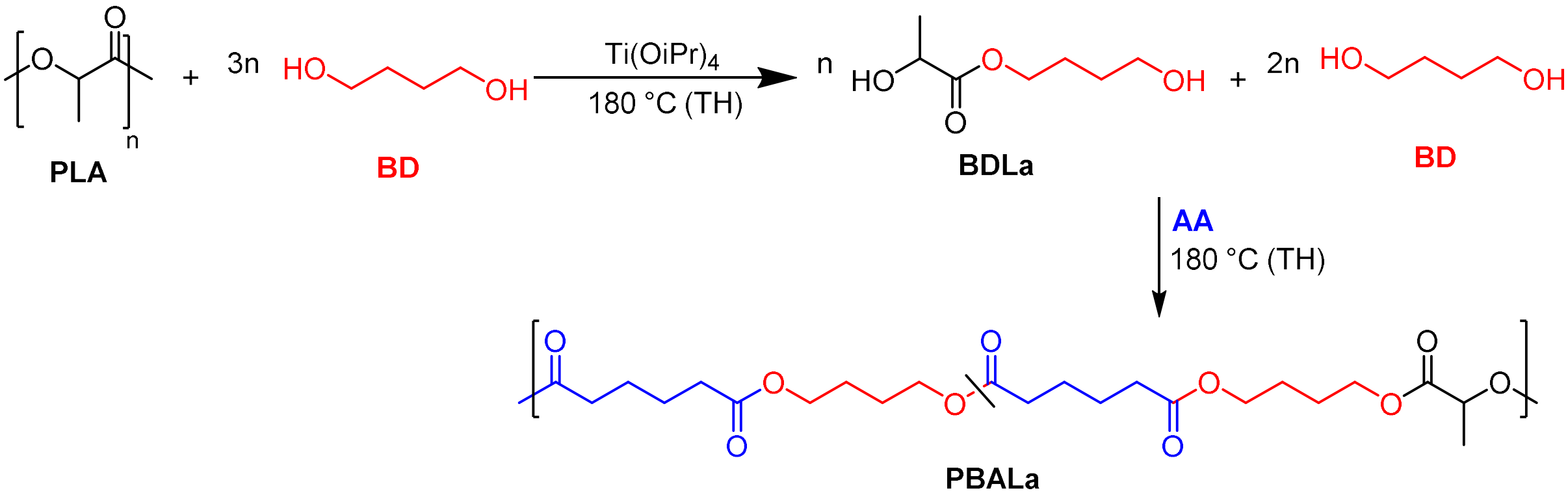

Scheme S4. Polycondensation reaction of the adipic acid (AA) with the mixture of 1,4 butane diol (BD) and 4-hydroxybutyl lactate (BDLa).

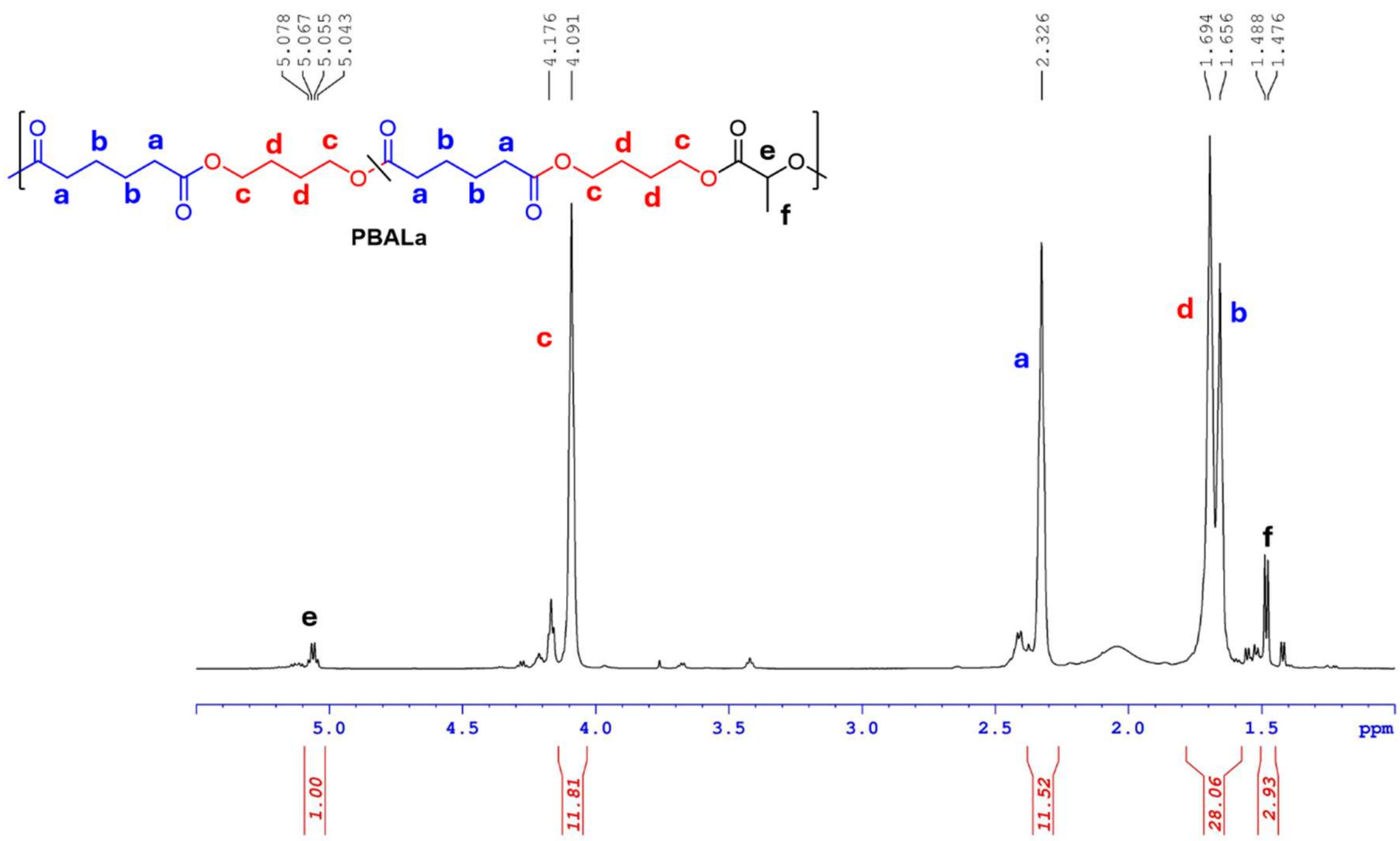

Figure S26. ^{1}H NMR spectrum (600 MHz, $CDCl_3$, 298 K) of poly(butylene adipate-*co*-lactate), PBALa.

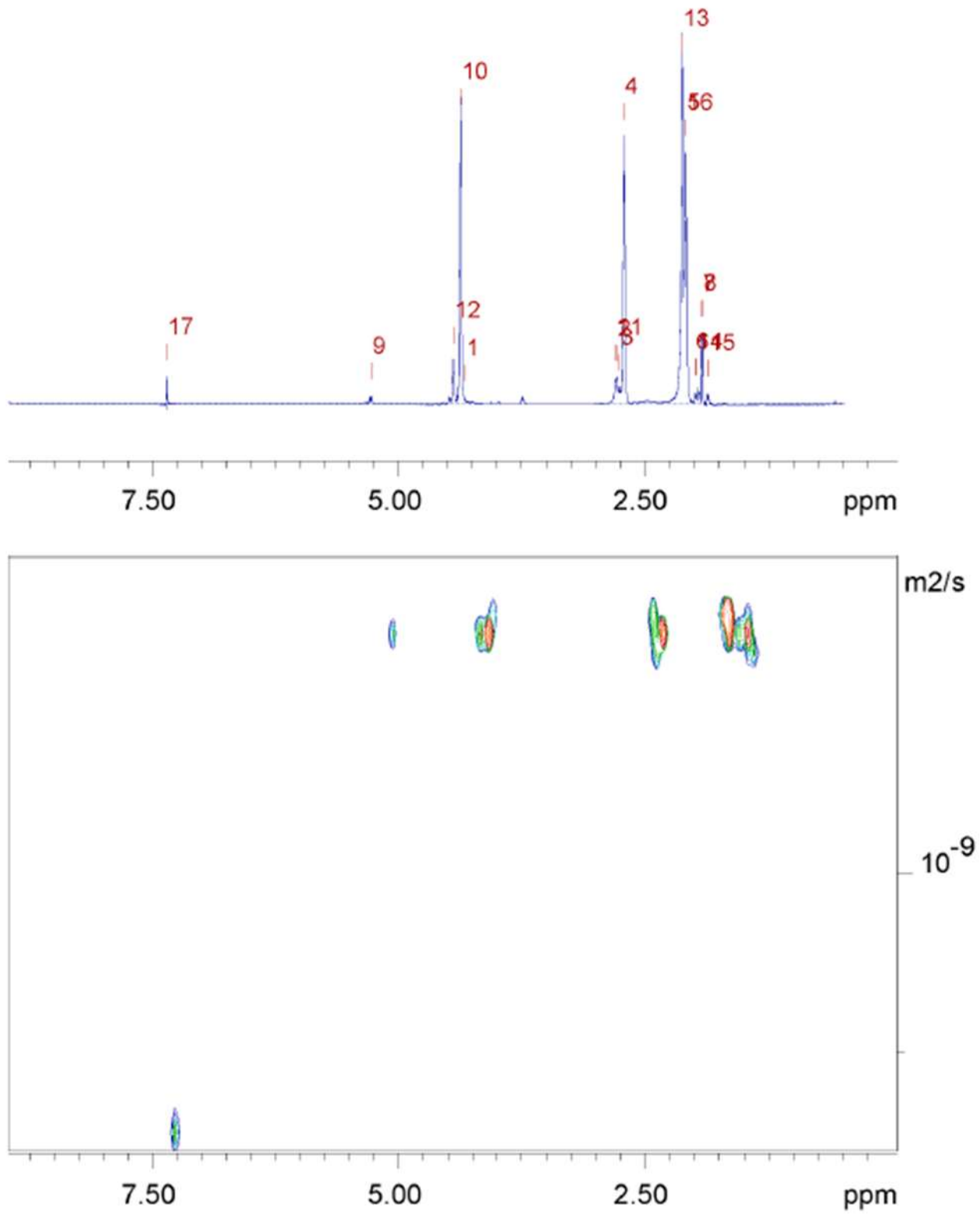

Figure S27. 2D DOSY spectrum (600 MHz, $CDCl_3$, 298 K) of poly(butylene adipate-*co*-lactate), PBALa.

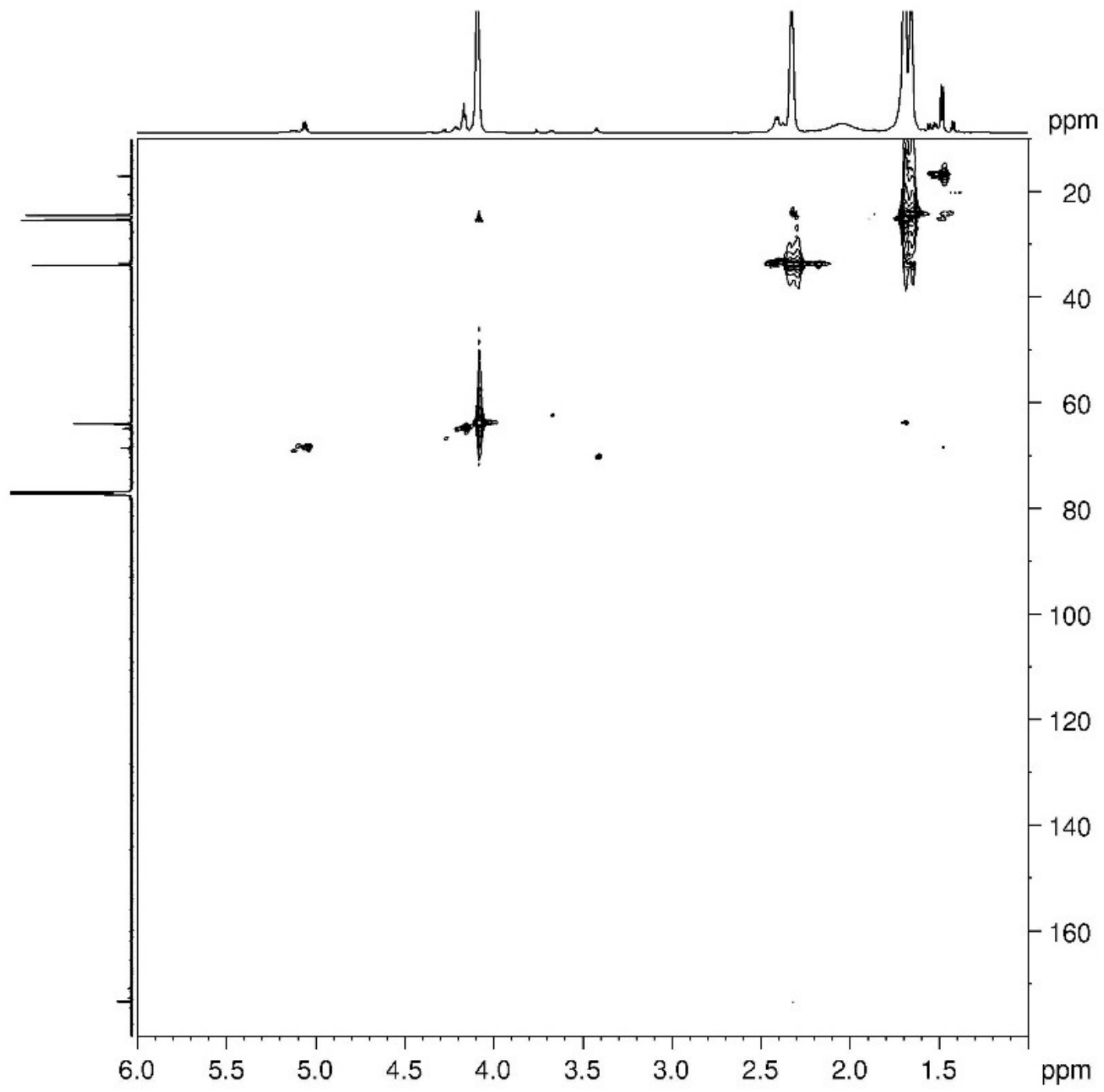

Figure S28. HSQC NMR spectrum (600 MHz, $CDCl_3$, 298 K) of poly(butylene adipate-*co*-lactate), PBALa.

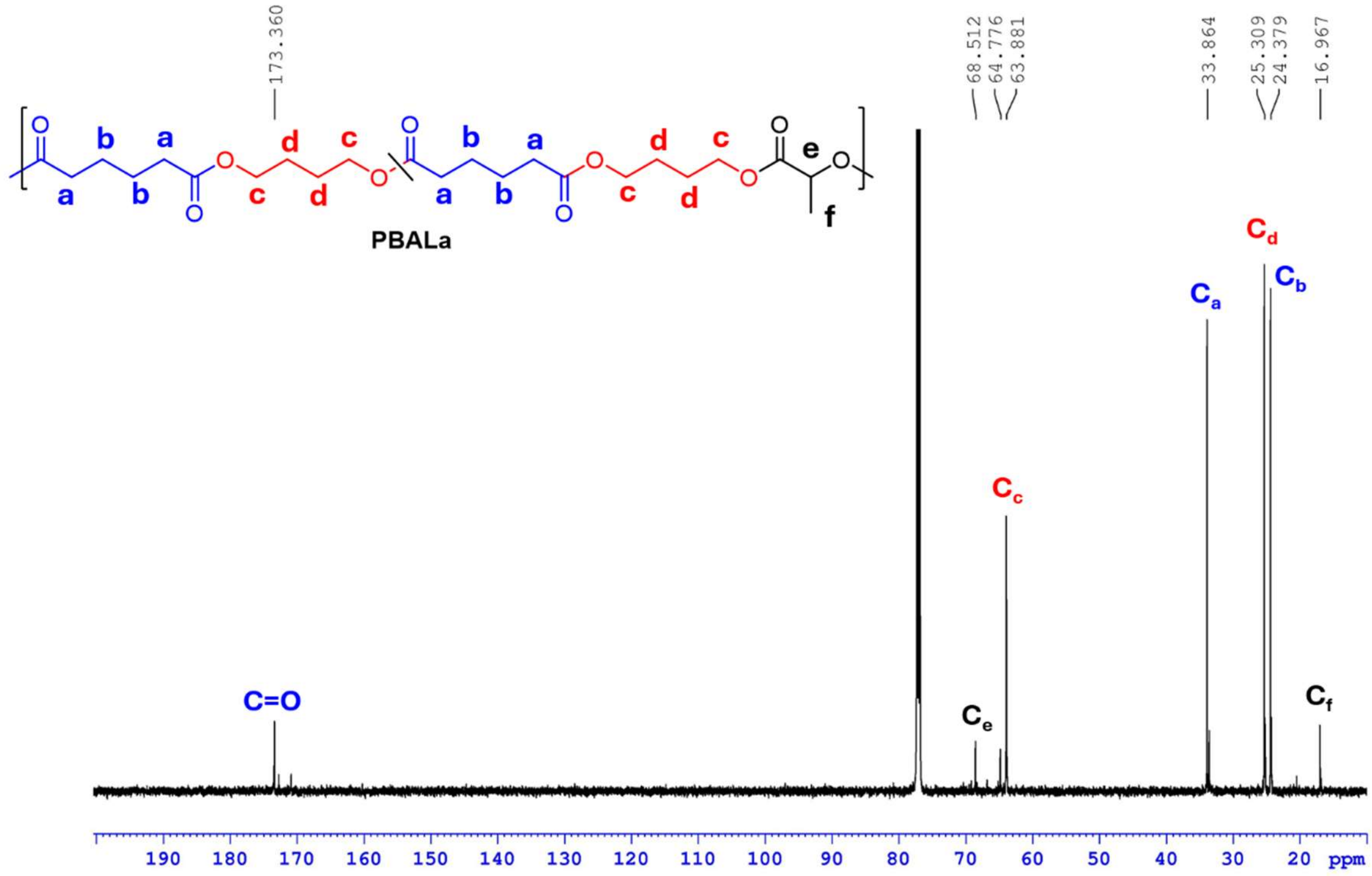

Figure S29. ^{13}C NMR spectrum (150 MHz, $CDCl_3$, 298 K) of poly(butylene adipate-*co*-lactate), PBALa.

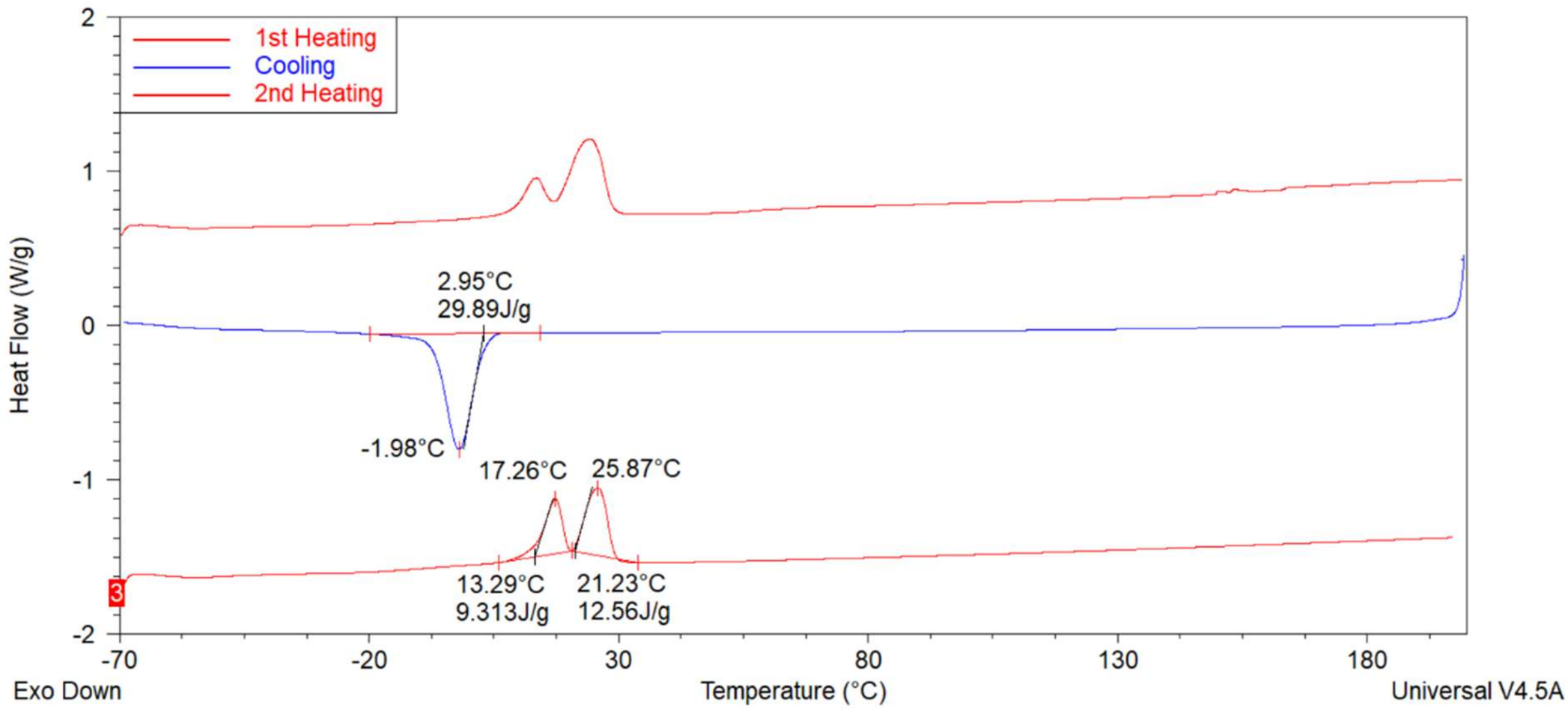

Figure S30. DSC of poly(butylene adipate-*co*-lactate), PBALa.

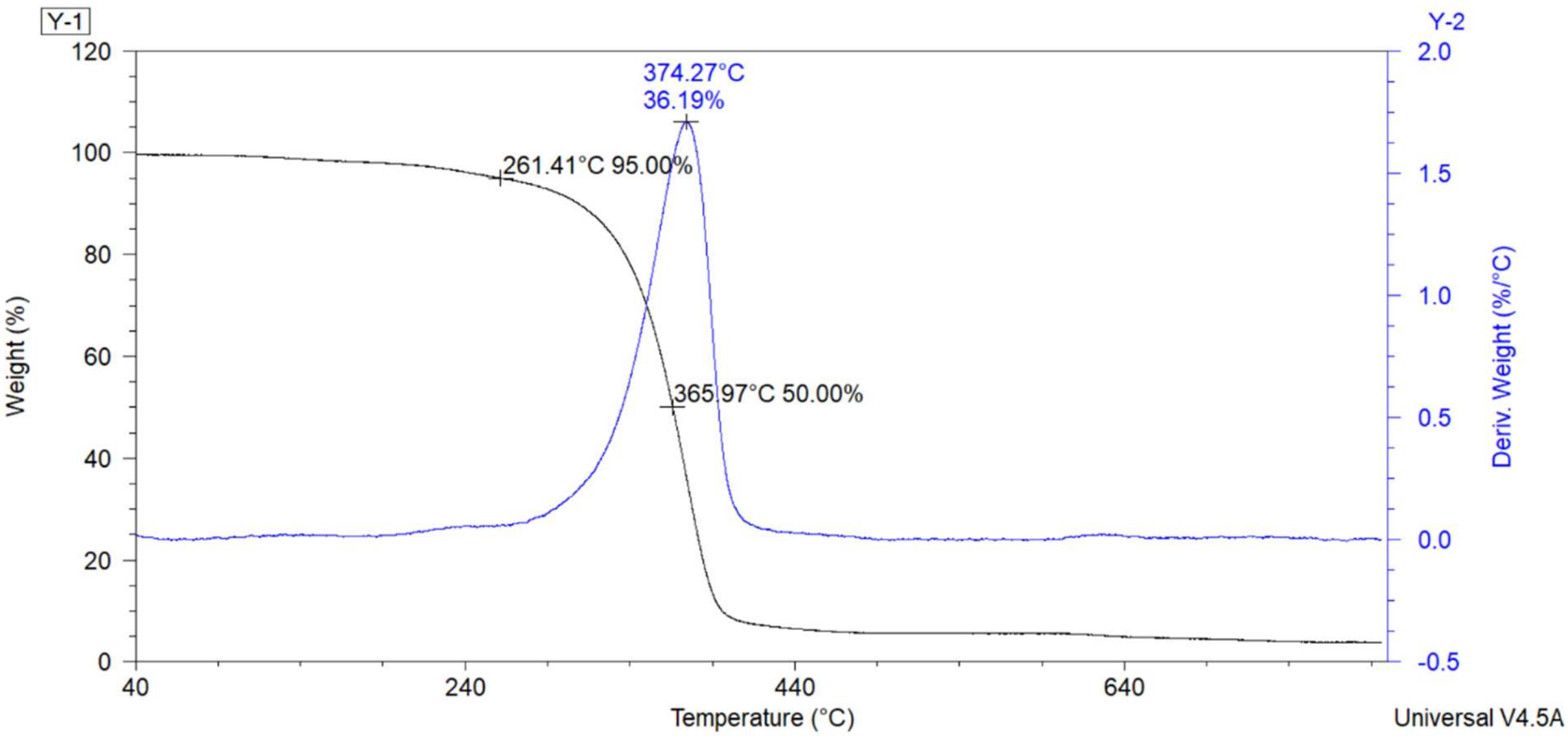

Figure S31. TGA of poly(butylene adipate-*co*-lactate), PBALa.

- *1,4-cis butene diol (cis-BD)*

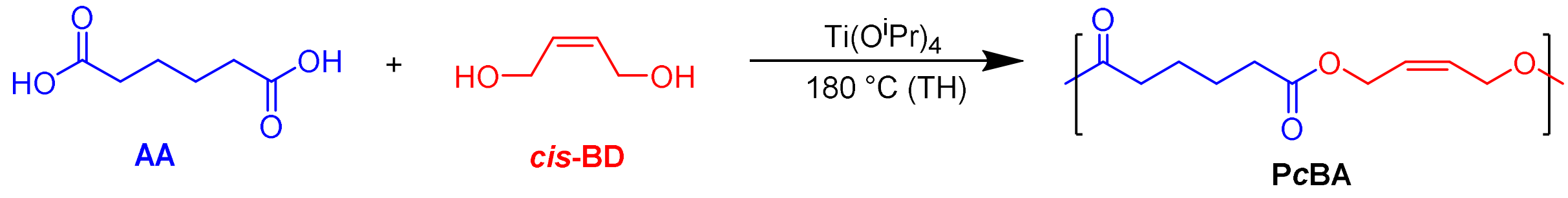

Scheme S5. Polycondensation reaction of the adipic acid (AA) and 1,4-*cis* butene diol (*cis*-BD).

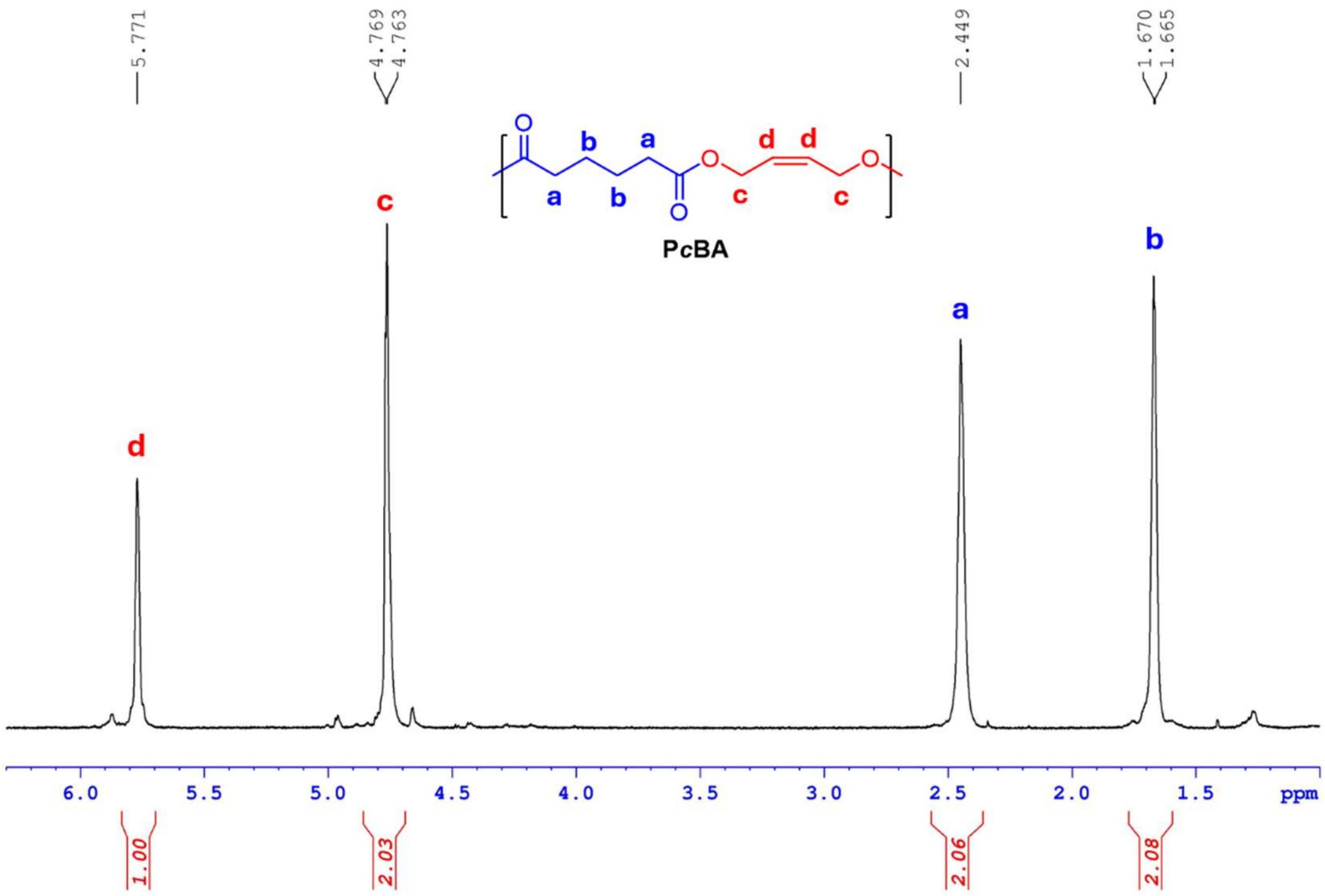

Figure S32. ^{1}H NMR spectrum (600 MHz, $CDCl_3$, 298 K) of poly(*cis*-butene) adipate, P*c*BA.

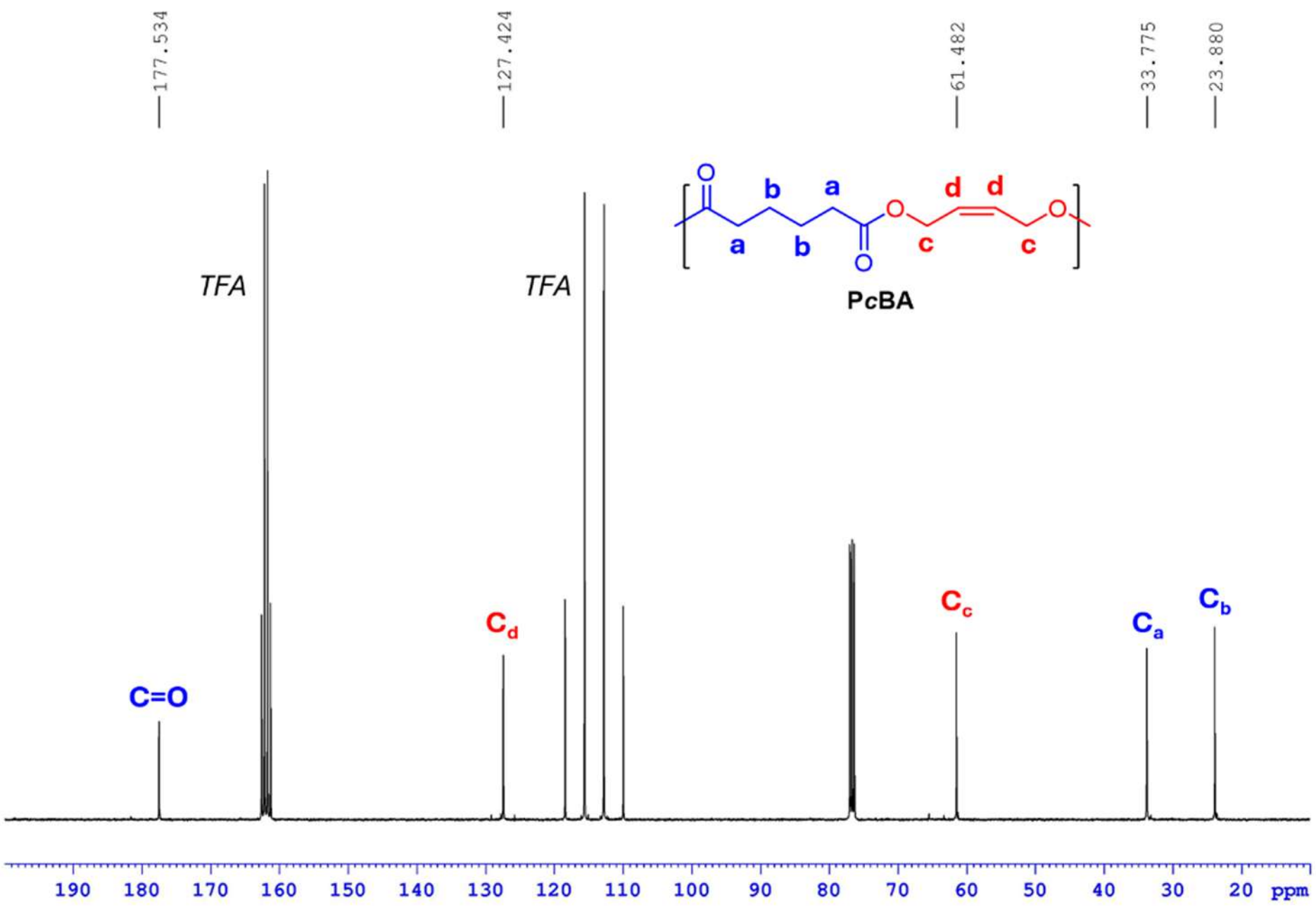

Figure S33. ^{1}H NMR spectrum (150 MHz, $CDCl_3$, 298 K) of poly(*cis*-butene) adipate, P*c*BA. *TFA= trifluoroacetic acid.*

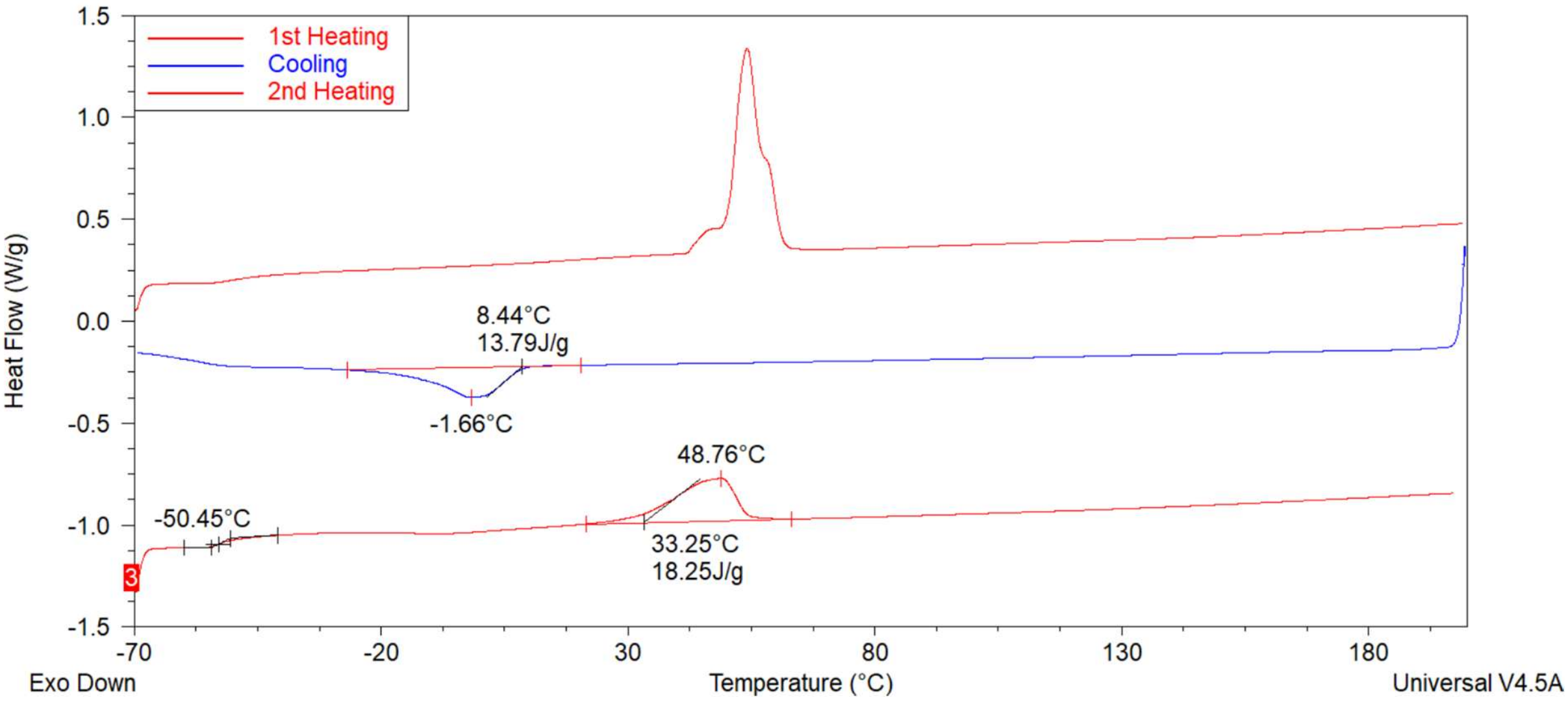

Figure S34. DSC of poly(*cis*-butene) adipate, P*c*BA.

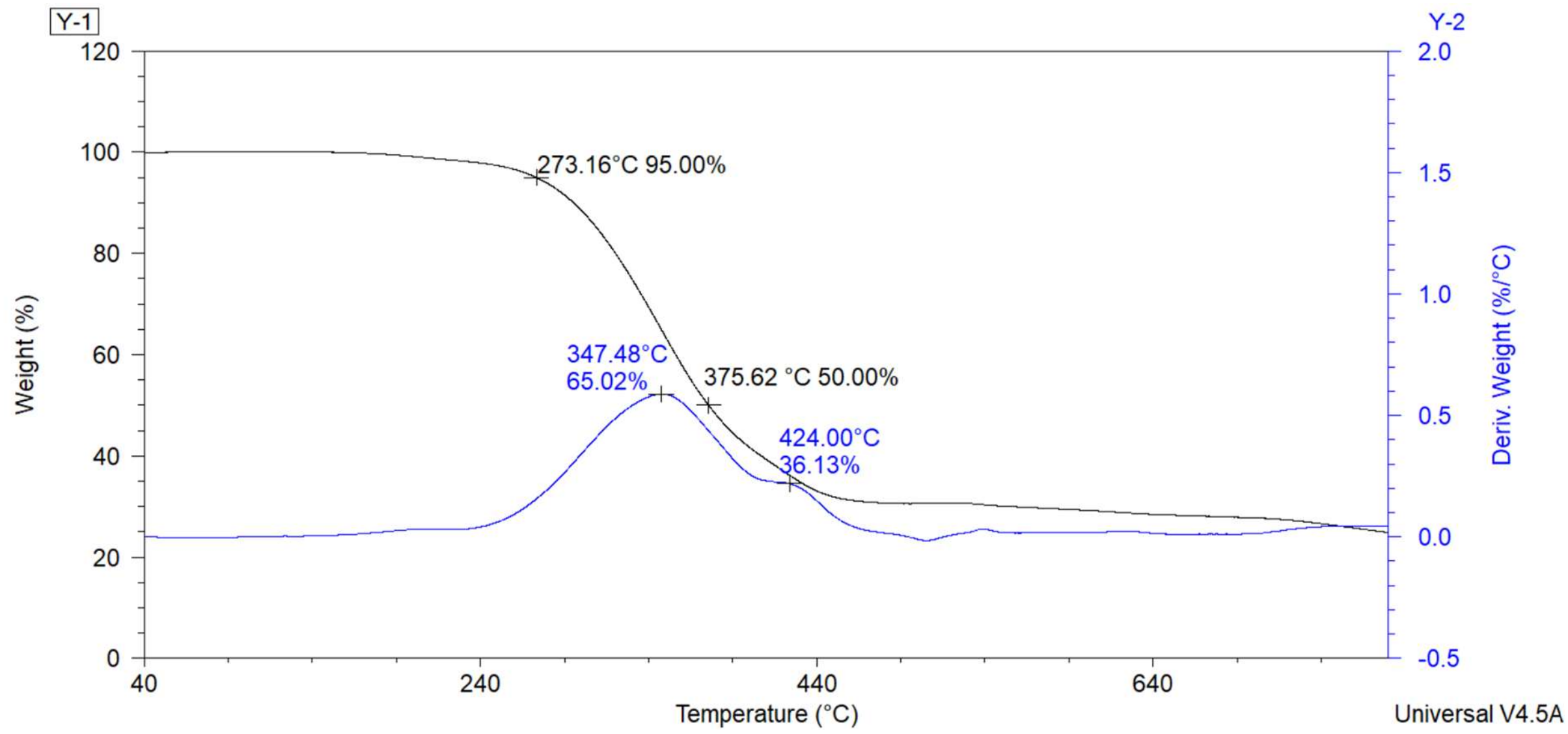

Figure S35. TGA of poly(*cis*-butene) adipate, P*c*BA.

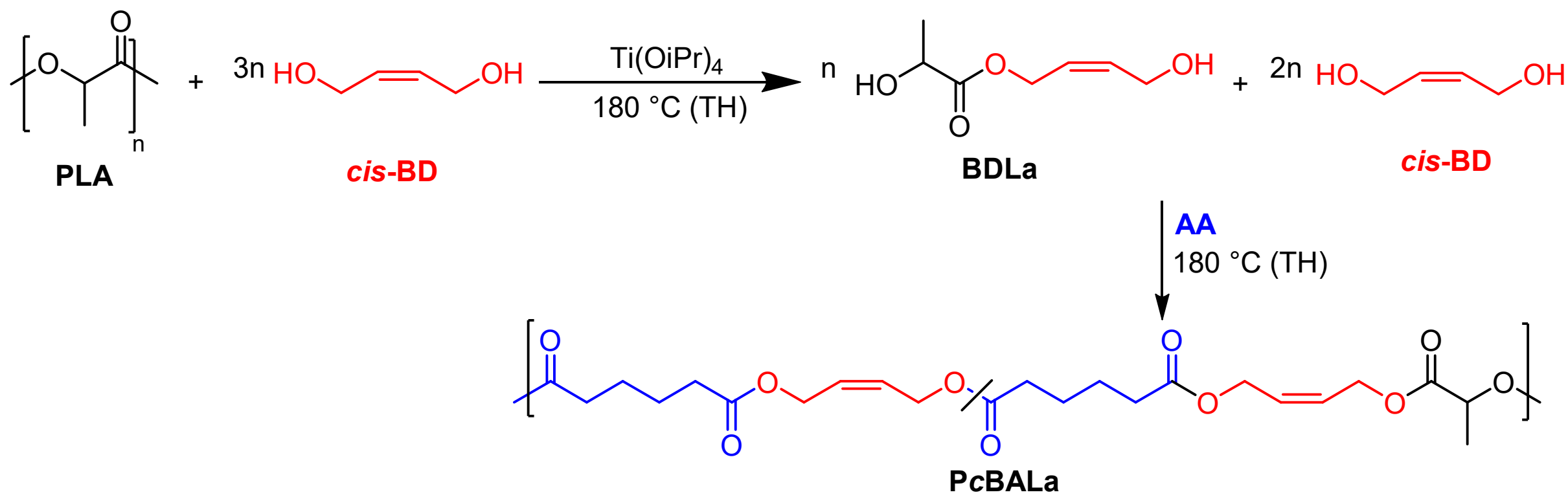

Scheme S6. Polycondensation reaction of the adipic acid (AA) with the mixture 1,4-*cis* butene diol (*cis*-BD) and 4-hydroxy-*cis*-butyl lactate (*c*BDLa).

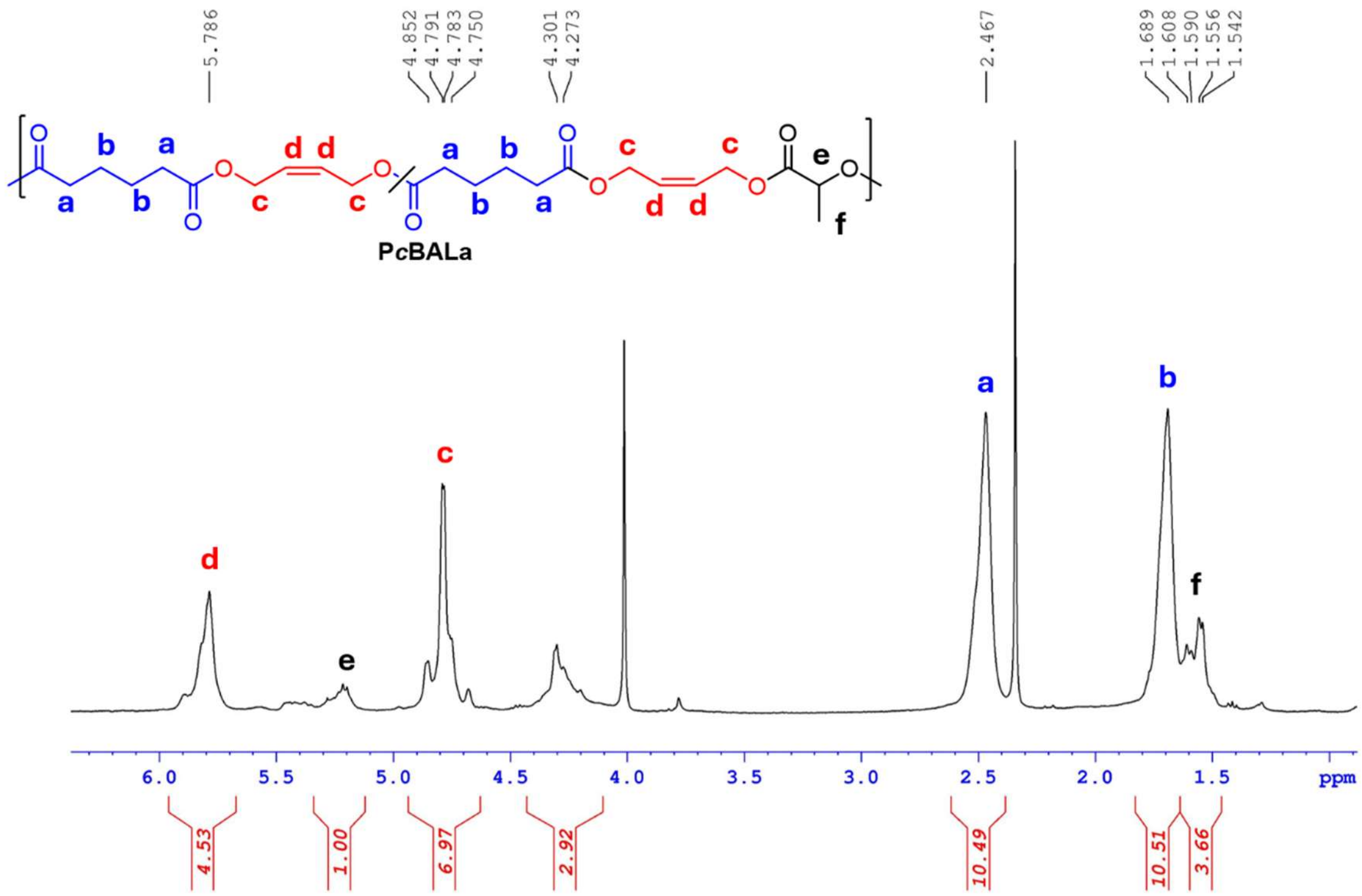

Figure S36. ^{1}H NMR spectrum (600 MHz, $CDCl_3$, 298 K) of poly(*cis*-butene adipate-*co*-lactate), P*c*BALa.

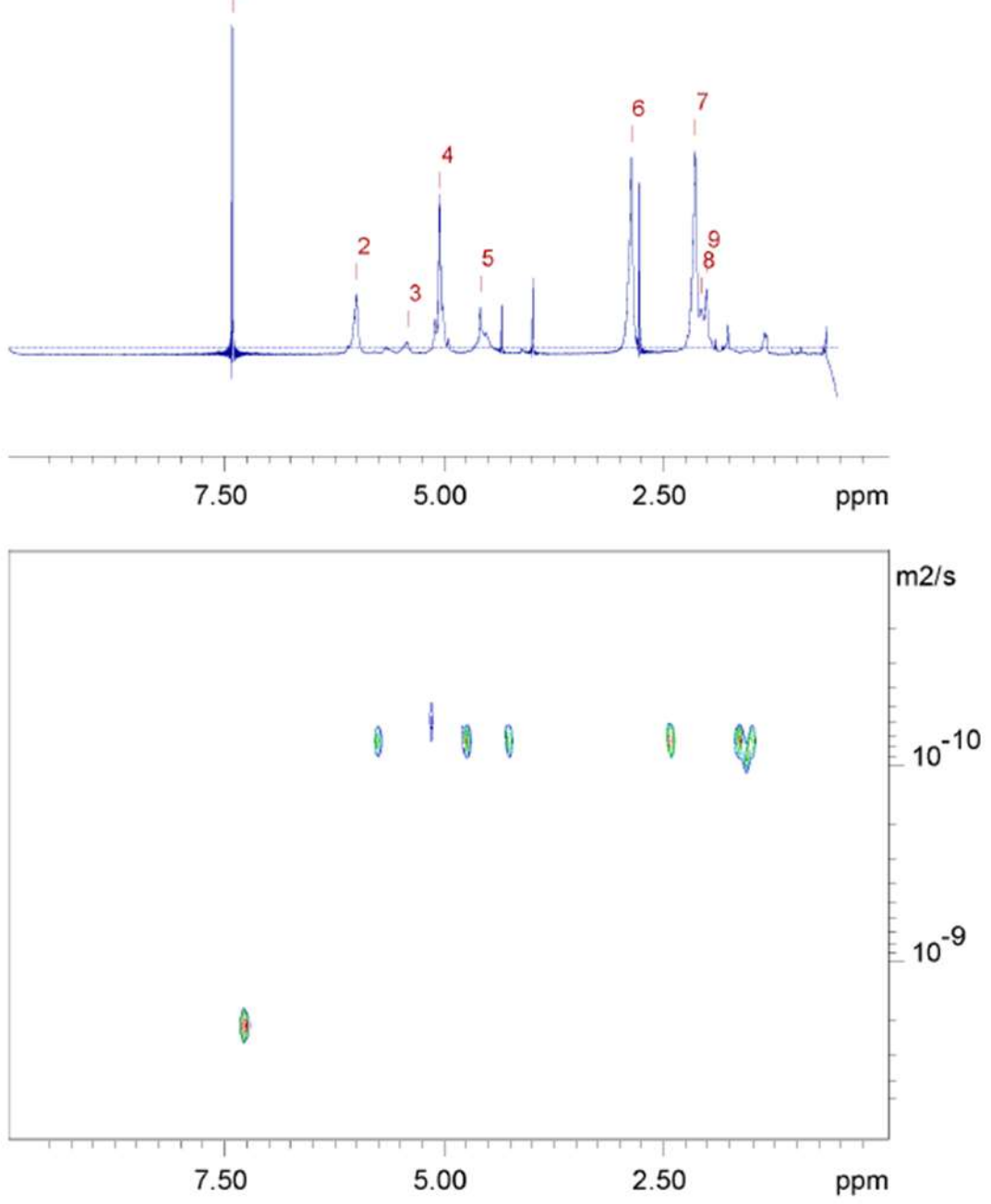

Figure S37. 2D DOSY NMR spectrum (600 MHz, $CDCl_3$, 298 K) of poly(*cis*-butene adipate-*co*-lactate), P*c*BALa.

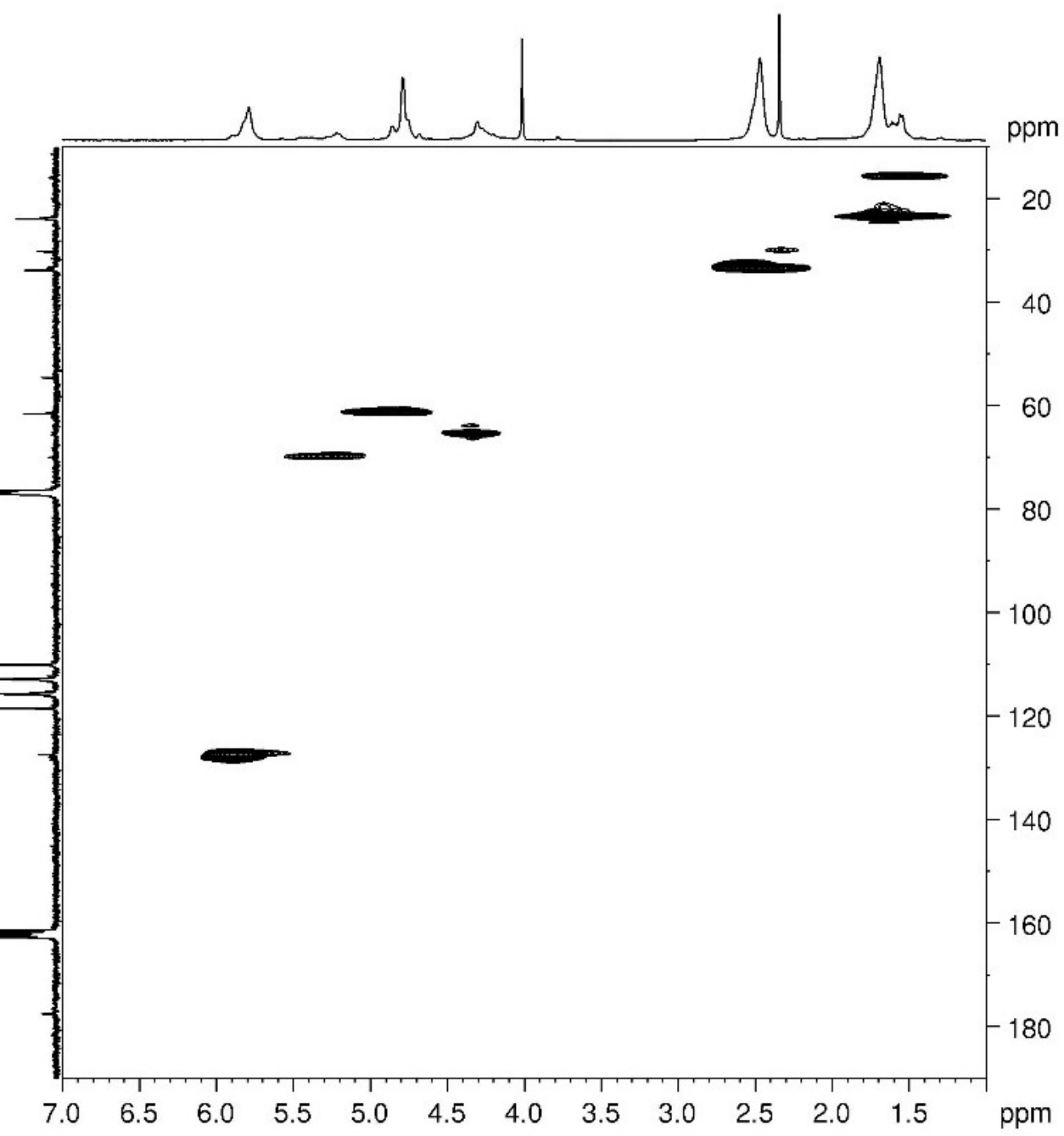

Figure S38. HSQC NMR spectrum (600 MHz, $CDCl_3$, 298 K) of poly(*cis*-butene adipate-*co*-lactate), P*c*BALa.

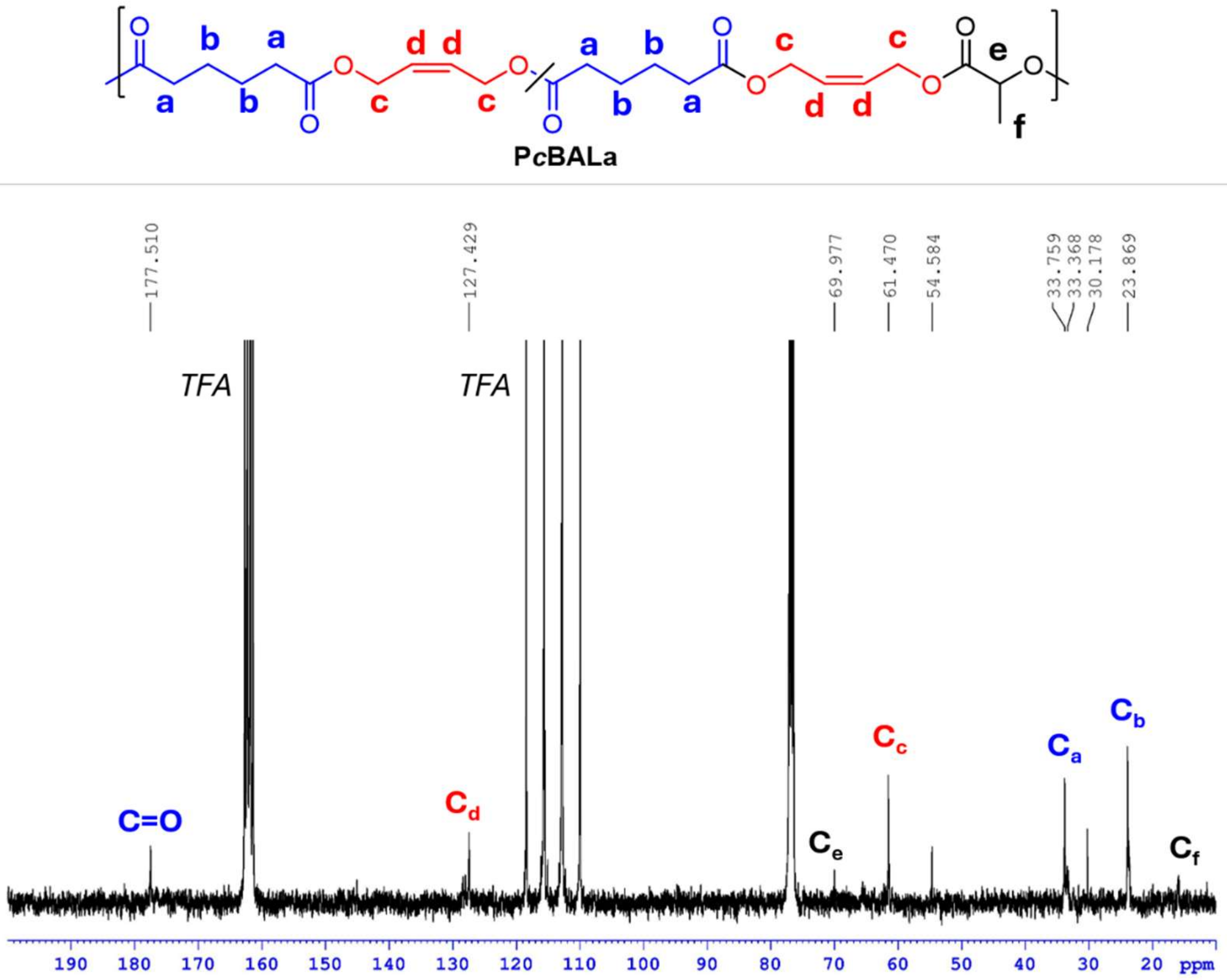

Figure S39. ^{13}C NMR spectrum (150 MHz, $CDCl_3$, 298 K) of poly(*cis*-butene adipate-*co*-lactate), P*c*BALa.
TFA= trifluoroacetic acid.

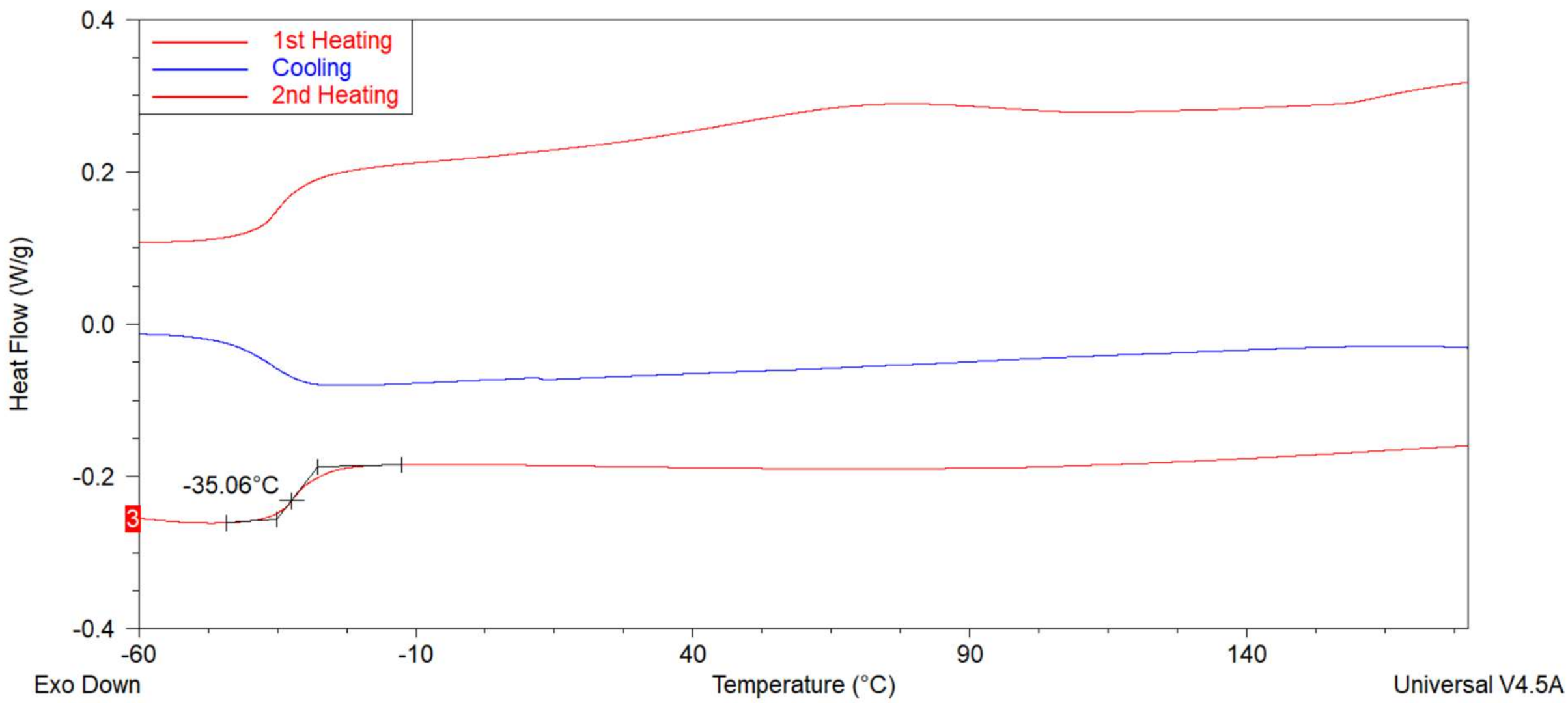

Figure S40. DSC of poly(*cis*-butene adipate-*co*-lactate), P*c*BALa.

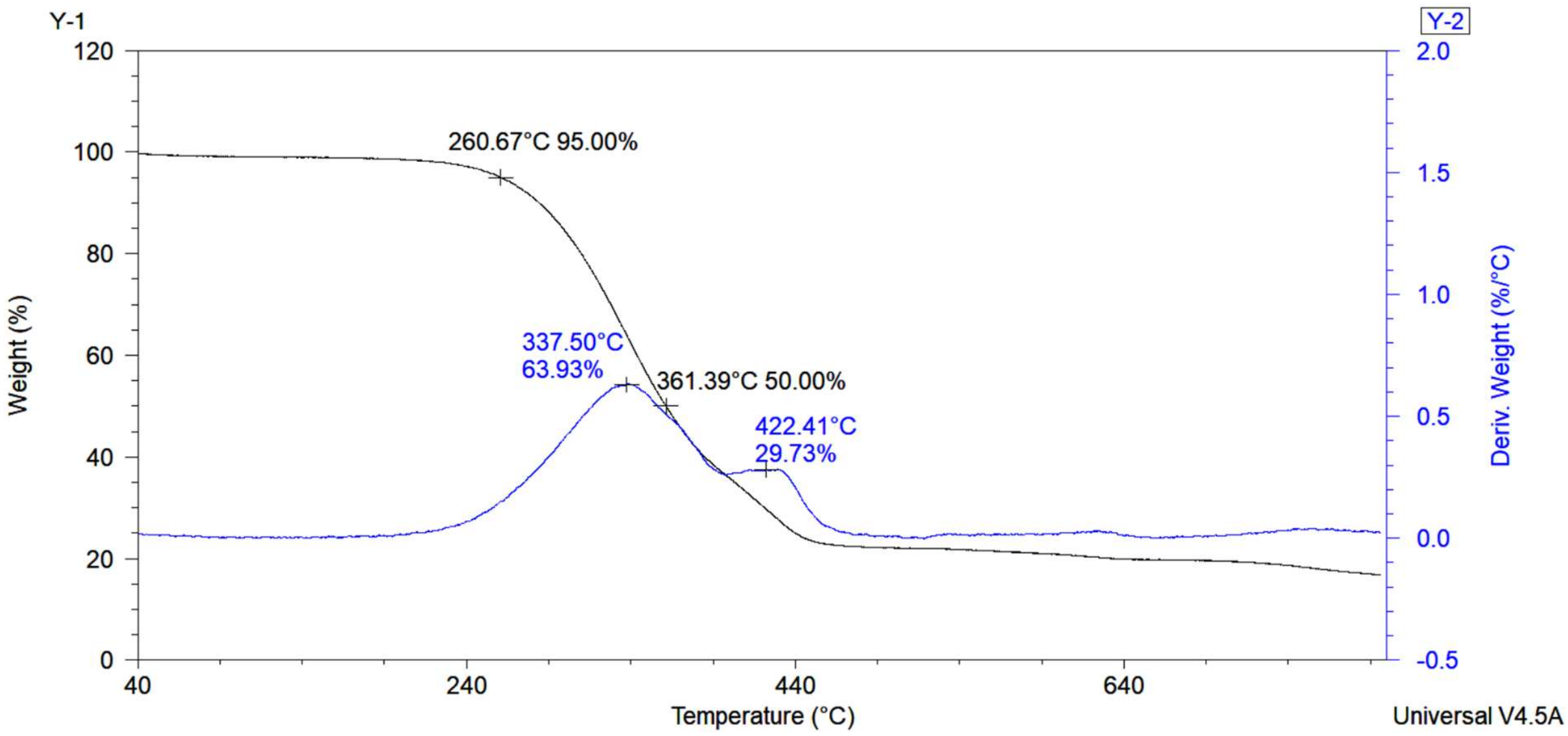

Figure S41. TGA of poly(*cis*-butene adipate-*co*-lactate), P*c*BALa.

[1] aF. Santulli, M. Lamberti, M. Mazzeo, *ChemSusChem* 2021, *14*, 5470-5475; bS. D'Aniello, S. Laviéville, F. Santulli, M. Simon, M. Sellitto, C. Tedesco, C. M. Thomas, M. Mazzeo, *Catalysis Science and Technology* 2022, *12*, 6142-6154; cF. Santulli, D. Pappalardo, M. Lamberti, A. Amendola, C. Barba, A. Sessa, G. Tepedino, M. Mazzeo, *ACS Sustainable Chemistry and Engineering* 2023, *11*, 15699-15709.
